# Calibrated Model Criticism Using Split Predictive Checks

**DOI:** 10.1080/01621459.2026.2649585

**Published:** 2026-06-11

**Authors:** Jiawei Li, Jonathan Hunter Huggins

**Affiliations:** a Department of Mathematics & Statistics, Boston University, Boston, MA; b Department of Mathematics & Statistics and Faculty of Computing & Data Sciences, Boston University, Boston, MA

**Keywords:** Bayesian model checking, Model misspecification, Posterior predictive checks, Predictive generalization, Uncertainty quantification

## Abstract

Assessing how well a Bayesian model generalizes to unobserved data is essential, yet existing general-purpose model checks are either not properly calibrated (as in posterior predictive checks) or fail to be sufficiently general for practical use (e.g., due to requiring model-specific derivations). We propose *split predictive checks (SPCs)* as a simple, general-purpose class of predictive checks that maintain the usability of posterior predictive checks while directly targeting predictive generalization. SPCs work by splitting the data into training and test subsets, then fitting the model to the former and evaluating predictive discrepancies on the latter. We develop an asymptotic theory for two variants—single SPCs and divided SPCs—and show that, unlike posterior predictive checks, both yield asymptotically calibrated (hence interpretable) *p*-values. Our results show that single SPCs work well at identifying substantial misspecification, while divided SPCs are sensitive even to subtle departures from modeling assumptions. Through simulation studies and real-data applications, we show that SPCs provide reliable, flexible, and computationally efficient assessments of Bayesian model fit, often revealing issues with predictive generalization missed by other predictive checks. Supplementary materials for this article are available online, including a standardized description of the materials available for reproducing the work.

## 1. Introduction

Assessing the fit of a Bayesian model is essential for understanding when inferences or predictions based on the fitted model can be trusted (Box 1980; Gelman et al. 2013; Blei 2014). An effective model assessment method should flag when the model fails to capture aspects of the data that the analyst considers important while accepting models that are adequate for the task. In addition, to support the iterative model building process that is possible with modern probabilistic programming systems such as BUGS, Stan, NIMBLE and PyMC (Lunn et al. 2009; de Valpine et al. 2015; Salvatier, Wiecki, and Fonnesbeck 2016; Carpenter et al. 2017; Bingham et al. 2019), a practical model check should be broadly usable without model-specific derivations. Existing approaches satisfy some of these goals, but none satisfy all: methods that are broadly applicable and computationally tractable lack reliable calibration, while methods with calibration guarantees require model-specific derivations or are computationally prohibitive (Gelfand, Dey, and Chang 1992; Bayarri and Berger 2000; Robins, van der Vaart, and Ventura 2000; Marshall and Spiegelhalter 2003; Johnson 2004; Hjort, Dahl, and Steinbakk 2006; Johnson 2007; Dahl, Gåsemyr, and Natvig 2007; Bayarri and Castellanos 2007; Yuan and Johnson 2011).

The most widely used tool in practice is the posterior predictive check (PPC; Guttman 1967; Rubin 1984; Gelman, Meng, and Stern 1996). Its appeal is clear: PPCs are general-purpose, easy to compute, and allow the analyst to tailor the check to features of the data that matter for the scientific question at hand. Conceptually, PPCs test whether the observed data look typical under the posterior predictive distribution conditioned on that data. This kind of check for *self-consistency* is perfectly sensible in applications where the primary concern is whether the posterior adheres to patterns already present in the data. However, when the goal is to assess a model’s ability to predict unobserved data, the double use of the observed data in PPCs becomes problematic because there is no guarantee that a fitted model that is self-consistent will generalize. Predictive checks that use some form of cross-validation aim to avoid double use of the data (Gelfand, Dey, and Chang 1992; Vehtari and Lampinen 2002). However, these methods can be computationally prohibitive (Marshall and Spiegelhalter 2003; Gronau and Wagenmakers 2019) and are not necessarily well-calibrated (Ambroise and McLachlan 2002; Cawley and Talbot 2010; Piironen and Vehtari 2017).

To address the limitations of existing model checks, we propose to use a predictive check that explicitly targets the fitted model’s predictive calibration on unobserved data by splitting the data into “train” and “test” sets. Hence, we call such methods *split predictive checks* (SPCs). Framing model checking to be about the unobserved data forces a more precise articulation of what type of generalization is required in a given application, with different applications naturally calling for different data-splitting schemes: prediction, extrapolation, hierarchical generalization, and temporal forecasting each impose distinct requirements. SPCs provide a unified framework that can accommodate these differences.

We develop an asymptotic theory for two SPC variants: *single SPCs* and *divided SPCs*. Our results show that both SPCs provide asymptotically well-calibrated checks with complimentary strengths. Single SPCs are applicable even in small-data regimes and is sensitive to substantial misspecification, while divided SPCs are applicable at larger sample sizes and can detect more subtle departures from modeling assumptions. Through simulations and real-data examples, we show how SPCs retain the ease and speed of PPCs while providing calibrated and application-aligned assessments of Bayesian model fit and generalization ability.

### Notation.

For a sequence $a_{1},a_{2},\ldots ,a_{N}$ (where a may be replaced with any other letter-like symbol), we define $a:=a_{N}=\left(a_{1},a_{2},\ldots ,a_{N}\right)$. For a vector $v\in R^{d}$, let $\|v{\|}_{2}$ denote the usual Euclidean norm. For a set A, denote the interior of the set as Int(A). We write f(N)=O(g(N)) if $lim\underset{N\to \infty }{sup}\;|f(N)/g(N)|<\infty$. We write $z_{\alpha }$ to denote the αth quantile of standard normal distribution. We write $\overset{𝒟\;}{\to }$ to denote convergence in distribution and $\overset{P\;}{\to }$ to denote convergence in probability.

## 2. Background

### 2.1. Approaches to Bayesian Model Assessment and Model Checking

The philosophy behind Bayesian model *assessment* and Bayesian model *checking* has been explored extensively (Gustafson 2001; Presnell and Boos 2004; Stern and Sinharay 2005; Gelman et al. 2013; Sprenger 2013). Broadly, Bayesian model assessment treats the model as an object to be tested, often against correctness in a frequentist sense, while Bayesian model checking focuses on diagnosing where and how the model departs from important features of the data. Classical goodness-of-fit ideas motivate one set of Bayesian assessment tools such as $\chi^{2}$-type tests (Johnson 2004, 2007; Yuan and Johnson 2011). These approaches produce asymptotically uniform *p*-values when the model is correctly specified, which aids in interpretability. However, they require model-specific derivations and offer limited flexibility for probing user-specified aspects of the data.

Bayesian model checking methods, on the other hand, can be formulated as assessing how well the model fits the data *in particular directions* selected by the analyst. This perspective underlies a wide range of test statistic–based checks (Gelman, Meng, and Stern 1996; Berkhof, Van Mechelen, and Hoijtink 2000; Ibrahim, Chen, and Sinha 2001; Steiger 2007; Czado, Gneiting, and Held 2009; Wang, Kasprzak, and Huggins 2023). The most common class of Bayesian model checking methods are called *predictive checks* (Guttman 1967; Box 1980; Rubin 1984; Gelfand, Dey, and Chang 1992; Bayarri and Berger 2000; Robins, van der Vaart, and Ventura 2000; Marshall and Spiegelhalter 2003; Hjort, Dahl, and Steinbakk 2006; Dahl, Gåsemyr, and Natvig 2007; Bayarri and Castellanos 2007; Gåsemyr and Scheel 2019; Moran, Blei, and Ranganath 2024). This approach requires the analyst to define a statistic that captures features they consider scientifically meaningful. The model is assessed by comparing the observed value of that statistic to its distribution under some version of the model’s predictive mechanism.

To formalize predictive checking, consider a model $\left\{P_{\theta }\;:\theta \in \Theta \right\}$ and denote the density of $P_{\theta }$ with respect to some reference measure λ by $p_{\theta }$. Assume random variables $X_{1},\ldots ,X_{N}\overset{\text{iid}}{∼}P_{\circ }$, where $X_{n}\in X$ and $x_{i}$ is a realization of $X_{i}$. Define $X\;:=\left(X_{1},\ldots ,X_{N}\right)$, $x\;:=\left(x_{1},\ldots ,x_{N}\right)$ and, with a slight abuse of notation, $p_{\theta }(x)\;:=\prod_{n=1}^{N}p_{\theta }\left(x_{n}\right)$. Let $\theta_{⋆}:={sup}_{\theta \in \Theta }\;E\left\{log\;p_{\theta }\left(X_{1}\right)\right\}$ denote the parameter that minimizes the Kullback–Leibler divergence between the data generating distribution and the model family; we assume throughout that $\theta_{⋆}$ exists and is unique. Predictive checks rely on a family of statistics ${\left(T_{N}:X^{N}\to R\right)}_{N\in N}$ indexed by the dataset size. The analyst can determine which aspects of the data the predictive check will validate via the choice of $T=T_{N}$. The observed value of the statistic is compared to the statistic evaluated on data sampled from a “null distribution” with density m. Let $\Pi_{0}$ denote the prior distribution on Θ. Standard choices for m include the prior predictive density $m_{\text{prior}}\left(x_{\text{pred}}\right)\;:=\int p_{\theta }\left(x_{\text{pred}}\right)\Pi_{0}(d\theta )$ (Box 1980) and the posterior predictive density $m_{\text{post}}\left(x_{\text{pred}}\right)\;:=\int p_{\theta }\left(x_{\text{pred}}\right)\Pi (d\theta |x)$ (Guttman 1967; Rubin 1984), where $\Pi \left(d\theta |x\right):=p_{\theta }(x)\Pi_{0}(d\theta )/m_{\text{prior}}(x)$ is the posterior distribution. The p-value for the predictive check is given by $p(x)\;:={Pr}_{X_{\text{pred}}∼m}\left\{T\left(X_{\text{pred}}\right)\geq T(x)\right\}$, where the superscript m denotes the distribution of $Y=\left(Y_{1},\ldots ,Y_{N}\right)$. Given any p-value p(x), define the two-sided version by $p_{2}(x)=2\;min\{p(x),1-p(x)\}$.

Some predictive checks require elaborations on the basic definition. For example, the statistic can also be allowed to depend on the model parameter, in which case it is of the form T(x,θ) and the null distribution m is a joint distribution on X×Θ (Gelman, Meng, and Stern 1996; Mimno and Blei 2011). In the posterior predictive case, the null density would be $m_{\text{post}}\left(x_{\text{pred}},\theta \right)\;:=p_{\theta }\left(x_{\text{pred}}\right)\pi (\theta |x)$, where π is the density of with respect to λ.

A second example is the cross-validated predictive check, where one p-value $p_{n}$ is computed for each observation: $p_{n}(x)\;:={Pr}_{X_{\text{pred},n}∼m_{\text{post}}^{-n}}\left\{T\left(X_{\text{pred},n}\right)\geq T\left(x_{n}\right)\right\}$, where

$$m_{\text{post}}^{-n}\left(x_{\text{pred},n}\right)\;:=\int p_{\theta }\left(x_{\text{pred},n}\right)\Pi \left(d\theta |x^{-n}\right)$$

is the posterior predictive density of the nth observation conditional on $X^{-n}:=\left(X_{1},\ldots ,X_{n-1},X_{n+1},\ldots ,X_{N}\right)$, the data without the nth observation (Marshall and Spiegelhalter 2003). Finally, rather than computing p-values, predictive checks can be also performed graphically (Gelman, Meng, and Stern 1996).

### 2.2. Limitations of Existing Predictive Checks

Posterior predictive checks (PPCs) owe much of their popularity to their simplicity and generality. They require only posterior draws and a user-chosen test statistic, making them easy to compute and widely applicable. Conceptually, PPCs verify the *self-consistency* of the fitted model by evaluating whether the observed data look typical under the posterior predictive distribution. This is entirely sensible when the goal is *explanatory adequacy*—that is, determining whether the fitted model captures patterns already present in the observed data (Shmueli 2010).

However, many scientific applications are ultimately concerned with prediction (Shmueli 2010; Efron 2020). In predictive settings, the double use of the observed data in PPCs becomes difficult to justify. Because the same data inform the posterior and are used to evaluate the model, PPCs inherently assess how well the model reproduces the data it was fit to, not how well it generalizes. Hence, a model can pass a PPC while still producing poor predictions for future or held-out observations (Robins, van der Vaart, and Ventura 2000).

This broader issue relates to, but is not reducible to, calibration. Posterior predictive *p*-values are typically conservative and nonuniform, which complicates their interpretation. But even a perfectly calibrated PPC would still evaluate the wrong target if the inferential goal concerns predictive performance rather than retrospective fit. Methods that attempt to avoid double use of the data (for example, cross-validated predictive checks; Gelfand, Dey, and Chang 1992; Marshall and Spiegelhalter 2003) address part of the problem by separating the data used to fit the model from the data used to check it. Yet these approaches can be computationally prohibitive, and the resulting *p*-values don’t provide a concise summary of model fit (Ambroise and McLachlan 2002; Cawley and Talbot 2010; Piironen and Vehtari 2017).

Finally, approaches that do offer calibrated predictive assessments often require model-specific derivations and are thus not general-purpose (Bayarri and Berger 2000; Dahl, Gåsemyr, and Natvig 2007; Bayarri and Castellanos 2007). So, in practice, analysts must choose between methods that test the wrong notion of fit for prediction-focused applications, methods that are theoretically sound but model-specific, and methods that are conceptually more appropriate but lack a clear interpretation in terms of overall model fit. These limitations highlight the need for predictive checks that (i) directly target a model’s ability to generalize to unobserved data, (ii) remain easy to implement across a wide class of models, and (iii) provide an interpretable summary of fit. Split predictive checks aim to provide exactly this combination.

## 3. Split Predictive Checks

We introduce split predictive checks (SPCs) as a general-purpose method to assess a fitted model’s predictive capabilities on unobserved data. SPCs complement the strengths of PPCs by computing predictive *p*-values using a data split: the model is fit to an observed “training” subset and evaluated on a held-out “test” subset. This separation avoids the double use of data inherent in PPCs, while retaining the computational efficiency and ease of implementation that make PPCs appealing in practice. Proofs of all theoretical results can be found in the supplementary materials.

### 3.1. Single SPCs

We start with the simplest possible SPC, which we call the *single* SPC. For a given proportion q∈(0,1), we split the data into two disjoint subsets: the observed data $X_{\text{obs}}$ of size $N_{\text{obs}}\;:=\lceil qN\rceil$ and the held-out data $X_{\text{ho}}$ of size $N_{ho}:=N-N_{\text{obs}}$. The *single q-SPC* p-*value* is defined as

(1) $$p_{SPC}(X)\;:=\underset{X_{\text{pred}}∼m_{\text{post}}^{SPC}}{Pr}\left\{T_{N_{ho}}\left(X_{\text{pred}}\right)\geq T_{N_{ho}}\left(X_{ho}\right)\right\},$$

where $m_{\text{post}}^{SPC}\;:=m_{\text{post}}\left(X_{\text{pred}}|X_{\text{obs}}\right)\;:=\int p_{\theta }\left(X_{\text{pred}}\right)\Pi \left(d\theta |X_{\text{obs}}\right)$ denotes the distribution of $X_{\text{pred}}$. Here we consider $p_{\text{SPC}}(X)$ as a random variable with $p_{\text{SPC}}(x)$ as its realization. Single SPCs inherit the “black-box” nature of general predictive checks as well as the flexibility provided to the analyst via the choice of statistic. Since only one additional posterior distribution must be computed, and it is on a smaller dataset, the additional computation required to obtain the single SPC *p*-value is relatively modest.

We first show that single q-SPC p-values are asymptotically uniform (i.e., well-calibarted) under the well-specified model and characterize their asymptotic power. The assumptions required for our result are relatively mild, and stated formally in Appendix A. The first key assumption is that the asymptotic mean $v(\theta )\;:=\underset{N\to \infty }{lim}\;\int T_{N}(x)P_{\theta }(dx)$ and asymptotic variance $\sigma^{2}(\theta )\;:=\underset{N\to \infty }{lim}\;\int T_{N}^{2}(x)P_{\theta }(dx)-\nu^{2}(\theta )$ exist. In addition, the derivative of the mean $\dot{v}(\theta )\;:=\frac{\partial \nu (\theta )}{\partial \theta }$ must exist at $\theta =\theta_{⋆}$. The second important assumption requires the scaled test statistic to satisfy a central limit theorem: for $Y_{1},\ldots ,Y_{N}\overset{\text{iid}}{∼}P_{\theta }$,

$$\frac{N^{1/2}\left\{T_{N}\left(Y_{N}\right)-v(\theta )\right\}}{\sigma (\theta )}\overset{𝒟\;}{\to }𝒩(0,\;1).$$

Our final assumption requires asymptotic normality of the maximum likelihood estimator $\overset{ˆ}{\theta }(X)\;:=arg{max}_{\theta \in \Theta }\;p_{\theta }(X)$ and the posterior Π(dθ∣X).

Denote the information matrices under the data distribution by $J_{⋆}:=-E_{P_{o}}\left[\nabla^{2}log\;p_{\theta_{⋆}}\left(X_{1}\right)\right]$ and $\Sigma_{⋆}:={cov}_{P_{o}}\left[\nabla \;log\;p_{\theta_{⋆}}\left(X_{1}\right)\right]$. Denote the asymptotic means and standard deviations of the test statistic under the data distribution by, respectively, $v_{\circ }\;:={lim}_{N\to \infty }\;\int T_{N}(x)P_{\circ }(dx)$ and $\sigma_{\circ }^{2}\;:={lim}_{N\to \infty }\;\int T_{N}^{2}(x)P_{\circ }(dx)-v_{\circ }^{2}$.

#### Theorem 1.

Under Assumptions 2–4, the following hold for any α∈(0,1):

1. If $\nu_{\circ }=\nu \left(\theta_{⋆}\right)$, then
$$Pr\left[p_{SPC}(X)<\alpha \right]\overset{P\;}{\to }1-\Phi \left(\frac{z_{1-\alpha }}{\rho }\right)$$
  where
$$\rho^{2}:=\frac{q\sigma_{\circ }^{2}+(1-q)\dot{v}{\left(\theta_{⋆}\right)}^{⊤}J_{⋆}^{-1}\Sigma_{⋆}J_{⋆}^{-1}\dot{v}\left(\theta_{⋆}\right)}{q\sigma {\left(\theta_{⋆}\right)}^{2}+(1-q)\dot{v}{\left(\theta_{⋆}\right)}^{⊤}J_{⋆}^{-1}\dot{v}\left(\theta_{⋆}\right)}.$$
  In particular, if the model is correctly specified, then $Pr\left[p_{\text{SPC}}(X)<\alpha \right]\overset{P\;}{\to }\alpha$.
2. If $\nu_{\circ }<\nu \left(\theta_{⋆}\right)$, then $Pr\left[p_{\text{SPC}}(X)>1-\alpha \right]\overset{P\;}{\to }1$.
3. If $\nu_{\circ }>\nu \left(\theta_{⋆}\right)$, then $Pr\left[p_{\text{SPC}}(X)<\alpha \right]\overset{P\;}{\to }1$.

Theorem 1 shows that the asymptotic behavior of single SPC *p*-values is impacted by three factors. The first factor is whether the model can match the mean of the test statistic under the data distribution; that is, whether $v_{\circ }=v\left(\theta_{⋆}\right)$. If $v_{\circ }\neq v\left(\theta_{⋆}\right)$, then the model is misspecified, and Parts 2 and 3 state that the single SPC *p*-value will have asymptotic power 1. We refer the case where the model cannot match the mean of the statistic as *substantial misspecification*.

However, when $v_{o}=v\left(\theta_{⋆}\right)$, the behavior of the single SPC *p*-value depends on the quality of the uncertainty quantification of *(i)* the test statistic and *(ii)* the model parameters, which are summarized by the quantity $\rho^{2}$. The numerator of $\rho^{2}$ is a mixture of the asymptotic variance of the test statistic under the data distribution, $\sigma_{o}^{2}$, and a more complicated term, $\dot{v}{\left(\theta_{⋆}\right)}^{⊤}J_{⋆}^{-1}\Sigma_{⋆}J_{⋆}^{-1}\dot{v}\left(\theta_{⋆}\right)$. This latter term can be interpreted as the asymptotic variance of the MLE, $J_{⋆}^{-1}\Sigma_{⋆}J_{⋆}^{-1}$, in the direction $v\left(\theta_{⋆}\right)$ is changing most quickly (i.e., $\dot{v}\left(\theta_{⋆}\right)$). The denominator has a similar form, but now $\sigma_{\circ }^{2}$ is replaced by the test statistic asymptotic variance under the model and the second term now quantifies the asymptotic posterior variance $J_{⋆}^{-1}$ in the direction of $\dot{v}\left(\theta_{⋆}\right)$.

**Figure 1.**
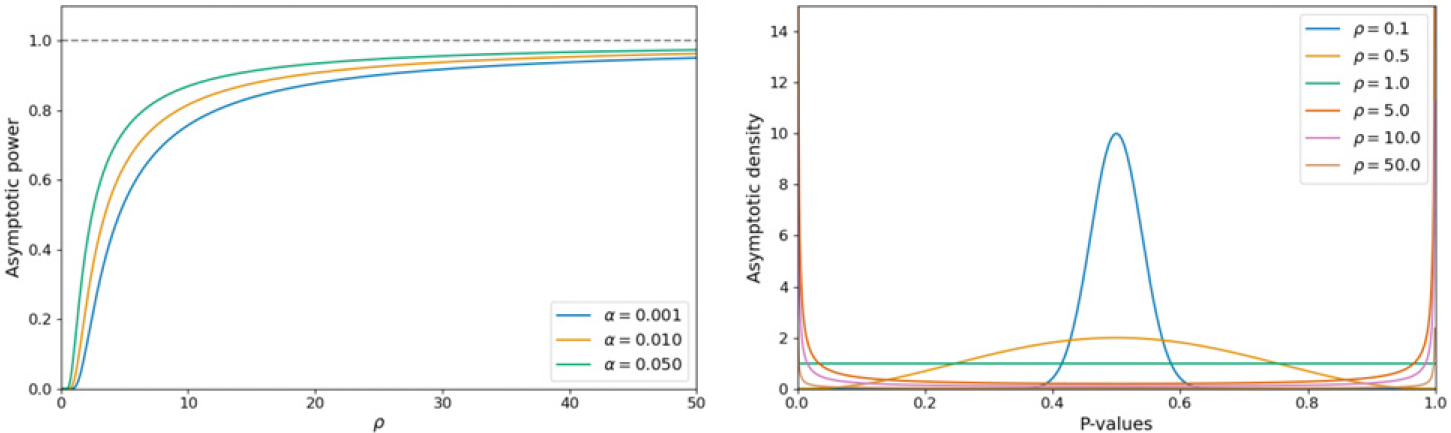
Left: The asymptotic power of single SPCs against ρ using a two-sided *p*-value with fixed αϵ{0.001,0.01,0.05}. The horizontal dashed line indicates power 1. Right: The asymptotic density of the single SPC *p*-values for different values of ρ.

If the model *under*estimates the two forms of uncertainty, then $\rho^{2}>1$. In this case, the power of a test using the single SPC *p*-value is greater than α, and converges to 1 as $\rho^{2}\to \infty$. Thus, the greater the underestimation of uncertainty, the greater the probability of obtaining a *p*-value close to 0 or 1. When the model correctly quantifies both forms of uncertainty, which includes the well-specified case, $\rho^{2}\;=\;1$ and the *p*-value is uniform. Hence, single SPCs are asymptotically uniform under correct specification. When the model *over*estimates the two forms of uncertainty, $\rho^{2}\;<\;1$. In this case the *p*-values concentrate away from 0 and 1. In this scenario the single SPC *p*-value is not useful for detecting model misspecification. How the asymptotic power and *p*-value distribution changes as a function of ρ are illustrated in Figure 1.

We refer to the case where the model can match the mean of the statistic as *mild-to-moderate misspecification*, with the exact degree of moderation quantified by the magnitude ρ. So, as a slogan, we can summarize Theorem 1 as showing that single SPCs will be effective at detecting moderate-to-substantial misspecification. It will depend on the use-case whether it is desirable that single SPCs fail to detect *mild misspecification*, which correspond to the case where ρ is not too large in magnitude. A cutoff of ρ≤3 to 5, for example, corresponds to a model that either overestimates or modestly underestimates uncertainty.

#### Example 1.

Consider a Gaussian location model with known variance $\sigma^{2}$ and the parameter of interest is the mean θ:

(2) $$X_{i}\overset{\text{iid}}{∼}𝒩\left(\theta ,\sigma^{2}\right),\;\theta ∼𝒩\left(\mu ,\tau^{2}\right).$$

Assume the data-generating distribution is $P_{\circ }=𝒩\left(\theta_{\circ },\sigma_{\circ }^{2}\right)$, so the model is misspecified if $\sigma \neq \sigma_{o}$. If we use the (sufficient) mean statistic $T_{N}\left(X_{N}\right)=\overset{‾}{X}\;:=\frac{1}{N}\sum_{i=1}^{N}X_{i}$, then $v_{\circ }=v\left(\theta_{⋆}\right)=\theta_{⋆}$. It follows from Part 1 of Theorem 1 that the asymptotic power of the two-sided single q-SPC converges to $2\Phi (z_{\frac{\alpha }{2}}/\rho )$ with $\rho =\sigma_{o}/\sigma$. Thus, the single q-SPC will be effective at detecting misspecification when the true variance is substantially underestimated (i.e., $\sigma_{o}^{2}/\sigma^{2}\gg 1$). However, when $\sigma_{o}^{2}/\sigma^{2}$ is close to or less than 1, the single q-SPC test will fail to reject the model.

#### Remark 1 (Extension to realized discrepancies).

While our focus is on the case of statistics depending only on the data, our approach and results can be extended to the case of realized discrepancies, where the realized discrepancy depends on both the data and the parameter. For example, in supplementary materials 1.4 we show that, for the Gaussian location model and mean-square error discrepancy $d(X,\theta )=\frac{1}{N}\sum_{i=1}^{N}{\left(X_{i}-\theta \right)}^{2}$, $Pr\left[p_{\text{SPC}}(X)<\alpha \right]\overset{P\;}{\to }1-\Phi \left(z_{1-\alpha }/\rho \right)$, where $\rho =\sigma_{\circ }^{2}/\sigma^{2}$. Hence, we reach similar conclusions about the q-SPC p-values for realized discrepancies being asymptotically well-calibrated but possibly having asymptotic power less than one.

### 3.2. Divided SPCs

**Figure 2.**
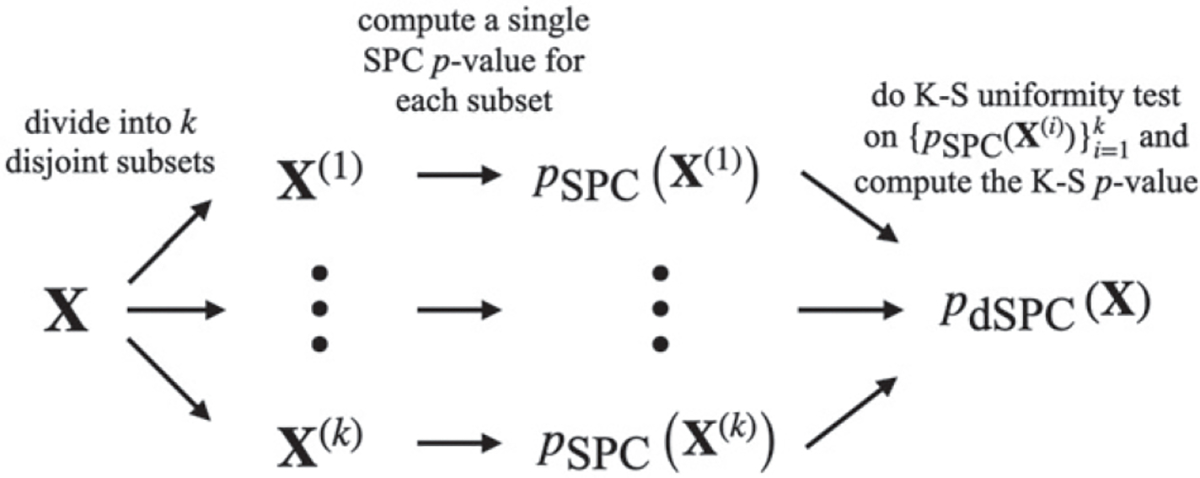
A schematic diagram of divided SPC. Divided SPC splits the original dataset into k disjoint subsets $X^{\left(i\right)},i=1,\ldots ,k$ and computes a single SPC p-value for each subset. The divided SPC p-value is the p-value from the Kolmogorov-Smirnov test on all single SPC p-values.

In light of the possibly poor sensitivity of the single SPC in the mild misspecification case, we aim to develop an alternative, more sensitive split predictive check that is guaranteed to have asymptotic power of one when the model is misspecified. We build upon the single SPC by observing that, when the model is misspecified, we still expect the asymptotic distribution of the single SPC p-values to be nonuniform. Hence, if we have many such p-values, we can instead test for their uniformity. More precisely, given data X, divide the original data into k equal folds of size $N_{k}\approx N/k$. Denoting these folds by $X^{\left(1\right)},\ldots ,X^{\left(k\right)}$, the next step is to compute the single q-SPC p-value $u_{kj}:=p_{\text{SPC}}(X^{\left(j\right)})$ for each fold j=1,…,k. To assimilate all the k single SPC p-values, we use the Kolmogorov-Smirnov (KS) test adapted to the case where the null distribution is uniform (Massey 1951). Specifically, we define the *divided SPC p-value* as

$$p_{dSPC}\left(X_{obs}\right)\;:=p_{KS}\left(u_{k1},\ldots ,u_{kk}\right),$$

where $p_{KS}(\cdot )$ denotes the p-value of the KS uniformity test. A schematic diagram of how to calculate the divided SPC p-value is presented in Figure 2.

Let Unif denote the uniform distribution on [0, 1] and $P_{k}$ be the distribution of a k single SPC p-value using $N_{k}$ observations. Let P^k denote the corresponding empirical distribution of k such p-values computed on independent datasets. For probability distributions μ and ω on R, define the Kolmogorov distance by $d_{K}(\mu ,\omega )\;:={sup}_{u\in R}\;|\mu ((-\infty ,u])-\omega ((-\infty ,u])|$. We require the following assumption to prove the correct asymptotic calibration of divided SPC *p*-values.

#### Assumption 1.

There exist constants C>0 and $\overset{‾}{\gamma }\in (0,\;1]$ such that for all $\gamma <\overset{‾}{\gamma }$, $d_{K}\left(P_{k}\right.,\;Unif)<CN_{k}^{-\gamma /2}$.

In Appendix A, Theorem 4 provides checkable conditions under which the assumption holds. In particular, under the hypotheses of Theorem 4, Assumption 1 holds with $\overset{‾}{\gamma }=s/(s+1)$, where s captures the model regularity. For sufficiently regular models, any s≥2 may be chosen, in which case $\overset{‾}{\gamma }$ can be arbitrarily close to 1.

#### Theorem 2.

If Assumption 1 olds and $k=\left\lfloor bN^{\beta }\right\rfloor$ for some b>0 and $0<\beta <\frac{\overset{‾}{\gamma }}{\overset{‾}{\gamma }+1}$, then for any t>0,

limN→∞∣Prk1/2dKP^k,Unif>t−1−FKS(t)∣=0,

where $F_{KS}(\cdot )$ is the CDF of the Kolmogorov distribution. Hence, ${lim}_{N\to \infty }\;d_{K}\left(p_{\text{dSPC}}\left(X_{\text{obs}}\right)\right.,\;Unif)=0$.

Theorem 2 suggests that k cannot grow too fast compared to N if we wish to control the test size. For example, if s can be chosen arbitrarily large, then we may take $k=O\left(N^{\beta }\right)$ for any β∈(0,1/2). On the other hand, if we only assume s≥2, then we must take β∈(0,2/5).

Having established the correct calibration of divided SPC *p*-values, our final result shows that, when the single SPC *p*-values are not asymptotically uniform, the asymptotic power of the divided SPC is one.

#### Theorem 3.

If ${lim\;inf}_{k\to \infty }\;d_{K}\left(P_{k}\right.,\;Unif)>0$, then for any t>0,

limk→∞PrkdKP^k,Unif>t=1.

We note that when the model is misspecified and ρ≠1, Theorem 2 shows that the distribution of SPC p-values is nonuniform, so the hypotheses of Theorem 3 hold.

## 4. Using Split Predictive Checks in Practice

We next provide some guidance on how to apply split predictive checks in practice, which we validate in our simulation studies in Section 5.

### 4.1. Using Single SPCs

As discussed earlier, most of the assumptions required by Theorem 1 are quite mild. The major assumption that the analyst has to be careful about is that the test statistic must be asymptotically normal.

#### Choosing q.

Theorem 1 shows that the split proportion q controls the relative weight placed on two forms of misspecification quantified by ρ: the uncertainty of the statistic T and the optimal parameter $\theta_{⋆}$. Choosing q large assigns more weight to the comparison between $\sigma_{\circ }$ and $\sigma \left(\theta_{⋆}\right)$ while a small q emphasizes the mismatch between frequentist sampling-based uncertainty $J_{⋆}^{-1}\Sigma_{⋆}J_{⋆}^{-1}$ and standard posterior uncertainty $J_{⋆}^{-1}$ in the direction $\dot{v}\left(\theta_{⋆}\right)$. A canonical choice for q is 0.5, which treats the calibration of the two types uncertainty as equally important.

In a small-sample regime, we recommend against extreme values for q. Small split proportions q may result in an inadequate amount of observed data, while choosing q large may result in poor estimation of the statistic. Hence, we recommend choosing a moderate value for q (e.g., 0.5 or 0.7) when the dataset size is fairly small relative to the model complexity.

#### Accounting for model structure.

There are many ways to split the data for structured models. The choice of splitting strategy should be guided by what type of generalization ability the analyst is interested in. The desired type of generalization also informs the choice of test statistic. To illustrate ideas, we discuss two canonical cases: hierarchical and time-series models.

We first consider the case of a two-level hierarchical structure:

$$\eta_{0}∼H$$

$$\eta_{i}\overset{\;\text{iid}\;}{∼}G_{\eta_{0}}\;(i=1,\ldots ,I),$$

$$X_{ij}\overset{\;\text{indep}\;}{∼}F_{\eta_{i}}\;\left(i=1,\ldots ,I;j=1,\ldots ,J_{i}\right).$$

**Figure 3.**
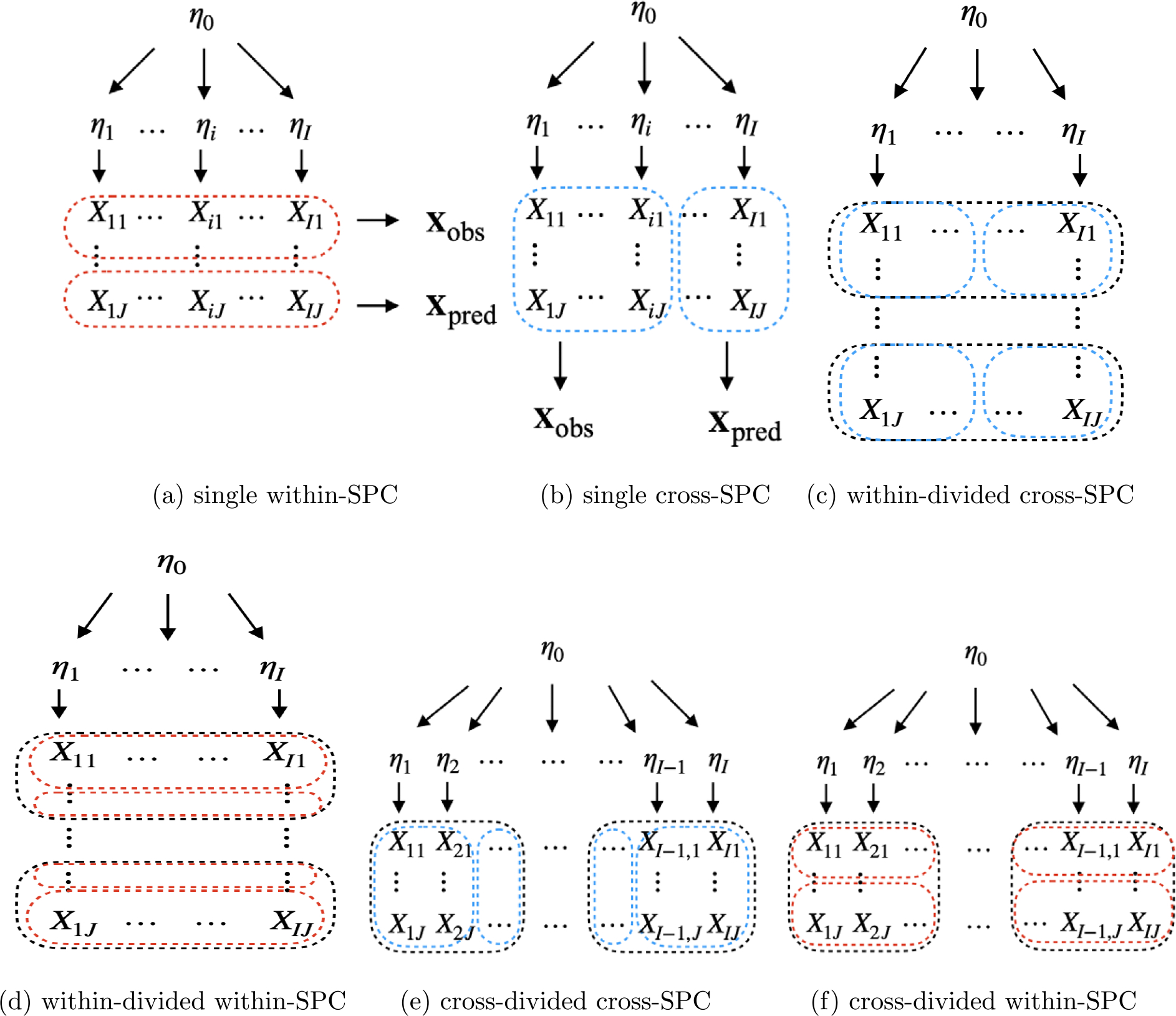
A schematic diagram of splitting strategies for single SPCs and divided SPCs in a two-level hierarchical model. Blue dotted circles refer to a single cross-SPC test for the subset. Red dotted lines indicate a single within-SPC *p*-value for each subset.

When applying the single SPC to such a model, we may be interested in *within-group* or *out-of-group* generalization. Within-group generalization (i.e., to new j with known i) requires splitting the data within each group, as shown in Figure 3(a). Out-of-group generalization (i.e., to new i) requires splitting the data across groups, as shown in Figure 3(b). We call the former *single within-SPC* and the latter *single cross-SPC*. Similar considerations can be applied to more complex hierarchical structures.

For a time-series model, the goal may be to make interpolative predictions (to times between those that are observed) or extrapolative predictions (to times beyond those that are observed). These goals lead to different splitting strategies. In the *interpolated single SPC*, for every m≪N observations, take the first ⌊qm⌋ as observed data and the reminder as held out. The value of m should be informed by how large the interpolative gaps are expected to be. The interpolated single SPC might also be appropriate for short-range extrapolation. But if interest is in longer-term extrapolation, then the *extrapolated single SPC*—taking the first ⌊qN⌋ data points as $X_{\text{obs}}$ and holding out the rest—would be more appropriate.

### 4.2. Using Divided SPCs

In addition to q, divided SPCs require choosing the number of splits k, while structured models also require additional considerations. We address each in turn.

#### Choosing q and k.

The choice of q requires the same considerations as in the single SPC case. However, we must judge the dataset size by $N_{k}$, the sample size used to compute the k single q-SPC p-values. The performance of divided SPCs could be significantly affected by the number of splits k. In general, a larger k will improve the power of the KS test but lead to $N_{k}$ being smaller, which may result in single q-SPC p-values being closer to uniform. We suggest using divided SPCs when N is very large, so choosing $k=\left\lfloor N^{\beta }\right\rfloor$ with β as large as possible is appropriate. Hence, based on Theorem 2 and the discussion that follows, for sufficiently regular models we suggest taking β just below 1/2 (e.g., 0.49). For less regular models, a safe choice is β just below 2/5 (e.g., 0.39).

#### Accounting for model structure.

Divided SPCs have two splitting steps: first divide the original data into k subsets, then apply single splits to each of the subset. For the hierarchical model (4.1), each split step can be across- or within-groups. Therefore, we have four possibilities, which we illustrate in Figure 2. For A,B∈{cross,within}, we refer to using the A split for the first step and the B split for the second step as the A*-divided* B-*SPC*. For example, the *cross-divided within-SPC* refers to doing cross-group splits to get subsets and single within-SPC for each subset. In general, we recommend taking A≠B because splitting the same way twice leads to very small sample sizes. The type of generalization of interest should determine B, since that is the split for the single SPC calculations. The application of divided SPCs to time-series data works in a similar way to hierarchical models: for each level of splitting in divided SPCs, there are two choices—extrapolation and interpolation—with the analyst’s goals dictating the best combination of splitting strategies.

## 5. Simulation Studies

To validate our theory from Section 3 and guidelines from Section 4, and investigate the finite-sample effects of SPC tuning parameters (the split proportion q and, for the divided SPC, the number of folds k), we carry out two simulation studies, with a conjugate Poisson model (in the supplementary materials) and a Gaussian hierarchical model. Specically, we consider the two-level Gaussian hierarchical model from Bayarri and Castellanos (2007), which corresponds to the model in (4.1) with $F_{\eta_{i}}=𝒩\left(\eta_{i},4\right)$, $G_{\eta_{0}}=𝒩\left(\mu_{0},\sigma_{0}^{2}\right)$, and an improper prior H on $\eta_{0}=(\mu_{0},\sigma_{0}^{2})$ with density $h\left(\mu_{0},\sigma_{0}^{2}\right)=\sigma_{0}^{-2}$. To illustrate how SPCs compare to posterior predictive checks under different forms of model—data mismatch, we simulate data based on the four scenarios described Table 1.

### Statistics for hierarchical model.

**Figure 4.**
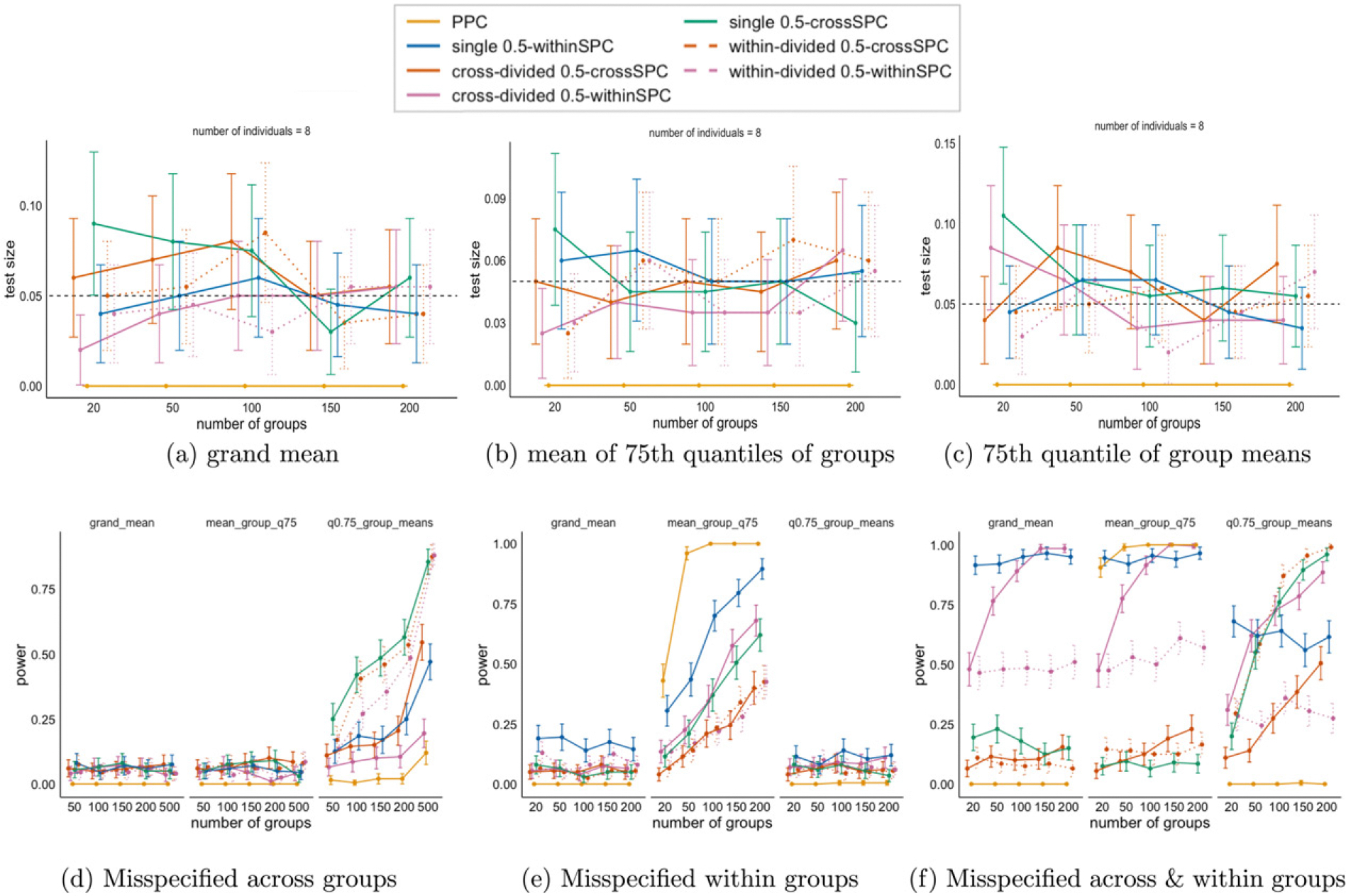
Test size (a,b,c) and power (d,e,f) plots for different checks and statistics given fixed number of individuals J=8 and increasing number of groups I∈{20,50,100,150,200} under Gaussian hierarchical model. The data generating distributions for each scenario are listed in Table 1. Vertical bars represent the 95th confidence intervals for the estimation. The horizontal dashed line indicates significance level α=0.05. Dotted lines are divided SPC using within splitting strategy to get k folds as discussed in Section 4.

Let Q(x) denote the 75th quantile of data x and ${\overset{‾}{x}}_{i}$ be the mean of the ith group of observations. We employ three statistics in our simulations: (i) the grand mean $T_{IJ}(x)=\frac{1}{IJ}\sum_{i=1}^{I}\sum_{j=1}^{J}x_{ij}$, (ii) the mean of the 75th quantiles of each group $T_{IJ}(x)=\frac{1}{I}\sum_{i=1}^{I}Q\left(x_{i.}\right)$, and (iii) the 75th quantile of the group means $T_{IJ}(x)=Q\left({\overset{‾}{x}}_{1},\ldots ,{\overset{‾}{x}}_{I}\right)$. For each candidate check, we estimate the test size by repeatedly generating data from well-specified model for 200 times and run each candidate check in each iteration to get their corresponding *p*-values. Powers are estimated with 200 repeated experiments for each scenario. Figure 4 shows results for a fixed number of observations J=8 per group and an increasing number of groups I. Results and discussions for fixed I=20 with increasing J are presented in supplementary materials 2.3.

### Calibration.

We first verify our theory concerning the calibration of SPCs. Figure 3(a)–(c) show that in the well-specified Scenario 1 for all statistics both single and divided SPCs correctly control test size and produce uniform *p*-values (see also Fig. B.18f in supplementary materials). PPCs have the smallest test size but the *p*-values are not uniformly distributed (see also Fig. B.18e in supplementary materials) and so are not well-calibrated. As discussed in Section 4, a hierarchical structure may produce different levels of misspecification for which only certain test statistics and checking methods will be effective.

### Misspecified across groups scenario.

Since in this scenario the misspecification is at the group level, the 75th quantile of the group means statistic is most sensitive. Figure 4(d) shows that, both single and divided SPCs dramatically outperform PPCs in detecting an issue with predictive generalization to new groups. Also, as suggested in Section 4, single cross-SPCs are best for testing for group-level misspecification and cross-divided cross-SPCs have smaller power than within-divided cross-SPCs because doing cross-splits twice results in uninformative posteriors in a small data regime.

### Misspecified within groups scenario.

In this scenario the observation-level mismatch between the model and data leads to the model not generalizing to new observations within a group and will also lack self-consistency. Here, the mean of the group 75th quantiles statistic is most effective at detecting the misspecification. Figure 4(e) shows that, as expected, PPCs and SPCs are both effective, although PPCs have greater sensitivity. Moreover, as discussed in Section 4, the within-SPC methods are more effective than cross-SPC methods.

### Misspecified across and within groups scenario.

Since in this scenario there is misspecification at both levels, all three statistics are capable of detecting model—data mismatch. However, Figure 4(f) shows that PPCs are only effective when used with the mean of group 75th quantiles statistic while SPCs are effective for all three statistics. These results are consistent with the previous two scenarios. For the mean of group 75th quantiles statistic (better for detecting the lower-level misspecification), and the grand mean, only the within-SPCs have good sensitivity; the cross-divided within-SPC outperforms the within-divided within-SPC due to the small data effects. The grand mean statistic results are similar. For 75th quantile of group means, which captures the group-level misspecification, cross-SPCs outperform within-SPCs, as expected.

### Summary.

Overall, the simulation study confirms our theoretical findings and methodological guidance. PPCs can detect low-level model misspecification that leads to inferences that aren’t self-consistent. On the other hand, SPCs are more flexible, and are the only method that can detect issues with generalization to new groups.

## 6. Illustrations

In this section, we confirm the findings of our simulation study by comparing SPCs to various predictive checks using two real-data illustrations.

### 6.1. Microarray Prediction Problem

Predictive checking methods that target predictive accuracy versus self-consistency can yield substantially different conclusions (Efron 2020). SPCs can evaluate both prediction performance and self-consistency, whereas standard predictive checks typically focus only on self-consistency and may have reduced power for assessing predictive ability. To illustrate this distinction, we analyze a publicly available microarray dataset from Singh et al. (2002), which contains gene expression measurements for *p* = 6033 genes across *n* = 102 samples consisting of data from 50 healthy controls and 52 cancer patients. We define a binary response y∈{0,1} indicating whether an individual is a cancer patient or a healthy control. Because these data are extremely high-dimensional (p≫n) and are believed to exhibit substantial sparsity, we keep the top 500 top genes by variance and model them using a logistic regression model with a horseshoe prior on the regression coefficients (Carvalho, Polson, and Scott 2009):

$$\begin{array}{l} y_{i}|\alpha ,\beta ∼Bern\left(\pi_{i}\right),\;\pi_{i}={logit}^{-1}\left(\alpha +x_{i}^{⊤}\beta \right),\\ \beta_{j}|\lambda_{j}\tau ∼𝒩\left(0,\lambda_{j}^{2}\tau^{2}\right), \end{array}$$

where $\lambda_{j}∼{𝒞}^{+}(0,\;1)$ is a local shrinkage parameter and $\tau ∼{𝒞}^{+}(0,\;1)$ is the global shrinkage parameter. We also set weakly informative priors for the intercept and regression coefficients: α∼𝒩(0,5) and $\beta_{j}∼𝒩(0,\;1)$.

To construct SPCs for both self-consistency and predictive accuracy, following Efron (2020), we first created training and test sets by randomly splitting the 102 samples into two groups of 51, each containing 25 healthy controls and 26 cancer patients. This split was used to assess self-consistency, that is, how well the model fits the training data. For SPCs targeting predictive performance, the training set was selected to include the 25 healthy controls and 26 cancer patients with the lowest ID numbers, while the test set consisted of the remaining 51 samples with the highest IDs, again maintaining 25 healthy controls and 26 cancer patients.^1^

We evaluated the model trained on the low-ID subset by computing its prediction accuracy on the test data, which resulted in an error rate of 59.8%, indicating poor generalization. We compared SPCs to standard PPCs, prior predictive checks, and the calibrated post-processing PPC proposed by Hjort, Dahl, and Steinbakk (2006). As shown in Table 2, SPCs that are specifically designed to target predictive performance successfully detect the model’s limited predictive ability, whereas the PPC and calibrated PPC (Hjort) checks fail to do so. The prior predictive check yields a small *p*-value, but this is not meaningful: weak priors cause the predictive distribution to differ from the observed data without implying a poorly behaved model. On the other hand, when assessing how well the model fits the training data, the estimated error rate is only 0.01%, indicating excellent self-consistency. SPCs targeting self-consistency correctly do not reject the model in this case. Overall, SPCs provide a flexible framework that can distinguish between predictive performance and self-consistency depending on the way the observations are split.

### 6.2. Airline Delays Data

We conclude by illustrating the flexibility of SPCs for assessing different forms of predictive generalization. We use the airline delays dataset containing the on-time performance records for all flights departing New York City in 2013.^2^ The dataset includes flight information such as departure and arrival times, distance, carrier, and the origin and destination airports. Each observation corresponds to a single flight, and the response variable is the arrival delay (in minutes). We consider two predictive scenarios: time generalization and destination generalization.

#### 6.2.1. Time Generalization

For the time generalization scenario, we fit a linear regression model that includes month-specific covariates. Let $y_{i}$ denote the arrival delay (in minutes) for flight i=1,...,N. Let ${dep\_delay}_{i}$ denote departure delay, ${\;distance}_{i}$ denote the flight distance, and $m_{i}\;\in \;\{1,\;...,12\}$ denote the month in which the flight occurred. The model predicts arrival delay using departure delay, flight distance, and smooth seasonal components based on the month of the year:

(3) $$\begin{array}{l} y_{i}|\mu_{i},\sigma ∼𝒩\left(\mu_{i},\sigma^{2}\right),\\ \mu_{i}=\alpha +\beta_{\text{dep}}\cdot {\text{dep\_delay}}_{i}+\beta_{\text{dist}}\cdot {\text{distance}}_{i},\\ \;+\beta_{c}\cdot c_{i}+\beta_{s}\cdot s_{i}, \end{array}$$

where $c_{i}=cos\left(m_{i}/12\pi \right)$ and $s_{i}=sin\left(m_{i}/12\pi \right)$ are the seasonal covariates. We use weakly informative priors on all regression parameters: $\beta_{j}∼𝒩(0,\;1)$ and α∼𝒩(0,5).

**Figure 5.**
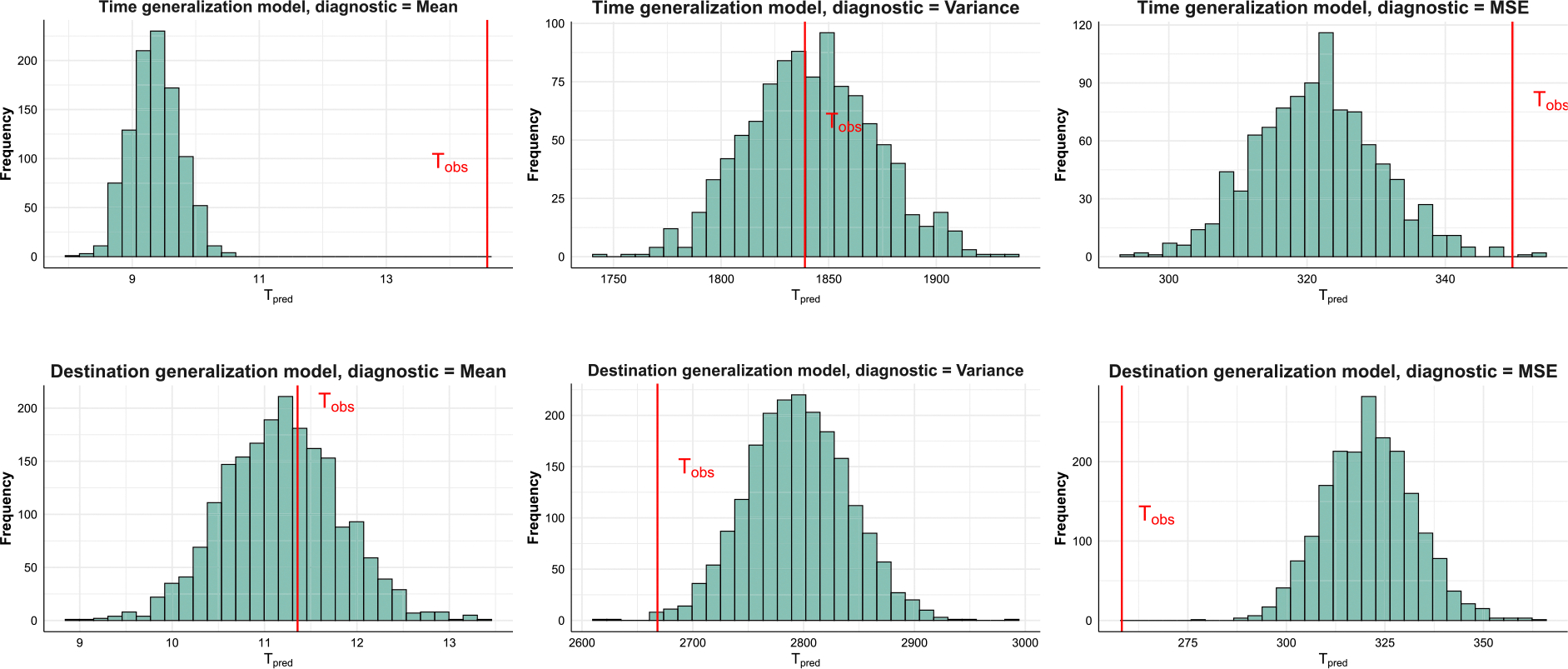
Histograms of diagnostic functions based on posterior predictive samples $y_{\text{pred}}$ for the time generalization model (top row) and destination generalization model (bottom row) in the airline delay experiments. The posterior samples correspond to predictions on the hold-out dataset: $y_{\text{pred}}∼p\left(y|\theta ,X_{\text{ho}}\right)p\left(\theta |X_{\text{obs}},y_{\text{obs}}\right)$. The vertical red line indicates the value of the diagnostic function computed on the observed training data. These graphical checks provide more detailed information about the model’s prediction performance. For example, in the case of the MSE diagnostic, for time generalization it is underestimated while for destination generalization it is more severely overestimated.

For SPCs, we define the observed dataset $X_{\text{obs}}$ as all data from January through November and the hold-out dataset $X_{\text{ho}}$ as all observations from December. We use three different discrepancy measures to evaluate model generalization: (a) empirical mean $T(y)=\frac{1}{N}\sum_{i=1}^{N}y_{i}$, (b) empirical variance: $T(y)=\frac{1}{N}\sum_{i=1}^{N}{\left(y_{i}-\overset{‾}{y}\right)}^{2}$, and (c) mean squared error (MSE) $d(y,\theta )=\frac{1}{N}\sum_{i=1}^{N}{\left(y_{i}-{\overset{‾}{y}}_{i}^{\text{pred}}\right)}^{2}$, where ${\overset{‾}{y}}_{i}^{\text{pred}}=E[y_{i}^{\text{pred}}|y]=\frac{1}{S}\sum_{s=1}^{S}y_{i,s}^{\text{pred}}$. For this model on the hold-out dataset, RMSE ≈ 61, which indicates that the predicted arrival delay differs from the observed arrival delay by about 61 min on average. As shown in Table 3, only the SPC rejects the model for the mean and MSE diagnostic while the prior predictive check (Prior PC), PPC, and calibrated PPC suggested by Hjort, Dahl, and Steinbakk (2006) all fail to reject for all diagnostic functions. SPC does not reject when applying a variance diagnostic function. Figure 5 illustrates the checks graphically.

#### 6.2.2. Destination Generalization

We conclude by comparing the predictive checks on a similar model that aims to generalize predictions to new destinations. We use a simple hierarchical Gaussian regression model with destination-specific intercepts:

(4) $$\begin{array}{l} y_{i}|\mu_{i},\sigma ∼𝒩\left(\mu_{i},\sigma^{2}\right),\\ \mu_{i}=\alpha +\beta_{\text{dep}}\cdot {\text{dep\_delay}}_{i}+\beta_{\text{dist}}\cdot {\text{distance}}_{i}+\beta_{\text{dest}[i]} \end{array}$$

where $\beta_{\text{dest}[i]}∼{𝒯}_{3}(0,\;10)$ is a heavy-tailed Student-t prior on the random intercepts, allowing some destinations to have extreme effects, and the fixed effects and intercept have broad priors: $\alpha ,\beta_{j}∼𝒩(0,\;10)$. This specification is deliberately weakly regularized to allow overfitting to the training data while still producing meaningful predictions for new destinations.

We apply the three diagnostic functions described above to compare different model checking methods for generalization to unseen destinations. As shown in Table 3, SPC rejects the model when using the variance and MSE diagnostics, but not for the mean statistic. In contrast, all other methods fail to reject any of the diagnostics. These results are supported by both RMSE calculations on the test data and graphical checks: the RMSE for this model on unseen destinations is approximately 73.89 min, indicating poor generalization. As illustrated in Figure 5, for both variance and MSE, the observed diagnostic values lie far from the distributions obtained from posterior predictive samples, whereas the posterior predictive draws capture the mean arrival delays accurately, consistent with the SPC results.

## 7. Conclusion

Split predictive checks (SPCs) provide a simple, general-purpose framework for assessing a Bayesian model’s ability to predict unobserved data. By explicitly separating the data used for fitting from the data used for checking, SPCs avoid the self-consistency bias inherent in posterior predictive checks and focus the assessment on predictive generalization. Our theoretical results and empirical studies show that SPCs retain the ease and scalability of PPCs while offering much stronger guarantees about detecting model—data mismatches that matter for prediction.

### Power and degree of misspecification.

Our results reveal a clear hierarchy in the sensitivity of predictive checks that aligns with distinct modes of model failure. When the model fails to match the expected value of the test statistic under the data-generating process (what we refer to as *major misspecification*), both single and divided SPCs achieve asymptotic power one, as they should. When the test statistic means agree but the model underestimates the uncertainty of the statistic or parameters (*moderate misspecification* corresponding to ρ≫1 in our theory), single SPCs remain highly effective. They can detect the resulting predictive overconfidence that PPCs may miss. Finally, when the model’s predictive uncertainty is only mildly inaccurate (*mild misspecification*), divided SPCs retain power by aggregating many nearly-uniform single-SPC *p*-values; PPCs and single SPCs, on the other hand, may show little sensitivity. In short, PPCs excel when detecting extreme failures of model–data consistency, single SPCs detect systematic underestimation of predictive uncertainty, and divided SPCs detect even subtle deviations in predictive calibration.

### Dataset size and computational efficiency.

All three methods require similar computational effort. PPCs use only the already computed posterior. Single SPCs require obtaining the posterior of the observed split of the data, which will typically require about half the computational effort of the original posterior calculation since we recommend choosing q≈0.5. While divided SPCs require one posterior computation for each subset, the dataset sizes are small. So, the total cost should be roughly equal to that of computing the original posterior. As for different dataset sizes, divided SPCs require a moderate-to-large dataset since when the dataset is small, there is an insufficient amount of data in each subset of size $N_{k}$. On the other hand, both PPCs and single SPCs remain effective in the small-data regime.

### Choosing the right predictive check.

The appropriate check depends on the scientific goal and the type of generalization required.

- Posterior predictive checks (PPCs) remain useful when the primary concern is explanatory adequacy—that is, whether the model reproduces features already present in the data. They are less appropriate when assessing predictive generalization.
- Single SPCs are a good choice when moderate-to-substantial misspecification is of practical concern: they directly test out-of-sample predictive behavior, are computationally cheap, and work well even in small datasets when the fitted model’s uncertainty quantification is inadequate.
- Divided SPCs should be used when sensitivity is paramount or when subtle model failures may materially affect predictions. They require larger datasets but offer the strongest detection guarantees, capturing even mild predictive miscalibration.

For structured models, the choice of SPC splitting strategy should reflect the form of generalization required (e.g., across groups vs. within groups, interpolation vs. extrapolation, short- vs. long-range prediction).

### Comparison to held-out predictive checks.

In independent and concurrent work, Moran, Blei, and Ranganath (2024) similarly identify shortcomings with existing predictive checks. In a concurrent revision, they propose the *held-out predictive check*, which is the same as the single SPC. They show that it produces asymptotically uniform *p*-values when the model is correctly specified (Moran, Blei, and Ranganath 2024, Theorem 1), which is a special case of our Theorem 1(1) when the model is well-specified. Moran, Blei, and Ranganath (2024) also demonstrate empirically that post-hoc calibration approaches for PPCs do not resolve the “double use of the data” problem, providing a complementary perspective to ours.

### Future work.

There are several interesting directions for future research. Our simulation study and theory for the normal location model suggest the calibration properties of SPCs extend to the case of realized discrepancies, but it would be worthwhile to develop a more comprehensive theory. Such a theory could also cover discrepancies that have nonnormal limiting distributions.

## Supplementary Material

Supp 1

Supp 2

This supplementary document provides additional methodological and empirical details. Section S1 includes lemmas, assumptions, and detailed proofs for the theorems in Section 3. Section S2 provides additional figures and tables for the simulation study and experiments. All relevant code and reproducible materials are available at the project’s GitHub repository: https://github.com/TARPS-group/split-predictive-checks/tree/main.

Supplementary materials for this article are available online. Please go to www.tandfonline.com/r/JASA.

## Figures and Tables

**Table 1.** Four scenarios with four types of misspecification in a hierarchical setup.

Scenario name	$X_{ij}$ Distribution	$\eta_{i}$ Distribution

Well-specified	$X_{ij}|\eta_{i}∼𝒩\left(\eta_{i},4\right)$	$\eta_{i}∼𝒩(0,1)$
Misspecified across groups	$X_{ij}|\eta_{i}∼𝒩\left(\eta_{i},4\right)$	$\eta_{i}∼Gamma(0.6,0.2)$
Misspecified within groups	$X_{ij}|\eta_{i}∼𝒩\left(\eta_{i},8\right)$	$\eta_{i}∼𝒩(0,1)$
Misspecified across and within groups	$lnX_{ij}|\eta_{i}∼𝒩\left(\eta_{i},4\right)$	$\eta_{i}∼𝒩(0,1)$

**Table 2.** Comparison of methods for microarray data under a horseshoe prior with global shrinkage parameter hyperprior $\tau ∼{𝒞}^{+}(0,\;1)$.

Method	*p*-value

PPC	0.350
PPC (Hjort)	0.978
Prior PC	0.000
SPC (consistency)	0.759
SPC (prediction)	0.020

**Table 3.** *p*-values for different diagnostic functions across various checking methods and scenarios.

Scenario	Method	Mean	Variance	MSE

Time generalization (Section 6.2.1)	PPC	0.969	0.944	0.892
	PPC (Hjort)	0.999	0.999	0.999
	Prior PC	0.965	0.974	0.874
	SPC	0.000	0.970	0.002

Destination generalization (Section 6.2.2)	PPC	0.984	0.972	0.809
	PPC (Hjort)	0.999	0.999	0.999
	Prior PC	0.984	0.982	0.985
	SPC	0.769	0.005	0.000

## Footnotes

1 As pointed out in Efron (2020), the prostate study subjects were likely collected sequentially according to their listed order, potentially exposing shifts in data collection or the patient population as the study progressed.

2 https://www.transtats.bts.gov/DL_SelectFields.asp?Table_ID=236 After removing entries with missing values and randomly sampling 10% of the remaining data, we obtain a cleaned dataset containing *N* = 32,735 flights.

## Appendix A: Assumptions and Additional Results

### A.1. Assumptions

First, we require some regularity conditions on the test statistic under the assumed model. Recall that $\theta_{⋆}:={sup}_{\theta \in \Theta }\;E\left\{log\;p_{\theta }\left(X_{1}\right)\right\}$ is the Kullback–Leibler-minimizing parameter.

#### Assumption 2.

Assume that

- The asymptotic mean $v(\theta )\;:={lim}_{N\to \infty }\int T_{N}(x)P_{\theta }(dx)$ and asymptotic variance $\sigma^{2}(\theta )\;:={lim}_{N\to \infty }\int T_{N}^{2}(x)P_{\theta }(dx)-v^{2}(\theta )$ exist;
- the asymptotic mean is twice-differentiable and finite at $\theta_{⋆}$, so $\dot{v}\left(\theta_{⋆}\right)\;:={\left.\underset{N\to \infty }{lim}\;\frac{\partial v(\theta )}{\partial \theta }\right|}_{\theta =\theta_{⋆}}$ and $\ddot{v}\left(\theta_{⋆}\right)\;:={\left.\underset{N\to \infty }{lim}\;\frac{\partial \dot{v}(\theta )}{\partial \theta }\right|}_{\theta =\theta_{⋆}}$ are well-defined and ${\left\|\ddot{v}\left(\theta_{⋆}\right)\right\|}_{2}<\infty$; and
- there exists an open neighborhood $U_{⋆,1}∋\theta_{⋆}$ and constant M>0 such that for all $\theta \in U_{⋆,1}$, $\dot{\sigma }(\theta )\;:=\frac{\partial \sigma (\theta )}{\partial \theta }$ exists and $\|\dot{\sigma }(\theta ){\|}_{2}\leq M$.

Our second assumption requires the scaled test statistic to satisfy a central limit theorem, which will typically be the case (e.g., when T takes the form of an average or is a U-statistic).

#### Assumption 3.

There exists an open neighborhood $U_{⋆,2}∋\theta_{⋆}$ such that for $\theta \in U_{⋆,2}$ and $Y_{1},\ldots ,Y_{N}\overset{\text{iid}}{∼}P_{\theta }$,

(A1)
$$\frac{N^{1/2}\left\{T_{N}\left(Y_{N}\right)-v(\theta )\right\}}{\sigma (\theta )}\overset{𝒟\;}{\to }𝒩(0,\;1).$$

Our third assumption requires asymptotic normality of the maximum likelihood estimator $\overset{ˆ}{\theta }(X)\;:=arg\;\underset{\theta \in \Theta }{max}\;p_{\theta }(X)$ and the posterior. Denote the usual information matrices by $J_{⋆}:=-E_{P_{o}}\left[\nabla^{2}log\;p_{\theta_{⋆}}\left(X_{1}\right)\right]$ and $\Sigma_{⋆}:={cov}_{P_{o}}\left[\nabla log\;p_{\theta_{⋆}}\left(X_{1}\right)\right]$, and denote the total variation distance between distributions μ and ω by $d_{TV}(\mu ,\omega )\;:=2\;sup\{|\mu (B)-\omega (B)|\;:\;measurable\;B\subseteq \Theta \}$.

#### Assumption 4.

Assume that

- $N^{1/2}\{\overset{ˆ}{\theta }\left(X_{N}\right)-\theta_{⋆}\}\overset{𝒟\;}{\to }𝒩{\left(0,J_{⋆}^{-1}\Sigma_{⋆}\right)}_{⋆}^{-1})$ and
- $d_{TV}(\Pi (\cdot |X_{N}),𝒩(\overset{ˆ}{\theta }\left(X_{N}\right),J_{⋆}^{-1}/N))\overset{P\;}{\to }0$.

The conditions for Assumption (A4-a) to hold are standard while Kleijn and van der Vaart (2012) gives general conditions under which Assumption (A4-b) holds.

### A.2. Additional Results

In this section, we provide sufficient conditions for Assumption 1 to hold. We recall that for distributions μ and ω on R, the Kolmogorov distance is given by $d_{K}(\mu ,\omega )\;:={sup}_{u\in R}\;|\mu ((-\infty ,u])-\omega ((-\infty ,u])|$. We sometimes slightly abuse notation and write the distance $d_{K}(U,V)$ instead of $d_{K}(\mu ,\omega )$ for random variables U and V having distributions μ and ω. In addition to Assumption 2, the assumptions required are versions of Assumptions 3 and 4 with quantitative bounds on the convergence rate. Specifically, we require high-probability bounds on the error of the maximum likelihood estimator $\overset{ˆ}{\theta }(X)\;:=arg\;{max}_{\theta \in \Theta }\;p_{\theta }(X)$ and the normality of the posterior. For a set $S\subseteq R^{d}$, define the constrained total variation distance $d_{TV,S}(\mu ,\omega )\;:={sup}_{B\subseteq ℬ(S)}\;|\mu (B)-\omega (B)|$, where ℬ(S) is the collection of all Borel subsets of S.

#### Assumption 5.

There exists a constant s≥2 such that, for any compact set 𝒦 satisfying $\theta_{⋆}\in Int(𝒦)$, there exist constants $c_{𝒦},c_{𝒦}^{\prime }>0$ such that

- $Pr[\left\|\overset{ˆ}{\theta }\left(X_{N}\right)-\theta_{⋆}\right\|>c_{𝒦}N^{-1/2}]=O(N^{-s/2})$ and
- $\begin{array}{l} Pr[d_{TV,𝒦}(\Pi (\cdot |X_{N}),𝒩(\overset{ˆ}{\theta }(X_{N}),\Sigma_{⋆}/N))>c_{𝒦}^{\prime }N^{-1/2}]\\ \;=O(N^{-s/2}). \end{array}$

Assumption (A5-a) hold under the regularity conditions listed in Hipp and Michel (1976, sec. 4). These conditions essentially impose some restrictions on the smoothness of the model density and the existence of lower-order moments of the log-likelihood, as characterized by the regularity parameter s≥2. We postpone the statement and discussion of the other conditions (Assumptions A6 and A7), which are stronger versions of Assumptions 3 and 4, to supplementary materials 1.3.

#### Theorem 4.

Suppose $P_{\circ }=P_{\theta_{⋆}}$. If Assumptions 2 and 5, A6 and A7 hold, then there exists an absolute constant C>0 such that

$$d_{K}\left(p_{SPC}(X),U\right)<Clog\frac{s}{1+s}N/N^{\frac{s}{2(1+s)}}.$$

The proof of Theorem 4 is in supplementary materials 1.3. It also follows from the proof of Theorem 4 that we could also use a Wasserstein distance-based uniformity test. However, in preliminary experiments we found the KS approach to be superior.
