# Supplementary material for: Calibrated Model Criticism Using Split Predictive Checks
Source: J Am Stat Assoc. Author manuscript; Available in PMC 2026 Aug 6. (PMC13441283; doi:10.1080/01621459.2026.2649585)
Supplement: Supplementary file 1 [file NIHMS2189864-supplement-Supp_1.pdf]

This form documents the artifacts associated with the article (i.e., the data and code supporting the computational findings) and describes how to reproduce the findings.

Part 1: Data

- ☐ This paper does not involve analysis of external data (i.e., no data are used or the only data are generated by the authors via simulation in their code).
- ☒ I certify that the author(s) of the manuscript have legitimate access to and permission to use the data used in this manuscript.

Abstract

We validate the proposed method using two publicly available datasets: the microarray gene-expression data of Singh et al. (2002), accessed via the singh2002 dataset in the sda R package, and the airline delays data provided through nycflights13 R package. The microarray dataset contains gene expression measurements for 50 healthy controls and 52 prostate cancer patients. The airline dataset provides on-time performance records for all flights departing New York City in 2013, originally sourced from the U.S. Department of Transportation and cached locally by nycflights13.

Availability

- ☒ Data **are** publicly available.
- ☐ Data **cannot be made** publicly available.

If the data are publicly available, see the *Publicly available data* section. Otherwise, see the *Non-publicly available data* section, below.

Publicly available data

- ☒ Data are available online at:
  - **Microarray gene-expression data (Singh et al., 2002):**
    Available through the **sda** R package.
    Load in R using:

    ```
    install.packages("sda")
    library(sda)
    data(singh2002)
    ```

  - **Airline delay data:**
    Available through the **nycflights13** R package. Load in R using:

    ```
    install.packages("nycflights13")
    library(nycflights13)
    flights
    ```

- ☐ Data are available as part of the paper's supplementary material.
- ☐ Data are publicly available by request, following the process described here:
- ☐ Data are or will be made available through some other mechanism, described here:

Non-publicly available data

Description

File format(s)

- ☐ CSV or other plain text.
- ☒ Software-specific binary format (.Rda, Python pickle, etc.): R `.rda` objects included in the **sda** and **nycflights13** packages.
- ☐ Standardized binary format (e.g., netCDF, HDF5, etc.):
- ☐ Other (please specify):

Data dictionary

- ☐ Provided by authors in the following file(s):
- ☐ Data file(s) is(are) self-describing (e.g., netCDF files)
- ☒ Available at the following URL:
  - **Microarray data (Singh et al., 2002):** Description available at https://search.r-project.org/CRAN/refmans/sda/html/singh2002.html.
  - **Airline delay data:** Description available at https://cran.r-project.org/web/packages/nycflights13/index.html.

Additional Information (optional)

Part 2: Code

Abstract

Description

Code format(s)

- ☒ Script files
  - ☒ R
  - ☐ Python
  - ☐ Matlab
  - ☐ Other:
- ☒ Package
  - ☒ R
  - ☐ Python
  - ☐ MATLAB toolbox
  - ☐ Other:
- ☒ Reproducible report
  - ☒ R Markdown
  - ☐ Jupyter notebook
  - ☐ Other:
- ☐ Shell script
- ☐ Other (please specify):

Supporting software requirements

Version of primary software used R version 4.0.3

Libraries and dependencies used by the code

- posterior version 1.1.0
- bayesplot version 1.8.1

- tibble version 3.0.4
- dplyr version 1.0.2
- tidyr version 1.1.2
- matrixStats version 0.57.0
- rstan version 2.21.2
- purrr version 0.3.4
- patchwork version 1.1.0
- sda version 1.3.9
- ggplot2 version 3.3.5
- brms version 2.16.3
- nycflights13 version 1.0.2
- MASS version 7.3-53
- tidyverse version 1.3.0
- BayesDA version 2012.4.1
- gtools version 3.8.2
- ggthemes version 4.2.4
- scales version 1.1.1
- RColorBrewer version 1.1-2
- stats version 4.0.3
- loo version 2.4.1
- twosamples version 1.1.1

Supporting system/hardware requirements (optional)

Parallelization used

☐ No parallel code used
☒ Multi-core parallelization on a single machine/node
  - Number of cores used: 4
☐ Multi-machine/multi-node parallelization
  - Number of nodes and cores used:

License

☒ MIT License (default)
☐ BSD
☐ GPL v3.0
☐ Creative Commons
☐ Other: (please specify)

Additional information (optional)

Part 3: Reproducibility workflow

Scope

The provided workflow reproduces:

☐ Any numbers provided in text in the paper
☒ The computational method(s) presented in the paper (i.e., code is provided that implements the method(s))
☐ All tables and figures in the paper
☒ Selected tables and figures in the paper, as explained and justified below:

All simulation studies and real-data experiment results can be reproduced using the provided code. Figures 2 and 3 are conceptual diagrams illustrating how the proposed method is defined and computed and are

therefore not generated by the code. Table 1 summarizes the experimental setup and is likewise not produced by the workflow.

Workflow

Location

The workflow is available:

☒ As part of the paper's supplementary material.
☒ In this Git repository: https://github.com/TARPS-group/split-predictive-checks
☐ Other (please specify):

Format(s)

☐ Single master code file
☐ Wrapper (shell) script(s)
☒ Self-contained R Markdown file, Jupyter notebook, or other literate programming approach
☐ Text file (e.g., a readme-style file) that documents workflow
☐ Makefile
☐ Other (more detail in *Instructions* below)

Instructions

The provided codebase is organized into two main components.

1. Core method implementation (`/spc` folder)
- `compute_spcs.R` and `compute_spcs_for_hierarchical.R` implement the SPCs method.
- `generate_pvals.R` contains functions for generating p-values for the simulation studies.
- `evaluation.R` provides visualization functions.
- `util.R` includes discrepancy functions used in the simulation study.

2. Reproduction scripts (`/script` folder)
To reproduce the illustrative figures in Fig. 1: Run `/simulation-models/illustrative_figures.Rmd`.

To reproduce results for simulation study in Section 5 and Appendix 2:

- The folder `/simulation-models` contains all the models used in the simulation study.
- To reproduce all simulation figures and tables, first run `simulation_main.R`, then knit `plot_simulation_results.Rmd`.

To reproduce results for real-data experiments in Section 6:

- Section 6.1 (Microarray data): run `experiments/microarray/microarray_main.Rmd`

- Section 6.2 (Airline delays data): run `experiments/airlines/airline_main.Rmd`

Expected run-time

Approximate time needed to reproduce the analyses on a standard desktop machine:

☐ < 1 minute
☐ 1-10 minutes
☐ 10-60 minutes
☐ 1-8 hours
☒ > 8 hours
☐ Not feasible to run on a desktop machine, as described here:

Additional information (optional)

Notes (optional)
