# Supplementary material for: Calibrated Model Criticism Using Split Predictive Checks
Source: J Am Stat Assoc. Author manuscript; Available in PMC 2026 Aug 6. (PMC13441283; doi:10.1080/01621459.2026.2649585)
Supplement: Supplementary file 2 [file NIHMS2189864-supplement-Supp_2.pdf]

Supplementary material for "Calibrated Model Criticism Using Split Predictive Checks"

Jiawei Li
Department of Mathematics & Statistics
Boston University
and
Jonathan H. Huggins
Department of Mathematics & Statistics and
Faculty of Computing & Data Sciences
Boston University

1 Proofs

1.1 Technical lemmas

We state some preliminary definitions and lemmas needed for the proofs of the single SPC-related results Theorems 3.1 and A.1.

To quantify the distance between probability measures, we rely on a number of different probability metrics: Kolmogorov distance, Wasserstein distance, total variation (TV) distance, and the bounded Lipschitz distance. It follows from the definitions $d_{TV,S} \leq d_{TV}$ and $d_K \leq d_{TV}$. The s-Wasserstein distance between distributions μ and ν defined on $\Omega \subseteq \mathbb{R}^d$ is given by $W_s(\mu,\nu) := \inf_\xi\{\int |u-v|^s \xi(\mathrm{d}u,\mathrm{d}v)\}^{1/s}$, where the infimum is over all distributions on $\Omega \times \Omega$ with marginals μ and ν; that is, with $\xi(\cdot,\mathbb{R}) = \mu$ and $\xi(\mathbb{R},\cdot) = \omega$ (Villani 2009). By Gibbs & Su (2002, Theorem 4.1), we have

$$W_s(\mu,\nu) \leq \mathrm{diam}(\Omega)\, d_{TV}(\mu,\nu).$$

where $\mathrm{diam}(\Omega) := \sup\{|x-y| : x,y \in \Omega\}$. If $d = 1$, by Bouchitte et al. (2007, Theorem 1.2), we have

$$d_K(\mu,\nu) \leq C_s W_s^{\frac{s}{1+s}}(\mu,\nu),$$

where C_s is a constant depending on $s \geq 1$.

For a function $f : \mathbb{R}^d \to \mathbb{R}$, let $\|f\|_L = \sup_{a\neq b} \frac{|f(a)-f(b)|}{\|a-b\|_2}$ denote the Lipschitz constant of f and let $\|f\|_\infty = \sup_a |f(a)|$. Define norm $\|\cdot\|_{BL}$ for all bounded and Lipschitz continuous functions f as $\|f\|_{BL} := \|f\|_\infty + \|f\|_L$. The bounded Lipschitz distance is given by

$$d_{BL}(X,Y) := \sup_{h:\|h\|_{BL}\leq 1} |\mathbb{E}\{h(X)\} - \mathbb{E}\{h(Y)\}|\,.$$

Further, we can show that the Kolmogorov distance between two pairs of convolutions can be upper bounded by a combination of a bounded Lipschitz metric and Kolmogorov distance between single random variables. We present it as a lemma below.

Lemma 1.1. *Suppose X, Y, $\tilde{X}$ and $\tilde{Y}$ are independent random variables. Assume that $F_{\tilde{Y}}$, the CDF of $\tilde{Y}$, satisfies $\|F_{\tilde{X}}\|_{BL} < \infty$. Then*

$$d_K(\tilde{X} + \tilde{Y}, X + Y) \leq \|F_{\tilde{X}}\|_{BL} d_{BL}(\tilde{Y}, Y) + d_K(\tilde{X}, X).$$

Proof. Let $F_{\tilde{X}}$, F_X, F_Y denotes the CDFs of, respectively, $\tilde{X}$, X and Y. By definition of convolutions and Kolmogorov distance,

$$\begin{aligned}
& d_K\left(\tilde{X} + \tilde{Y}, X + Y\right) \\
&= \sup_{t\in\mathbb{R}} \left| \int_{\mathbb{R}} F_{\tilde{X}}(t-y) \mathrm{d}F_{\tilde{Y}}(y) - \int_{\mathbb{R}} F_X(t-y) \mathrm{d}F_Y(y) \right| \\
&\leq \sup_{t\in\mathbb{R}} \left| \int_{\mathbb{R}} F_{\tilde{X}}(t-y) \mathrm{d}F_{\tilde{Y}}(y) - \int_{\mathbb{R}} F_{\tilde{X}}(t-y) \mathrm{d}F_Y(y) \right| + \sup_{t\in\mathbb{R}} \left| \int_{\mathbb{R}} F_X(t-y) - F_{\tilde{X}}(t-y) \mathrm{d}F_Y(y) \right| \\
&\leq \sup_{t\in\mathbb{R}} \left| \mathbb{E}\left[F_{\tilde{X}}(t-\tilde{Y}) - F_{\tilde{X}}(t-Y) \right] \right| + \sup_{x\in\mathbb{R}} |F_X(x) - F_{\tilde{X}}(x)| \int_{\mathbb{R}} \mathrm{d}F_Y(y) \\
&= \sup_{t\in\mathbb{R}} \left| \mathbb{E}\left[F_{\tilde{X}}(t-\tilde{Y}) - F_{\tilde{X}}(t-Y) \right] \right| + d_K(\tilde{X}, X) \\
&\leq \|F_{\tilde{X}}\|_{BL} \sup_{f:\|f\|_{BL}<\infty} \left| \mathbb{E}\left[f(\tilde{Y}) - f(Y) \right] \right| + d_K(\tilde{X}, X) \\
&= \|F_{\tilde{X}}\|_{BL} d_{BL}(\tilde{Y}, Y) + d_K(\tilde{X}, X).
\end{aligned}$$

□

Lemma 1.2. *Let $\tilde{\theta}_N \in \Theta_N := \{\theta : \|\theta - \theta_\star\| \leq CR_N\}$ for some constant $C > 0$ and a sequence $R_N > 0$ depending on sample size N. If Assumption (A2-b) holds, then there exists an absolute constant $c_2 > 0$ such that*

$$\left| \frac{N^{1/2}\{\nu(\tilde{\theta}_N) - \nu(\theta_\star)\}}{\sigma(\theta_\star)} - \dot{\nu}(\theta_\star)^\top \frac{N^{1/2}\{\tilde{\theta}_N - \theta_\star\}}{\sigma(\theta_\star)} \right| < \frac{c_2 N^{1/2} R_N^2}{\sigma(\theta_\star)}.$$

Proof. By Assumption (A2-b), we have $\|\ddot{\nu}(\theta_\star)\|_2 = \lambda_{\max}(\ddot{\nu}(\theta_\star)) < \infty$. Consider the first-order Taylor expansion of $\nu(\tilde{\theta}_N)$ around $\theta_\star$, for some constant $c_2 > C\lambda_{\max}(\ddot{\nu}(\theta_\star))$ and $\theta' \in \mathcal{U}(\theta_\star, \tilde{\theta}_N) := \{\theta : \|\theta - \theta_\star\| \leq \|\tilde{\theta}_N - \theta_\star\|\}$,

$$\begin{aligned}
\left| \nu(\tilde{\theta}_N) - \nu(\theta_\star) + \dot{\nu}(\theta_\star)(\tilde{\theta}_N - \theta_\star) \right| &= \left| \frac{(\theta' - \theta_\star)^\top \ddot{\nu}(\theta_\star)(\theta' - \theta_\star)}{2} \right| \\
&\leq \frac{1}{2} \lambda_{\max}(\ddot{\nu}(\theta_\star)) \|\theta' - \theta_\star\|^2 \\
&\leq C\lambda_{\max}(\ddot{\nu}(\theta_\star)) R_N^2 \\
&\leq c_2 R_N^2,
\end{aligned}$$

where the first inequality holds since $\ddot{\nu}(\theta_\star)$ is symmetric. The proof is complete after multiplying both sides by $N^{1/2}/\sigma(\theta_\star)$. □

Lemma 1.3. *(Gaussian Concentration, Vershynin (2018, Chap 2.5)) Let $X \sim \mathcal{N}(0, v)$. Then, for all $t \geq 0$,*

$$\mathbb{P}\left[|X| \geq t\right] \leq 2e^{-\frac{t^2}{2v}}.$$

Lemma 1.4. *Let $\Theta_N := \{\theta : \|\theta - \theta_\star\| < C \log^{1/2} N / N^{1/2}\}$. If Assumption A5 holds, then $\mathbb{P}_{\theta_\star}\left[|\Pi(\Theta_N \,|\, \mathbf{X}_N) - 1| > cN^{-1/2}\right] = O(N^{-s/2})$.*

Proof. It follows from Lemma 1.3 and Assumption (A5-a) that $\mathbb{P}_{\theta_\star}[|\widehat{\Pi}(\Theta_N \,|\, \mathbf{X}_N) - 1| > CN^{-1}] = O(N^{-s/2})$. Combining this inequality with Assumption (A5-b) yields

$$\begin{aligned}
&\mathbb{P}_{\theta_\star}\left[|\Pi(\Theta_N \,|\, \mathbf{X}_N) - 1| > cN^{-1/2}\right] \\
&\leq \mathbb{P}_{\theta_\star}\left[\left|\Pi(\Theta_N \,|\, \mathbf{X}_N) - \widehat{\Pi}(\Theta_N \,|\, \mathbf{X}_N)\right| + \left|\widehat{\Pi}(\Theta_N \,|\, \mathbf{X}_N) - 1\right| > cN^{-1/2}\right] \\
&\leq \mathbb{P}_{\theta_\star}\left[d_{TV,\Theta_N}\left(\Pi(\cdot \,|\, \mathbf{X}_N), \widehat{\Pi}(\cdot \,|\, \mathbf{X}_N)\right) > cN^{-1/2} - CN^{-1}\right] + O(N^{-s/2}) \\
&\leq \mathbb{P}_{\theta_\star}\left[d_{TV,\Theta_N}\left(\Pi(\cdot \,|\, \mathbf{X}_N), \widehat{\Pi}(\cdot \,|\, \mathbf{X}_N)\right) > c_{\mathcal{K}}N^{-1/2}\right] + O(N^{-s/2}) \\
&\leq \mathbb{P}_{\theta_\star}\left[d_{TV,\mathcal{K}}\left(\Pi(\cdot \,|\, \mathbf{X}_N), \widehat{\Pi}(\cdot \,|\, \mathbf{X}_N)\right) > c_{\mathcal{K}}N^{-1/2}\right] + O(N^{-s/2}) \\
&= O(N^{-s/2}),
\end{aligned}$$

where the penultimate inequality follows by $d_{\mathrm{TV},\Theta_N} \leq d_{\mathrm{TV},\mathcal{K}}$ since $\Theta_N \subset \mathcal{K}$. □

Lemma 1.5. *If Assumption (A2-c) holds, then for any $\theta \in \Theta_N$ defined in Lemma 1.2,*

$$\left|\frac{\sigma(\theta)}{\sigma(\theta_\star)} - 1\right| \leq \frac{CMR_N}{\sigma(\theta_\star)}.$$

Proof. By Taylor's theorem, for any $\theta \in \Theta_N$, there exists a $\theta' \in \mathcal{U}(\theta_\star, \theta) := \{\theta' : \|\theta' - \theta_\star\| \leq \|\theta - \theta_\star\|\}$ such that

$$\sigma(\theta) = \sigma(\theta_\star) + (\theta - \theta_\star)^\top \dot{\sigma}(\theta'). \tag{1}$$

Since $\Theta_N \subseteq U_{\theta_\star}$, it follows from Assumption (A2-c) together with Eq. (1) that

$$\begin{aligned}
\left|\frac{\sigma(\theta)}{\sigma(\theta_\star)} - 1\right| &= |(\theta - \theta_\star)^\top \dot{\sigma}(\theta')| / \sigma(\theta_\star) \\
&\leq \|\theta - \theta_\star\|_2 \, \|\dot{\sigma}(\theta')\| \, / \sigma(\theta_\star) \\
&\leq \frac{CMR_N}{\sigma(\theta_\star)}.
\end{aligned}$$

□

1.2 Proof of Theorem 3.1

Consider the single SPC p-value as a random variable

$$p_{\mathrm{SPC}}(\mathbf{X}) = \int_\Theta \mathbb{P}\left[T_{N_{\mathrm{ho}}}(\mathbf{X}_{\mathrm{pred}}) \geq T_{N_{\mathrm{ho}}}(\mathbf{X}_{\mathrm{ho}}) \,|\, \theta\right] \Pi(\mathrm{d}\theta \,|\, \mathbf{X}_{\mathrm{obs}}).$$

Let Z denote a standard normal random variable and $\phi(\cdot; \mu, \sigma^2)$ denote the PDF of normal random variables with mean μ and variance σ^2.

Let $\widehat{\pi}(\cdot \mid \mathbf{X}_{\mathrm{obs}}) := \phi(\cdot; \hat{\theta}(\mathbf{X}_{\mathrm{obs}}), J_\star^{-1}/N_{\mathrm{obs}})$ and $\widehat{\Pi}(\cdot \mid \mathbf{X}_{\mathrm{obs}})$ be the corresponding distribution. Denote shrinking parameter space as $\Theta_{N_{\mathrm{obs}}} := \{\theta : \|\theta - \theta_\star\| \le c{N_{\mathrm{obs}}}^{-1/2}\}$. We emphasize on the difference between asymptotic means and standard deviations of T_N by defining $\nu(\theta)$ and $\sigma(\theta)$ as under P_θ and $\nu_\circ$ and $\sigma_\circ$ as under $P_\circ$ respectively. We denote the ratio between asymptotic standard deviations under $P_\circ$ and $P_{\theta_\star}$ as $\kappa := \sigma_\circ/\sigma(\theta_\star)$. For simplicity, we introduce some shorthand notation:

$$
\begin{aligned}
p'_{\mathrm{SPC}}(\mathbf{X}) &:= \int_\Theta \mathbb{P}\left[T_{N_{\mathrm{ho}}}(\mathbf{X}_{\mathrm{pred}}) \ge T_{N_{\mathrm{ho}}}(\mathbf{X}_{\mathrm{ho}}) \mid \theta\right] \widehat{\Pi}(\mathrm{d}\theta \mid \mathbf{X}_{\mathrm{obs}}) \\
p''_{\mathrm{SPC}}(\mathbf{X}) &:= \int_{\Theta_{N_{\mathrm{obs}}}} \mathbb{P}\left[T_{N_{\mathrm{ho}}}(\mathbf{X}_{\mathrm{pred}}) \ge T_{N_{\mathrm{ho}}}(\mathbf{X}_{\mathrm{ho}}) \mid \theta\right] \widehat{\Pi}(\mathrm{d}\theta \mid \mathbf{X}_{\mathrm{obs}}) \\
s_{T\nu}(\mathbf{X}; \theta, N) &:= N^{1/2}\{T_N(\mathbf{X}) - \nu(\theta)\} \\
s_{T\nu_\circ}(\mathbf{X}; N) &:= N^{1/2}\{T_N(\mathbf{X}) - \nu_\circ\} \\
\xi(\theta) &:= \mathbb{P}\big[s_{T\nu}(\mathbf{X}_{\mathrm{pred}}; \theta, N_{\mathrm{ho}}) + N_{\mathrm{ho}}^{1/2}\{\nu(\theta) - \nu(\theta_\star)\} \\
&\qquad\qquad \ge s_{T\nu_\circ}(\mathbf{X}_{\mathrm{pred}}; N_{\mathrm{ho}}) + N_{\mathrm{ho}}^{1/2}\{\nu_\circ - \nu(\theta_\star)\} \mid \theta\big] \\
t(\theta) &:= \frac{s_{T\nu_\circ}(\mathbf{X}_{\mathrm{ho}}; N_{\mathrm{ho}})}{\sigma_\circ}\kappa - \frac{N_{\mathrm{ho}}^{1/2}\dot{\nu}(\theta_\star)\{\theta - \theta_\star\}}{\sigma(\theta_\star)} + \frac{N_{\mathrm{ho}}^{1/2}\{\nu_\circ - \nu(\theta_\star)\}}{\sigma(\theta_\star)} \\
Q(\mathbf{X}_{\mathrm{ho}}, \mathbf{X}_{\mathrm{obs}}) &:= \frac{1}{\tilde{\sigma}}\Bigg(\frac{s_{T\nu_\circ}(\mathbf{X}_{\mathrm{ho}}; N_{\mathrm{ho}})}{\sigma_\circ}\kappa - \frac{N_{\mathrm{ho}}^{1/2}}{N_{\mathrm{obs}}^{1/2}} \frac{N_{\mathrm{obs}}^{1/2}\dot{\nu}(\theta_\star)\{\hat{\theta}(\mathbf{X}_{\mathrm{obs}}) - \theta_\star\}}{\sigma(\theta_\star)} \\
&\qquad\qquad + \frac{N_{\mathrm{ho}}^{1/2}\{\nu_\circ - \nu(\theta_\star)\}}{\sigma(\theta_\star)}\Bigg).
\end{aligned}
$$

Assume that N_{obs} is sufficiently large such that $\Theta_{N_{\mathrm{obs}}} \subseteq \mathcal{U}_{\star,1} \cap \mathcal{U}_{\star,2}$. Letting $t = \Omega(N_{\mathrm{obs}}^{-1/2})$ and $v = O(N_{\mathrm{obs}}^{-1})$ for Lemma 1.3 gives that $\widehat{\Pi}(\Theta_{N_{\mathrm{obs}}} \mid \mathbf{X}_{\mathrm{obs}}) \xrightarrow{P} 1$. By Assumption (A4-b), Lemma 1.3 and the fact that $d_{TV}(\mu, \omega) := 2\sup_{f \in [0,1]} |\int f\mathrm{d}\mu - \int f\mathrm{d}\omega|$, we have

$$
\begin{aligned}
|p_{\mathrm{SPC}}(\mathbf{X}) - p''_{\mathrm{SPC}}(\mathbf{X})| &\le |p_{\mathrm{SPC}}(\mathbf{X}) - p'_{\mathrm{SPC}}(\mathbf{X})| + |p'_{\mathrm{SPC}}(\mathbf{X}) - p''_{\mathrm{SPC}}(\mathbf{X})| \\
&\le d_{TV}\left(\Pi(\cdot \mid \mathbf{X}_{\mathrm{obs}}), \widehat{\Pi}(\cdot \mid \mathbf{X}_{\mathrm{obs}})\right) + \widehat{\Pi}(\Theta_{N_{\mathrm{obs}}}^c \mid \mathbf{X}_{\mathrm{obs}}) \\
&\xrightarrow{P} 0. \qquad (2)
\end{aligned}
$$

By centering both $T_{N_{\mathrm{ho}}}(\mathbf{X}_{\mathrm{pred}})$ and $T_{N_{\mathrm{ho}}}(\mathbf{X}_{\mathrm{ho}})$, the integrand of $p''_{\mathrm{SPC}}(\mathbf{X})$ becomes

$$
\begin{aligned}
&\mathbb{P}\left[T_{N_{\mathrm{ho}}}(\mathbf{X}_{\mathrm{pred}}) \ge T_{N_{\mathrm{ho}}}(\mathbf{X}_{\mathrm{ho}}) \mid \theta\right] \\
&= \mathbb{P}\big[N_{\mathrm{ho}}^{1/2}\{T_{N_{\mathrm{ho}}}(\mathbf{X}_{\mathrm{pred}}) - \nu(\theta)\} + N_{\mathrm{ho}}^{1/2}\{\nu(\theta) - \nu(\theta_\star)\} \\
&\qquad \ge N_{\mathrm{ho}}^{1/2}\{T_{N_{\mathrm{ho}}}(\mathbf{X}_{\mathrm{ho}}) - \nu_\circ\} + N_{\mathrm{ho}}^{1/2}\{\nu_\circ - \nu(\theta_\star)\} \mid \theta\big] \\
&= \mathbb{P}\big[s_{T\nu}(\mathbf{X}_{\mathrm{pred}}; \theta, N_{\mathrm{ho}}) + N_{\mathrm{ho}}^{1/2}\{\nu(\theta) - \nu(\theta_\star)\} \\
&\qquad \ge s_{T\nu_\circ}(\mathbf{X}_{\mathrm{pred}}; N_{\mathrm{ho}}) + N_{\mathrm{ho}}^{1/2}\{\nu_\circ - \nu(\theta_\star)\} \mid \theta\big] \\
&=: \xi(\theta). \qquad (3)
\end{aligned}
$$

Dividing by $1/\sigma(\theta_\star)$ on both sides within the probability and applying Lemmas 1.2 and 1.5 with

$R_N = CN_{\mathrm{obs}}^{-1/2}$ yields

$$\begin{aligned}
\xi(\theta) \leq \mathbb{P}\big[&s_{T\nu}(\mathbf{X}_{\mathrm{pred}};\theta,N_{\mathrm{ho}}) + \frac{N_{\mathrm{ho}}^{1/2}}{N_{\mathrm{obs}}^{1/2}}\left(N_{\mathrm{obs}}^{1/2}\dot{\nu}(\theta_\star)\{\theta-\theta_\star\} + C/N_{\mathrm{obs}}^{1/2}\right) \\
&\qquad > s_{T\nu_\circ}(\mathbf{X}_{\mathrm{ho}};N_{\mathrm{ho}}) + N_{\mathrm{ho}}^{1/2}\{\nu_\circ - \nu(\theta_\star)\}\mid\theta\big] \\
= \mathbb{P}\big[&s_{T\nu}(\mathbf{X}_{\mathrm{pred}};\theta,N_{\mathrm{ho}}) + N_{\mathrm{ho}}^{1/2}\dot{\nu}(\theta_\star)\{\theta-\theta_\star\} + CN_{\mathrm{ho}}^{1/2}/N_{\mathrm{obs}} \\
&\qquad \geq s_{T\nu_\circ}(\mathbf{X}_{\mathrm{ho}};N_{\mathrm{ho}}) + N_{\mathrm{ho}}^{1/2}\{\nu_\circ - \nu(\theta_\star)\}\mid\theta\big] \\
= \mathbb{P}\Bigg[&\frac{s_{T\nu}(\mathbf{X}_{\mathrm{pred}};\theta,N_{\mathrm{ho}})}{\sigma(\theta)}\frac{\sigma(\theta)}{\sigma(\theta_\star)} + \frac{N_{\mathrm{ho}}^{1/2}\dot{\nu}(\theta_\star)\{\theta-\theta_\star\}}{\sigma(\theta_\star)} + \frac{CN_{\mathrm{ho}}^{1/2}/N_{\mathrm{obs}}}{\sigma(\theta_\star)} \\
&\qquad \geq \frac{s_{T\nu_\circ}(\mathbf{X}_{\mathrm{ho}};N_{\mathrm{ho}})}{\sigma_\circ}\frac{\sigma_\circ}{\sigma(\theta_\star)} + \frac{N_{\mathrm{ho}}^{1/2}\{\nu_\circ - \nu(\theta_\star)\}}{\sigma(\theta_\star)}\mid\theta\Bigg] \\
\leq \mathbb{P}\Bigg[&\frac{s_{T\nu}(\mathbf{X}_{\mathrm{pred}};\theta,N_{\mathrm{ho}})}{\sigma(\theta)}\left(1 + \frac{CM}{\sigma(\theta_\star)N_{\mathrm{obs}}^{1/2}}\right) + \frac{N_{\mathrm{ho}}^{1/2}\dot{\nu}(\theta_\star)\{\theta-\theta_\star\}}{\sigma(\theta_\star)} \\
&\qquad + \frac{CN_{\mathrm{ho}}^{1/2}/N_{\mathrm{obs}}}{\sigma(\theta_\star)} \geq \frac{s_{T\nu_\circ}(\mathbf{X}_{\mathrm{ho}};N_{\mathrm{ho}})}{\sigma_\circ}\kappa + \frac{N_{\mathrm{ho}}^{1/2}\{\nu_\circ - \nu(\theta_\star)\}}{\sigma(\theta_\star)}\mid\theta\Bigg] \\
= 1 - \mathbb{P}\Bigg[&\frac{s_{T\nu}(\mathbf{X}_{\mathrm{pred}};\theta,N_{\mathrm{ho}})}{\sigma(\theta)} \leq \frac{s_{T\nu_\circ}(\mathbf{X}_{\mathrm{ho}};N_{\mathrm{ho}})}{\sigma_\circ}\kappa \\
&\qquad - \frac{N_{\mathrm{ho}}^{1/2}\dot{\nu}(\theta_\star)\{\theta-\theta_\star\}}{\sigma(\theta_\star)} + \frac{N_{\mathrm{ho}}^{1/2}\{\nu_\circ - \nu(\theta_\star)\}}{\sigma(\theta_\star)}\mid\theta\Bigg] + o_P(N_{\mathrm{obs}}^{-1/2}) \\
= 1 - \mathbb{P}\Bigg[&\frac{s_{T\nu}(\mathbf{X}_{\mathrm{pred}};\theta,N_{\mathrm{ho}})}{\sigma(\theta)} \leq t(\theta)\mid\theta\Bigg] + o_P(N_{\mathrm{obs}}^{-1/2}).
\end{aligned} \tag{4}$$

By Assumption A3, $\frac{s_{T\nu}(\mathbf{X}_{\mathrm{pred}};\theta,N_{\mathrm{ho}})}{\sigma(\theta)}$, we have

$$\left|\mathbb{P}\left[\frac{s_{T\nu}(\mathbf{X}_{\mathrm{pred}};\theta,N_{\mathrm{ho}})}{\sigma(\theta)} \leq t(\theta)\mid\theta\right] - \Phi\left(t(\theta);\theta\right)\right| \xrightarrow{P} 0. \tag{5}$$

Combining Eqs. (3) to (5) yields

$$\left|\mathbb{P}\left[T_{N_{\mathrm{ho}}}(\mathbf{X}_{\mathrm{pred}}) \geq T_{N_{\mathrm{ho}}}(\mathbf{X}_{\mathrm{ho}})\mid\theta\right] - \left(1 - \Phi\left(t(\theta);\theta\right)\right)\right| \xrightarrow{P} 0,$$

and thus

$$\left|p''_{\mathrm{SPC}}(\mathbf{X}) - \left(1 - \int_{\Theta_{N_{\mathrm{obs}}}} \Phi\left(t(\theta);\theta\right)\widehat{\Pi}(\mathrm{d}\theta\mid\mathbf{X}_{\mathrm{obs}})\right)\right| \xrightarrow{P} 0, \tag{6}$$

Let $\mathbb{E}_\theta[\cdot]$ denote the expectation with respect to $\phi(\hat{\theta}(\mathbf{X}_{\mathrm{obs}}), J_\star^{-1}\Sigma_\star J_\star^{-1}/N_{\mathrm{obs}})$. Let Z be an independent standard normal random variable and define

$$\tilde{Z} := Z + \frac{N_{\mathrm{ho}}^{1/2}}{N_{\mathrm{obs}}^{1/2}}\frac{N_{\mathrm{obs}}^{1/2}\dot{\nu}(\theta_\star)\{\theta - \hat{\theta}(\mathbf{X}_{\mathrm{obs}})\}}{\sigma(\theta_\star)} \sim \mathcal{N}(0,\tilde{\sigma}^2),$$

where $\tilde{\sigma}^2 := 1 + \frac{\frac{N_{\text{ho}}}{N_{\text{obs}}}\dot{\nu}(\theta_\star)^2 J_\star^{-1}}{\sigma(\theta_\star)^2}$. Consider

$$
\begin{aligned}
&\int_{\Theta_{N_{\text{obs}}}} \Phi\left(t(\theta);\theta\right) \widehat{\Pi}(\mathrm{d}\theta \mid \mathbf{X}_{\text{obs}}) \\
&= \mathbb{E}_\theta\left[\mathbb{P}_Z\left[Z \le \frac{s_{T\nu_\circ}(\mathbf{X}_{\text{ho}}; N_{\text{ho}})}{\sigma_\circ}\kappa - \frac{N_{\text{ho}}^{1/2}\dot{\nu}(\theta_\star)\{\theta-\theta_\star\}}{\sigma(\theta_\star)} + \frac{N_{\text{ho}}^{1/2}\{\nu_\circ - \nu(\theta_\star)\}}{\sigma(\theta_\star)}\right]\right] \\
&= \mathbb{P}_{Z,\theta}\left[Z \le \frac{s_{T\nu_\circ}(\mathbf{X}_{\text{ho}}; N_{\text{ho}})}{\sigma_\circ}\kappa - \frac{N_{\text{ho}}^{1/2}\dot{\nu}(\theta_\star)\{\theta-\theta_\star\}}{\sigma(\theta_\star)} + \frac{N_{\text{ho}}^{1/2}\{\nu_\circ - \nu(\theta_\star)\}}{\sigma(\theta_\star)}\right] \\
&= \mathbb{P}_{Z,\theta}\Bigg[Z + \frac{N_{\text{ho}}^{1/2}}{N_{\text{obs}}^{1/2}}\frac{N_{\text{obs}}^{1/2}\dot{\nu}(\theta_\star)\{\theta-\hat{\theta}(\mathbf{X}_{\text{obs}})\}}{\sigma(\theta_\star)} \\
&\qquad \le \frac{s_{T\nu_\circ}(\mathbf{X}_{\text{ho}}; N_{\text{ho}})}{\sigma_\circ}\kappa - \frac{N_{\text{ho}}^{1/2}}{N_{\text{obs}}^{1/2}}\frac{N_{\text{obs}}^{1/2}\dot{\nu}(\theta_\star)\{\hat{\theta}(\mathbf{X}_{\text{obs}})-\theta_\star\}}{\sigma(\theta_\star)} + \frac{N_{\text{ho}}^{1/2}\{\nu_\circ - \nu(\theta_\star)\}}{\sigma(\theta_\star)}\Bigg] \\
&= \mathbb{P}_{\tilde{Z}}\left[\frac{\tilde{Z}}{\tilde{\sigma}} \le Q(\mathbf{X}_{\text{ho}}, \mathbf{X}_{\text{obs}})\right] \\
&= \Phi\left(Q(\mathbf{X}_{\text{ho}}, \mathbf{X}_{\text{obs}})\right). \qquad (7)
\end{aligned}
$$

Combining Eqs. (2), (6) and (7) yields the asymptotic approximation

$$p_{\text{SPC}}(\mathbf{X}) = 1 - \Phi\left(Q(\mathbf{X}_{\text{ho}}, \mathbf{X}_{\text{obs}})\right) + o_P(1).$$

Case 1: $\nu_\circ = \nu(\theta_\star)$. Since $\mathbf{X}_{\text{obs}}$ and $\mathbf{X}_{\text{ho}}$ are independent, we have by Assumptions A3 and (A4-a) that

$$Q(\mathbf{X}_{\text{ho}}, \mathbf{X}_{\text{obs}}) \xrightarrow{\mathcal{D}} \mathcal{N}(0, \rho^2).$$

Hence for any $\alpha \in (0,1)$, we have

$$
\begin{aligned}
\mathbb{P}\left[p_{\text{SPC}}(\mathbf{X}) < \alpha\right] &= \mathbb{P}\left[1 - \Phi\left(Q(\mathbf{X}_{\text{ho}}, \mathbf{X}_{\text{obs}})\right) < \alpha\right] + o_P(1) \\
&= \mathbb{P}\left[Q(\mathbf{X}_{\text{ho}}, \mathbf{X}_{\text{obs}}) > z_{1-\alpha}\right] + o_P(1) \\
&= \mathbb{P}\left[\frac{Q(\mathbf{X}_{\text{ho}}, \mathbf{X}_{\text{obs}})}{\rho} > \frac{z_{1-\alpha}}{\rho}\right] + o_P(1) \\
&= 1 - \Phi\left(\frac{z_{1-\alpha}}{\rho}\right) + o_P(1),
\end{aligned}
$$

where z_α satisfies $\mathbb{P}[Z \le z_\alpha] = \alpha$.

Case 2: $\nu_\circ < \nu(\theta_\star)$. Taking $N_{\text{ho}} \to \infty$ yields $\mathbb{P}(Q(\mathbf{X}_{\text{ho}}, \mathbf{X}_{\text{obs}}) > t) \to 0, \forall t \in \mathbb{R}$ and thus $p_{\text{SPC}}(\mathbf{X}) \xrightarrow{P} 1$. Therefore, the tail probabilities of single SPC p-values are immediate

$$
\begin{aligned}
\mathbb{P}\left[p_{\text{SPC}}(\mathbf{X}) > 1 - \alpha\right] &= \mathbb{P}\left[1 - \Phi\left(Q(\mathbf{X}_{\text{ho}}, \mathbf{X}_{\text{obs}})\right) > 1 - \alpha\right] + o_P(1) \\
&= 1 + o_P(1).
\end{aligned}
$$

Case 3: $\nu_\circ > \nu(\theta_\star)$. Similarly to Case 2, the single SPC p-values converge to 0 in probability and thus $\mathbb{P}\left[p_{\text{SPC}}(\mathbf{X}) < \alpha\right] \xrightarrow{P} 1$.

1.3 Proof of Theorem A.1

Theorem A.1 gives the rate of convergence results for single SPC p-values to uniform distribution under a well-specified model. In addition to the regularity conditions in Assumption A2, we require stronger versions of Assumptions A3 and A4. First, we require the scaled test statistic satisfy a CLT at rate $N^{-1/2}$ in Kolmogorov distance d_K.

Assumption A6. *There exists an open neighborhood $U_{\star,6} \ni \theta_\star$ and an absolute constant $c_1 > 0$ such that for $\theta \in U_{\star,6}$ and $Y_1, \dots, Y_N \overset{i.i.d.}{\sim} P_\theta$,*

$$d_K\left(\frac{N^{1/2}\{T_N(\mathbf{Y}_N) - \nu(\theta)\}}{\sigma(\theta)}, Z\right) < c_1/N^{1/2}, \tag{8}$$

where Z is a standard normal random variable.

If the statistic takes the form of an average, $T_N(\mathbf{X}) = \frac{1}{N}\sum_{n=1}^N h(X_n)$, the assumption holds by the Berry–Esseen theorem as long as $\sup_{\theta \in U_{\star,6}} \mathbb{E}_\theta[|h(X_1)|^3] < \infty$ (Gut 2013, Theorem 6.1). The assumption also holds for U-statistics $T_N(\mathbf{X}) = \frac{2}{N(N-1)}\sum_{i<j} h(X_i, X_j)$ as long as $\sup_{\theta \in U_{\star,6}} \mathbb{E}[|h(X_1, X_2)|^3] < \infty$ (Alberink 1999).

We also require the maximum likelihood to be asymptotically normal in the bounded Lipschitz metric.

Assumption A7. *For $Z \sim \mathcal{N}(0, I)$, the maximum likelihood estimator satisfies*

$$d_{BL}(N^{1/2}\Sigma_\star^{-1}\{\hat\theta(\mathbf{X}_N) - \theta_\star\}, Z) = O(N^{-1/2}).$$

Assumption A7 can be shown to hold using, for example, Anastasiou & Ley (2017, Lemma 2.1).

Proof of Theorem A.1. We will first prove convergence in s-Wasserstein distance, which we will then use to control convergence in Kolmogorov distance.

Given data $\mathbf{X}$, the single SPC p-value is defined as

$$p_{\text{SPC}}(\mathbf{X}) = \int_\Theta \mathbb{P}\left[T_{N_{\text{ho}}}(\mathbf{X}_{\text{pred}}) \geq T_{N_{\text{ho}}}(\mathbf{X}_{\text{ho}}) \,|\, \theta\right] \Pi(\mathrm{d}\theta \,|\, \mathbf{X}_{\text{obs}}).$$

Let Z denote a standard normal random variable and $\phi(\cdot; \mu, \sigma^2)$ denote the PDF of a normal random variables with mean μ and variance σ^2. Suppose $\mathcal{K}$ is a compact set containing $\theta_\star$ and a shrinking parameter space is defined as $\Theta_{N_{\text{obs}}} := \{\theta : \|\theta - \theta_\star\| \leq C \log^{1/2} N_{\text{obs}}/{N_{\text{obs}}}^{1/2}\}$. Assume that N_{obs} is sufficiently large that $\Theta_{N_{\text{obs}}} \subseteq \mathcal{K} \cap \mathcal{U}_{\star,1} \cap \mathcal{U}_{\star,6}$. Denote $\widehat\pi(\cdot \,|\, \mathbf{X}_{\text{obs}}) := \phi(\cdot; \hat\theta(\mathbf{X}_{\text{obs}}), \Sigma_\star/N_{\text{obs}})$ and

$\widehat{\Pi}(\cdot \mid \mathbf{X}_{\text{obs}})$ the corresponding distribution. For simplicity, we introduce some shorthand notation:

$$
\begin{aligned}
p'_{\text{SPC}}(\mathbf{X}) &:= \int_{\Theta_{N_{\text{obs}}}} \mathbb{P}[T_{N_{\text{ho}}}(\mathbf{X}_{\text{pred}}) \geq T_{N_{\text{ho}}}(\mathbf{X}_{\text{ho}}) \mid \theta] \Pi(\mathrm{d}\theta \mid \mathbf{X}_{\text{obs}}) \\
p''_{\text{SPC}}(\mathbf{X}) &:= \int_{\Theta_{N_{\text{obs}}}} \mathbb{P}[T_{N_{\text{ho}}}(\mathbf{X}_{\text{pred}}) \geq T_{N_{\text{ho}}}(\mathbf{X}_{\text{ho}}) \mid \theta] \widehat{\Pi}(\mathrm{d}\theta \mid \mathbf{X}_{\text{obs}}) \\
s_{T\nu}(\mathbf{X}, \theta; N) &:= N^{1/2}\{T_N(\mathbf{X}) - \nu(\theta)\} \\
t(\theta) &:= N_{\text{ho}}^{1/2}\{T_{N_{\text{ho}}}(\mathbf{X}_{\text{ho}}) - \nu(\theta_\star)\} - N_{\text{ho}}^{1/2}\{\nu(\theta) - \nu(\theta_\star)\} \\
\tilde{Q}(\theta) &:= \frac{s_{T\nu}(\mathbf{X}_{\text{ho}}, \theta_\star; N_{\text{ho}}) - \dot{\nu}(\theta_\star)^\top N_{\text{ho}}^{1/2}(\theta - \theta_\star)}{\sigma(\theta_\star)} \\
Q &:= \frac{s_{T\nu}(\mathbf{X}_{\text{ho}}, \theta_\star; N_{\text{ho}}) - \dot{\nu}(\theta_\star)^\top N_{\text{ho}}^{1/2}\{\hat{\theta}(\mathbf{X}_{\text{obs}}) - \theta_\star\}}{(\sigma^2(\theta_\star) + \frac{N_{\text{ho}}}{N_{\text{obs}}}\dot{\nu}(\theta_\star)^\top \Sigma(\theta_\star)\dot{\nu}(\theta_\star))^{1/2}} \\
\zeta(\theta) &:= \mathbb{P}\left[s_{T\nu}(\mathbf{X}_{\text{pred}}, \theta; N_{\text{ho}}) \geq t(\theta) \mid \theta\right].
\end{aligned}
$$

Here $p'_{\text{SPC}}(\mathbf{X})$ approximates the single SPC p-values by restricting the integral to $\Theta_{N_{\text{obs}}}$ and then $p''_{\text{SPC}}(\mathbf{X})$ defines a further approximation with standard posterior replaced by a limiting Gaussian density $\widehat{\Pi}(\cdot \mid \mathbf{X}_{\text{obs}})$. We use $t(\theta), \tilde{Q}(\theta)$ and Q in intermediate steps in our proof and that they are quantities approximating p-values using assumptions listed above. For any real-valued function $g(\theta)$, define the shorthands $\widehat{\int} g(\theta) := \int_{\Theta_{N_{\text{obs}}}} g(\theta)\widehat{\Pi}(\mathrm{d}\theta \mid \mathbf{X}_{\text{obs}})$ and $\widehat{\int}_\Theta g(\theta) := \int_\Theta g(\theta)\widehat{\Pi}(\mathrm{d}\theta \mid \mathbf{X}_{\text{obs}})$.

Let $\Phi(\cdot)$ be the CDF of a standard normal random variables. Applying triangle inequalities, we can divide the problem into several parts and bound each part in turn.

$$
\begin{aligned}
&W_s\left(p_{\text{SPC}}(\mathbf{X}), U\right) = W_s\left(p_{\text{SPC}}(\mathbf{X}), \Phi(Z)\right) \\
&\leq W_s\left(p_{\text{SPC}}(\mathbf{X}), p'_{\text{SPC}}(\mathbf{X})\right) + W_s\left(p'_{\text{SPC}}(\mathbf{X}), p''_{\text{SPC}}(\mathbf{X})\right) \\
&\quad + W_s\left(p''_{\text{SPC}}(\mathbf{X}), \widehat{\int}\zeta(\theta)\right) + W_s\left(\widehat{\int}\zeta(\theta), \widehat{\int}\Phi\left(\frac{t(\theta)}{\sigma(\theta)}\right)\right) \\
&\quad + W_s\left(\widehat{\int}\Phi\left(\frac{t(\theta)}{\sigma(\theta)}\right), \widehat{\int}\Phi\left(\frac{t(\theta)}{\sigma(\theta_\star)}\right)\right) + W_s\left(\widehat{\int}\Phi\left(\frac{t(\theta)}{\sigma(\theta_\star)}\right), \widehat{\int}\Phi\left(\tilde{Q}(\theta)\right)\right) \\
&\quad + W_s\left(\widehat{\int}\Phi\left(\tilde{Q}(\theta)\right), \Phi(Q)\right) + W_s(\Phi(Q), \Phi(Z)).
\end{aligned}
$$

Part I: $W_s\left(p_{\text{SPC}}(\mathbf{X}), p'_{\text{SPC}}(\mathbf{X})\right) = O(N_{\text{obs}}^{-1/2})$.

We first show that the integral that defines SPC p-values can be restricted to a shrinking ball while only introducing an error of order $O(N_{\text{obs}}^{-1/2})$ in probability. Fix constants $C > 0$ and

$c > c_{\mathcal{K}} + C$. It follows by the coupling characterization of Wasserstein distance that

$$\begin{aligned}
&W_s^s\left(p_{\text{SPC}}(\mathbf{X}), p'_{\text{SPC}}(\mathbf{X})\right)\\
&\leq \mathbb{E}\left|p_{\text{SPC}}(\mathbf{X}) - p'_{\text{SPC}}(\mathbf{X})\right|^s\\
&= \mathbb{E}\left|\int_{\Theta\setminus\Theta_{N_{\text{obs}}}} \mathbb{P}[T_{N_{\text{ho}}}(\mathbf{X}_{\text{pred}}) \geq T_{N_{\text{ho}}}(\mathbf{X}_{\text{ho}}) \mid \theta]\Pi(\mathrm{d}\theta \mid \mathbf{X}_{\text{obs}})\right|^s\\
&\leq \mathbb{E}\left|1 - \Pi(\Theta_{N_{\text{obs}}} \mid \mathbf{X}_{\text{obs}})\right|^s\\
&\leq cN_{\text{obs}}^{-s/2} + \mathbb{P}_{\theta_\star}[|\Pi(\Theta_{N_{\text{obs}}} \mid \mathbf{X}_{\text{obs}}) - 1| > cN_{\text{obs}}^{-1/2}]\\
&\leq c' N_{\text{obs}}^{-s/2}
\end{aligned} \tag{9}$$

where the last inequality follows by applying Lemma 1.4 with $\mathbf{X}_{\text{obs}}$ of size N_{obs}.

Part II: $W_s\left(p_{\text{SPC}}(\mathbf{X}), p''_{\text{SPC}}(\mathbf{X})\right) = O(N_{\text{obs}}^{-1/2})$.

Consider the equivalent definition of total variation distance, i.e., $d_{TV}(\mu,\nu) = 2\sup_f\{|\int f\mathrm{d}\mu - \int f\mathrm{d}\nu| : 0 \leq f \leq 1\}$. A similar argument as used in Eq. (9) can be employed to get

$$\begin{aligned}
W_s^s\left(p'_{\text{SPC}}(\mathbf{X}), p''_{\text{SPC}}(\mathbf{X})\right) &\leq \mathbb{E}\left|p'_{\text{SPC}}(\mathbf{X}) - p''_{\text{SPC}}(\mathbf{X})\right|^s\\
&\leq \mathbb{E}_\theta\left[d^s_{TV,\Theta_{N_{\text{obs}}}}(\Pi(\cdot \mid \mathbf{X}), \widehat{\Pi}(\cdot \mid \mathbf{X}))\right]\\
&\leq c_{\mathcal{K}} N_{\text{obs}}^{-s/2} + O(N_{\text{obs}}^{-s/2}).
\end{aligned}$$

Therefore,

$$W_s\left(p'_{\text{SPC}}(\mathbf{X}), p''_{\text{SPC}}(\mathbf{X})\right) = O(N_{\text{obs}}^{-1/2}).$$

Part III: $W_s\left(p''_{\text{SPC}}(\mathbf{X}), \widehat{\int}\Phi\left(\frac{t(\theta)}{\sigma(\theta)}\right)\right) = O(N_{\text{obs}}^{-1/2})$. Starting from Part III, we mainly approximate the integrand of single SPC p-values. In this part, we first rewrite the integrand using our shorthand notations defined earlier. By definition, $p''_{\text{SPC}}(\mathbf{X}) = \widehat{\int}\mathbb{P}[T_{N_{\text{ho}}}(\mathbf{X}_{\text{pred}}) \geq T_{N_{\text{ho}}}(\mathbf{X}_{\text{ho}}) \mid \theta]$. But since

$$\begin{aligned}
&\mathbb{P}[T_{N_{\text{ho}}}(\mathbf{X}_{\text{pred}}) \geq T_{N_{\text{ho}}}(\mathbf{X}_{\text{ho}}) \mid \theta]\\
&= \mathbb{P}[N_{\text{ho}}^{1/2}\{T_{N_{\text{ho}}}(\mathbf{X}_{\text{pred}}) - \nu(\theta_\star)\} - N_{\text{ho}}^{1/2}\{\nu(\theta) - \nu(\theta_\star)\}\\
&\qquad \geq N_{\text{ho}}^{1/2}\{T_{N_{\text{ho}}}(\mathbf{X}_{\text{pred}}) - \nu(\theta_\star)\} - N_{\text{ho}}^{1/2}\{\nu(\theta) - \nu(\theta_\star)\} \mid \theta]\\
&= \mathbb{P}[N_{\text{ho}}^{1/2}\{T_{N_{\text{ho}}}(\mathbf{X}_{\text{pred}}) - \nu(\theta)\} \geq t(\theta) \mid \theta]\\
&= \mathbb{P}[s_{T\nu}(X_{\text{pred}}, \theta; N_{\text{ho}}) \geq t(\theta) \mid \theta]\\
&= \zeta(\theta),
\end{aligned}$$

the desired equality follows. Now we only need to show that $d_W\left(\widehat{\int}\zeta(\theta), \widehat{\int}\Phi\left(\frac{t(\theta)}{\sigma(\theta)}\right)\right) = O(N_{\text{obs}}^{-1/2})$. By definition of $N_{\text{obs}} := \lceil qN \rceil$ and $N_{\text{ho}} := N - N_{\text{obs}}$, $N_{\text{ho}}^{1/2}/N_{\text{obs}}^{1/2} = \sqrt{N/N_{\text{obs}} - 1} > c_0 > 0$. Since

$$\zeta(\theta) = \mathbb{P}\left[s_{T\nu}(\mathbf{X}_{\text{pred}}, \theta; N_{\text{ho}}) \geq t(\theta) \mid \theta\right] = \mathbb{P}\left[\frac{s_{T\nu}(\mathbf{X}_{\text{pred}}, \theta; N_{\text{ho}})}{\sigma(\theta)} \geq \frac{t(\theta)}{\sigma(\theta)} \mid \theta\right]$$

it follows from Eq. (8) that for some constant $c_1 > 0$,

$$\sup_{\theta\in\Theta_{N_{\text{obs}}}}\left|\mathbb{P}\left[\frac{s_{T\nu}(\mathbf{X}_{\text{pred}}, \theta; N_{\text{ho}})}{\sigma(\theta)} \geq \frac{t(\theta)}{\sigma(\theta)} \mid \theta\right] - \Phi\left(\frac{t(\theta)}{\sigma(\theta)}\right)\right| < c_1/N_{\text{obs}}^{1/2}. \tag{10}$$

It then follows by the coupling characterization of Wasserstein distance and Eq. (10) that

$$
\begin{aligned}
&W_s^s\left(\widehat{\int}\zeta(\theta), \widehat{\int}\Phi\left(\frac{t(\theta)}{\sigma(\theta)}\right)\right) \\
&\leq \mathbb{E}\left|\widehat{\int}\zeta(\theta) - \widehat{\int}\Phi\left(\frac{t(\theta)}{\sigma(\theta)}\right)\right|^s \\
&\leq \mathbb{E}\left[\widehat{\int}\left|\mathbb{P}\left[\frac{s_{T\nu}(\mathbf{X}_{\mathrm{pred}}, \theta; N_{\mathrm{ho}})}{\sigma(\theta)} \geq \frac{t(\theta)}{\sigma(\theta)}; \theta\right] - \Phi\left(\frac{t(\theta)}{\sigma(\theta)}\right)\right|\right]^s \\
&\leq \mathbb{E}\left[\int_{\Theta_{N_{\mathrm{obs}}}} \sup_{\theta\in\Theta_{N_{\mathrm{obs}}}}\left|\mathbb{P}\left[\frac{s_{T\nu}(\mathbf{X}_{\mathrm{pred}}, \theta; N_{\mathrm{ho}})}{\sigma(\theta)} \geq \frac{t(\theta)}{\sigma(\theta)}; \theta\right] - \Phi\left(\frac{t(\theta)}{\sigma(\theta)}\right)\right|\widehat{\Pi}(\mathrm{d}\theta \mid \mathbf{X}_{\mathrm{obs}})\right]^s \\
&\leq c_1/N_{\mathrm{obs}}^{s/2}\mathbb{E}\left[\widehat{\Pi}(\Theta_{N_{\mathrm{obs}}} \mid \mathbf{X}_{\mathrm{obs}})\right]^s \\
&\leq c_1/N_{\mathrm{obs}}^{s/2}.
\end{aligned}
$$

Part IV: $W_s\left(\widehat{\int}\Phi\left(\frac{t(\theta)}{\sigma(\theta)}\right), \widehat{\int}\Phi\left(\frac{t(\theta)}{\sigma(\theta_\star)}\right)\right) = O(\log N_{\mathrm{obs}}/N_{\mathrm{obs}}^{1/2})$.

We have

$$
\begin{aligned}
&W_s^s\left(\widehat{\int}\Phi\left(\frac{t(\theta)}{\sigma(\theta)}\right), \widehat{\int}\Phi\left(\frac{t(\theta)}{\sigma(\theta_\star)}\right)\right) \\
&\leq \mathbb{E}\left[\widehat{\int}\left|\Phi\left(\frac{t(\theta)}{\sigma(\theta)}\right) - \Phi\left(\frac{t(\theta)}{\sigma(\theta_\star)}\right)\right|\right]^s \\
&\leq \|\Phi\|_L^s\mathbb{E}\left[\int_{\Theta_{N_{\mathrm{obs}}}}\left|\frac{t(\theta)}{\sigma(\theta)}\left(1 - \frac{\sigma(\theta)}{\sigma(\theta_\star)}\right)\right|\widehat{\Pi}(\mathrm{d}\theta \mid \mathbf{X}_{\mathrm{obs}})\right]^s,
\end{aligned}
$$

where $\|\Phi\|_L$ denotes the Lipschitz constant of Φ. By Lemma 1.5, the distance can be further bounded as

$$
\begin{aligned}
&W_s\left(\widehat{\int}\Phi\left(\frac{t(\theta)}{\sigma(\theta)}\right), \widehat{\int}\Phi\left(\frac{t(\theta)}{\sigma(\theta_\star)}\right)\right) \\
&\leq \frac{\|\Phi\|_L CM\log^{1/2}N_{\mathrm{obs}}}{\sigma(\theta_\star)N_{\mathrm{obs}}^{1/2}}\mathbb{E}\left[\int_{\Theta_{N_{\mathrm{obs}}}}\left|\frac{t(\theta)}{\sigma(\theta)}\right|\widehat{\Pi}(\mathrm{d}\theta \mid \mathbf{X}_{\mathrm{obs}})\right].
\end{aligned}
\tag{11}
$$

Notice that $(\mathbf{X}_{\text{obs}}, \mathbf{X}_{\text{ho}})$ are jointly independent. Applying Lemmas 1.2 and 1.5, we have

$$
\begin{aligned}
\sup_{\theta \in \Theta_{N_{\text{obs}}}} \mathbb{E}\left[\frac{t(\theta)}{\sigma(\theta)}\right] &= \sup_{\theta \in \Theta_{N_{\text{obs}}}} \mathbb{E}\left[\frac{N_{\text{ho}}^{1/2}\{T_{N_{\text{ho}}}(\mathbf{X}_{\text{ho}}) - \nu(\theta)\}}{\sigma(\theta)}\right] \\
&= \sup_{\theta \in \Theta_{N_{\text{obs}}}} \frac{N_{\text{ho}}^{1/2}\{\mathbb{E}\left[T_{N_{\text{ho}}}(\mathbf{X}_{\text{ho}})\right] - \nu(\theta)\}}{\sigma(\theta)} \\
&= \sup_{\theta \in \Theta_{N_{\text{obs}}}} \frac{N_{\text{ho}}^{1/2}\{\nu(\theta_\star) - \nu(\theta)\}}{\sigma(\theta_\star)} \frac{\sigma(\theta_\star)}{\sigma(\theta)} \\
&\leq \left(\sup_{\theta \in \Theta_{N_{\text{obs}}}} \dot{\nu}(\theta_\star) \frac{N_{\text{ho}}^{1/2}\{\theta_\star - \theta\}}{\sigma(\theta_\star)} + c_2 \log N_{\text{ho}}/N_{\text{ho}}^{1/2}\right) \\
&\qquad\qquad \times \left(1 + \frac{CM \log^{1/2} N_{\text{obs}}}{\sigma(\theta_\star) N_{\text{obs}}^{1/2}}\right) \\
&\leq C_1 \log^{1/2} N_{\text{ho}} + C_2 \log N_{\text{ho}}/N_{\text{ho}}^{1/2} \\
&\qquad + C_3 \log N_{\text{ho}}/N_{\text{ho}}^{1/2} + C_4 \log^{3/2} N_{\text{ho}}/N_{\text{ho}} \\
&\leq \tilde{C} \log^{1/2} N_{\text{ho}}. \qquad (12)
\end{aligned}
$$

It then follows by Fubini's theorem, and Eqs. (11) and (12) that

$$
\begin{aligned}
&W_s\left(\widehat{\int} \Phi\left(\frac{t(\theta)}{\sigma(\theta)}\right), \widehat{\int} \Phi\left(\frac{t(\theta)}{\sigma(\theta_\star)}\right)\right) \\
&\leq \frac{\|\Phi\|_L CM \log^{1/2} N_{\text{obs}}}{\sigma(\theta_\star) N_{\text{obs}}^{1/2}} \tilde{C} \log^{1/2} N_{\text{ho}} \\
&= C' \log N_{\text{obs}}/N_{\text{obs}}^{1/2}.
\end{aligned}
$$

Part V: $W_s\left(\widehat{\int} \Phi\left(\frac{t(\theta)}{\sigma(\theta_\star)}\right), \widehat{\int} \Phi\left(\tilde{Q}(\theta)\right)\right) = O(\log N_{\text{obs}}/N_{\text{obs}}^{1/2})$

By definition of $t(\theta)$ and $\tilde{Q}(\theta)$, we have

$$
\left|\frac{t(\theta)}{\sigma(\theta_\star)} - \tilde{Q}(\theta)\right| = \left|\frac{N_{\text{ho}}^{1/2}\{\nu(\theta) - \nu(\theta_\star)\}}{\sigma(\theta_\star)} - \dot{\nu}(\theta_\star)^\top \frac{N_{\text{ho}}^{1/2}\{\theta - \theta_\star\}}{\sigma(\theta_\star)}\right|.
$$

Applying Lemmas 1.2 and 1.5, we conclude that for some constant $c_2 > 0$,

$$\begin{aligned}
& d_W^s\left(\widehat{\int}\Phi\left(\frac{t(\theta)}{\sigma(\theta_\star)}\right), \widehat{\int}\Phi\left(\tilde{Q}(\theta)\right)\right) \\
& \leq \mathbb{E}\left|\widehat{\int}\Phi\left(\frac{t(\theta)}{\sigma(\theta_\star)}\right) - \Phi\left(\tilde{Q}(\theta)\right)\right|^s \\
& \leq \mathbb{E}\left[\int_{\Theta_{N_{\text{obs}}}}\left|\Phi\left(\frac{t(\theta)}{\sigma(\theta_\star)}\right) - \Phi\left(\tilde{Q}(\theta)\right)\right|\widehat{\Pi}(\mathrm{d}\theta \mid \mathbf{X}_{\text{obs}})\right]^s \\
& \leq \|\phi\|_\infty^s \mathbb{E}\left[\int_{\Theta_{N_{\text{obs}}}}\left|\frac{t(\theta)}{\sigma(\theta_\star)} - \tilde{Q}(\theta)\right|\widehat{\Pi}(\mathrm{d}\theta \mid \mathbf{X}_{\text{obs}})\right]^s \\
& \leq \|\phi\|_\infty^s c_2 \log^s N_{\text{obs}}/N_{\text{obs}}^{s/2}\mathbb{E}\left[\widehat{\Pi}(\Theta_{N_{\text{obs}}} \mid \mathbf{X}_{\text{obs}})\right]^s \\
& \leq \|\phi\|_\infty^s c_2 \log^s N_{\text{obs}}/N_{\text{obs}}^{s/2}.
\end{aligned}$$

Part VI: $W_s\left(\widehat{\int}\Phi\left(\tilde{Q}(\theta)\right), \Phi(Q)\right) = O(N_{\text{obs}}^{-1/2})$.

Letting $\vartheta \sim \mathcal{N}(\hat{\theta}(\mathbf{X}_{\text{obs}}), \Sigma_\star/N_{\text{obs}})$, we have

$$\begin{aligned}
\widehat{\int}_\Theta \Phi\left(\tilde{Q}(\theta)\right) &= \int_\Theta \Phi\left(\tilde{Q}(\theta)\right)\widehat{\Pi}(\mathrm{d}\theta \mid \mathbf{X}_{\text{obs}}) \\
&= \mathbb{E}\left[\Phi(\tilde{Q}(\vartheta))\right] \\
&= \mathbb{E}\left\{\mathbb{P}\left[Z \leq \frac{s_{T\nu}(\mathbf{X}_{\text{ho}}, \theta_\star; N_{\text{ho}}) - \dot{\nu}(\theta_\star)^\top N_{\text{ho}}^{1/2}(\vartheta - \theta_\star)}{\sigma(\theta_\star)}\right]\right\} \\
&= \mathbb{E}\left\{\mathbb{P}\left[\underbrace{Z\sigma(\theta_\star) + \dot{\nu}(\theta_\star)^\top N_{\text{ho}}^{1/2}(\vartheta - \theta_\star)}_{=:Z' \sim \mathcal{N}(\mu_{Z'}, \sigma_{Z'}^2)} \leq s_{T\nu}(\mathbf{X}_{\text{ho}}, \theta_\star; N_{\text{ho}})\right]\right\} \\
&= \mathbb{P}\left[\frac{Z' - \mu_{Z'}}{\sigma_{Z'}} \leq \underbrace{\frac{s_{T\nu}(\mathbf{X}_{\text{ho}}, \theta_\star; N_{\text{ho}}) - \mu_{Z'}}{\sigma_{Z'}}}_{=:Q}\right],
\end{aligned}$$

where $\mu_{Z'} := \dot{\nu}(\theta_\star)^\top N_{\text{ho}}^{1/2}(\hat{\theta}(\mathbf{X}_{\text{obs}}) - \theta_\star)$ and $\sigma_{Z'}^2 := \sigma^2(\theta_\star) + \frac{N_{\text{ho}}}{N_{\text{obs}}}\dot{\nu}(\theta_\star)^\top \Sigma(\theta_\star)\dot{\nu}(\theta_\star)$. It remains to show that $W_s\left(\widehat{\int}\Phi\left(\tilde{Q}(\theta)\right), \widehat{\int}_\Theta\Phi\left(\tilde{Q}(\theta)\right)\right) = O(N_{\text{obs}}^{-1/2})$. We apply Lemma 1.4 and Assumption (A5-b) to get

$$\begin{aligned}
& W_s^s\left(\widehat{\int}\Phi\left(\tilde{Q}(\theta)\right), \widehat{\int}_\Theta\Phi\left(\tilde{Q}(\theta)\right)\right) \\
& \leq \mathbb{E}\left[\left|\int_{\Theta\setminus\Theta_{N_{\text{obs}}}}\Phi(\tilde{Q}(\theta))\right|\widehat{\Pi}(\mathrm{d}\theta \mid \mathbf{X}_{\text{obs}})\right]^s \\
& \leq \mathbb{E}\left[\left|1 - \widehat{\Pi}(\Theta_{N_{\text{obs}}} \mid \mathbf{X}_{\text{obs}})\right|^s\right] \\
& \leq CN_{\text{obs}}^{-s/2}.
\end{aligned}$$

Part VII: $W_s(\Phi(Q),\Phi(Z)) = O(N_{\text{obs}}^{-1/2})$.

Since Φ is a Lipschitz-continuous function and $\Phi(\cdot)\in[0,1]$, there exists some constant $C>0$ such that

$$W_s(\Phi(Q),\Phi(Z)) \le C d_K(\Phi(Q),\Phi(Z)) \le C\|\Phi\|_L d_K(Q,Z). \tag{13}$$

Let $Z_1, Z_2 \sim N(0,1)$ be independent standard normal random variables. For simplicity, we use shorthand notations $\sigma_1^2 := \sigma_1^2(\theta_\star) = \frac{N_{\text{ho}}}{N_{\text{obs}}}\dot{\nu}(\theta_\star)^\top\Sigma(\theta_\star)\dot{\nu}(\theta_\star)$ and $\sigma_0^2 := \sigma^2(\theta_\star)$. Observe that $s_{T\nu}(\mathbf{X}_{\text{ho}},\theta_\star;N_{\text{ho}})$ and $N_{\text{obs}}^{1/2}\{\hat{\theta}(\mathbf{X}_{\text{obs}})-\theta_\star)\}$ are jointly independent. Let $Z := \frac{\sigma_0 Z_1 - \sigma_1 Z_2}{(\sigma_0^2+\sigma_1^2)^{1/2}} \sim \mathcal{N}(0,1)$ and $\tilde{Z}_1 := \frac{\sigma_0 Z_1}{(\sigma_0^2+\sigma_1^2)^{1/2}} \sim \mathcal{N}(0,\sigma_0^2/(\sigma_0^2+\sigma_1^2))$ with CDF $F_{\tilde{Z}}$. Let $\tilde{\sigma}_1^2$ be the variance of $\tilde{Z}_1$. By definition, $\|F_{\tilde{Z}_1}\|_{BL} = \|F_{\tilde{Z}_1}\|_\infty + \|F_{\tilde{Z}_1}\|_L = 1 + 1/\sqrt{2\pi\tilde{\sigma}_1^2} < \infty$. It then follows by Lemma 1.1 that,

$$\begin{aligned}
d_K(Q,Z) &= d_K\left(\frac{s_{T\nu}(\mathbf{X}_{\text{ho}},\theta_\star;N_{\text{ho}}) - \dot{\nu}(\theta_\star)^\top N_{\text{ho}}^{1/2}\{\hat{\theta}(\mathbf{X}_{\text{obs}})-\theta_\star\}}{(\sigma^2(\theta_\star)+\sigma_1^2(\theta_\star))^{1/2}}, \frac{\sigma_0 Z_1 - \sigma_1 Z_2}{(\sigma_0^2+\sigma_1^2)^{1/2}}\right)\\
&\le d_K\left(\frac{s_{T\nu}(\mathbf{X}_{\text{ho}},\theta_\star;N_{\text{ho}})}{(\sigma_0^2+\sigma_1^2)^{1/2}}, \frac{\sigma_0 Z_1}{(\sigma_0^2+\sigma_1^2)^{1/2}}\right)\\
&\quad + \|F_{\tilde{Z}_1}\|_{BL} d_{BL}\left(\frac{\dot{\nu}(\theta_\star)^\top N_{\text{ho}}^{1/2}\{\hat{\theta}(\mathbf{X}_{\text{obs}})-\theta_\star\}}{(\sigma_0^2+\sigma_1^2)^{1/2}}, \frac{\sigma_1 Z_2}{(\sigma_0^2+\sigma_1^2)^{1/2}}\right)\\
&= d_K\left(\frac{s_{T\nu}(\mathbf{X}_{\text{ho}},\theta_\star;N_{\text{ho}})}{\sigma_0}, Z_1\right)\\
&\qquad + \|F_{\tilde{Z}_1}\|_{BL} d_{BL}\left(\frac{\dot{\nu}(\theta_\star)^\top N_{\text{ho}}^{1/2}\{\hat{\theta}(\mathbf{X}_{\text{obs}})-\theta_\star\}}{\sigma_1}, Z_2\right).
\end{aligned} \tag{14}$$

By Assumptions A6 and A7 and Assumption (A5-a), the two terms in Eq. (14) are of order $O(N_{\text{obs}}^{-1/2})$. It follows by the definition of $N_{\text{obs}} = \lceil qN \rceil$ that $= O(N_{\text{obs}}^{-1/2}) = O(N^{-1/2})$. Combining Eq. (13) and above facts together, we obtain

$$W_s(\Phi(Q),\Phi(Z)) \le C' d_K(Q,Z) \le O(N^{-1/2}).$$

Hence, we get $W_s(\Phi(Q),\Phi(Z)) = O(N^{-1/2})$. Now combining all parts from **I** to **VIII**, we have for sufficiently large constant $C>0$ and smoothing constant $s \ge 2$

$$W_s(p_{\text{SPC}}(\mathbf{X}),U) \le C\log N/N^{1/2}.$$

Therefore, assuming a smooth model which has large finite moments (large s) allows the rate for Kolmogorov distance to be close to $O(\log N_{\text{obs}}/N_{\text{obs}}^{1/2})$.

By (Bouchitte et al. 2007, Theorem 1.2), for constant $C_s>0$ depending on $s\ge 2$

$$d_K(p_{\text{SPC}}(\mathbf{X}),U) \le C_s W_s^{\frac{s}{1+s}}(p_{\text{SPC}}(\mathbf{X}),U).$$

The proof is immediate by Theorem A.1. □

1.4 Proof of Remark 3.3

Consider $d(\mathbf{X},\theta) = \frac{1}{N}\sum_{i=1}^N (X_i-\theta)^2$. For the Gaussian location model, we have MLE $\hat{\theta}(\mathbf{X}_{\text{obs}}) = \frac{1}{N_{\text{obs}}}\sum_{i=1}^{N_{\text{obs}}} X_{\text{obs},i} \sim \mathcal{N}(\theta_\star,\sigma_\circ^2/N_{\text{obs}})$. Further, the scaled and centered discrepancy measures are

asymptotically normal:

$$\begin{aligned}\sqrt{N_{\text{ho}}}\{d(\mathbf{X}_{\text{pred}},\theta)-\sigma^2\} &= \frac{1}{\sqrt{N_{\text{ho}}}}\left\{\sum_{i=1}^{N_{\text{ho}}}(X_{\text{pred},i}-\theta)^2-\sigma^2 N_{\text{ho}}\right\} \\ &\sim \frac{\chi^2_{N_{\text{ho}}}-N_{\text{ho}}}{\sqrt{N_{\text{ho}}}}\cdot\sigma^2 \xrightarrow{\mathcal{D}} \mathcal{N}(0,\sigma^4)\end{aligned} \tag{15}$$

and

$$\begin{aligned}\sqrt{N_{\text{ho}}}\{d(\mathbf{X}_{\text{ho}},\theta_\star)-\sigma_\circ^2\} &= \frac{1}{\sqrt{N_{\text{ho}}}}\left\{\sum_{i=1}^{N_{\text{ho}}}(X_{\text{ho},i}-\theta_\star)^2-\sigma_\circ^2 N_{\text{ho}}\right\} \\ &\sim \frac{\chi^2_{N_{\text{ho}}}-N_{\text{ho}}}{\sqrt{N_{\text{ho}}}}\cdot\sigma_\circ^2 \xrightarrow{\mathcal{D}} \mathcal{N}(0,\sigma_\circ^4).\end{aligned} \tag{16}$$

By definition of single SPC p-value

$$\begin{aligned}p_{\text{SPC}}(\mathbf{X}) &= 1-\int \mathbb{P}\left[d(\mathbf{X}_{\text{pred}},\theta)\geq d(\mathbf{X}_{\text{ho}},\theta)\mid\theta\right]\Pi(\mathrm{d}\theta\mid\mathbf{X}_{\text{obs}}) \\ &= 1-\int \mathbb{P}\left[\sqrt{N_{\text{ho}}}\,d(\mathbf{X}_{\text{pred}},\theta)\geq \sqrt{N_{\text{ho}}}\,d(\mathbf{X}_{\text{ho}},\theta)\mid\theta\right]\widehat{\Pi}(\mathrm{d}\theta\mid\mathbf{X}_{\text{obs}})+o_P(1).\end{aligned} \tag{17}$$

Observe that $\sqrt{N_{\text{ho}}}\,d(\mathbf{X}_{\text{ho}},\theta)$ is not centralized. We write

$$\sqrt{N_{\text{ho}}}\,d(\mathbf{X}_{\text{ho}},\theta) = \sqrt{N_{\text{ho}}}\,d(\mathbf{X}_{\text{ho}},\theta_\star)+\sqrt{N_{\text{ho}}}(\theta-\theta_\star)^2+2(\theta-\theta_\star)\frac{1}{\sqrt{N_{\text{ho}}}}\sum_{i=1}^{N_{\text{ho}}}(X_{\text{ho},i}-\theta_\star)^2. \tag{18}$$

The first term in Eq. (18) follows a scaled $\chi^2_{N_{\text{ho}}}$ distribution. For the second term, we have

$$\begin{aligned}\sqrt{N_{\text{ho}}}(\theta-\theta_\star)^2 &= \sqrt{N_{\text{ho}}}(\theta-\hat\theta(\mathbf{X}_{\text{obs}}))^2+\sqrt{N_{\text{ho}}}(\hat\theta(\mathbf{X}_{\text{obs}})-\theta_\star)^2 \\ &\quad+2\sqrt{N_{\text{ho}}}(\theta-\hat\theta(\mathbf{X}_{\text{obs}}))(\hat\theta(\mathbf{X}_{\text{obs}})-\theta_\star) \\ &\sim \frac{N_{\text{ho}}}{N_{\text{obs}}}\frac{1}{\sqrt{N_{\text{ho}}}}(\sigma^2\chi^2_1+\sigma_\circ^2\chi^2_1+2\sigma\sigma_\circ\chi^2_1),\end{aligned} \tag{19}$$

which converges to 0 in probability as $N\to\infty$. Similarly for the last term in Eq. (18), we have

$$\begin{aligned}2(\theta-\theta_\star)\frac{1}{\sqrt{N_{\text{ho}}}}\sum_{i=1}^{N_{\text{ho}}}(X_{\text{ho},i}-\theta_\star) &= 2\Bigg\{\sum_{i=1}^{N_{\text{ho}}}\left(\theta-\hat\theta(\mathbf{X}_{\text{obs}})\right)(X_{\text{ho},i}-\theta_\star) \\ &\qquad+\sum_{i=1}^{N_{\text{ho}}}\left(\hat\theta(\mathbf{X}_{\text{obs}})-\theta_\star\right)(X_{\text{ho},i}-\theta_\star)\Bigg\} \\ &= \frac{2\sigma_\circ}{\sqrt{N_{\text{ho}}N_{\text{obs}}}}\left(\sigma\chi^2_{N_{\text{ho}}}+\sigma_\circ\chi^2_{N_{\text{ho}}}\right) \\ &= o_P(1).\end{aligned} \tag{20}$$

Substituting Eqs. (19) and (20) into Eq. (18) yields

$$\sqrt{N_{\text{ho}}}\,d(\mathbf{X}_{\text{ho}},\theta) = \sqrt{N_{\text{ho}}}\,d(\mathbf{X}_{\text{ho}},\theta_\star)+o_P(1).$$

Let Z_1, Z_2 be independent standard normal random variables and $\mathbb{E}_\theta[\cdot]$ denote the expectation with respect to the posterior distribution. By the DCT and Eqs. (15) and (16), we can rewrite the p-values in Eq. (17) as

$$\begin{aligned}
p_{\text{SPC}}(\mathbf{X}) &= 1 - \mathbb{E}_\theta\left[\mathbb{P}\left[\sqrt{N_{\text{ho}}}\, d(\mathbf{X}_{\text{pred}}, \theta) \geq \sqrt{N_{\text{ho}}}\, d(\mathbf{X}_{\text{ho}}, \theta) \mid \theta\right]\right] + o_P(1) \\
&= 1 - \mathbb{E}_\theta\Bigg\{\mathbb{P}\Bigg[\frac{\sqrt{N_{\text{ho}}}\{d(\mathbf{X}_{\text{pred}}, \theta) - \sigma^2\}}{\sigma^2} \geq \frac{\sqrt{N_{\text{ho}}}\{d(\mathbf{X}_{\text{ho}}, \theta) - \sigma_\circ^2\}}{\sigma_\circ^2}\frac{\sigma_\circ^2}{\sigma^2} \\
&\qquad\qquad + \frac{\sqrt{N_{\text{ho}}}\,(\sigma_\circ^2 - \sigma^2)}{\sigma^2} \mid \theta\Bigg]\Bigg\} + o_P(1) \\
&= 1 - \mathbb{P}\left[Z_1 \geq \kappa^2 Z_2 + \sqrt{N_{\text{ho}}}\,(\kappa^2 - 1)\right] + o_P(1) \\
&= \Phi\left(\kappa^2 Z_2 + \sqrt{N_{\text{ho}}}\,(\kappa^2 - 1)\right) + o_P(1).
\end{aligned}$$

Letting $\kappa = 1$ we have $p_{\text{SPC}}(\mathbf{X}) = \Phi(Z_2) + o_P(1) \xrightarrow{\mathcal{D}} \mathsf{Unif}$ and therefore

$$\mathbb{P}\left[p_{\text{SPC}}(\mathbf{X}) < \frac{\alpha}{2}\right] = \frac{\alpha}{2}.$$

For $\kappa \neq 1$, followed by a similar argument in the proof of Theorem A.1, we have

$$\begin{aligned}
\mathbb{P}\left[p_{\text{SPC}}(\mathbf{X}) < \frac{\alpha}{2}\right] &= \mathbb{P}\left[\Phi\left(\kappa^2 Z_2 + \sqrt{N_{\text{ho}}}\,(\kappa^2 - 1)\right) + o_P(1) < \frac{\alpha}{2}\right] \\
&= \mathbb{P}\left[\frac{z_{\frac{\alpha}{2}} - \sqrt{N_{\text{ho}}}\,(\kappa^2 - 1)}{\kappa^2} < Z_2 + o_P(1)\right] \\
&= 1 - \Phi\left(\frac{z_{\frac{\alpha}{2}} - \sqrt{N_{\text{ho}}}\,(\kappa^2 - 1) - o_P(1)}{\kappa^2}\right) \\
&\xrightarrow{P} 1,
\end{aligned}$$

where the last inequality follows by taking $N_{\text{ho}} \to \infty$ and applying DCT. Similarly, we can derive for $\kappa \neq 1$

$$\mathbb{P}\left[p_{\text{SPC}}(\mathbf{X}) > 1 - \frac{\alpha}{2}\right] = \Phi\left(\frac{z_{\frac{\alpha}{2}} - \sqrt{N_{\text{ho}}}\,(\kappa^2 - 1) - o_P(1)}{\kappa^2}\right) \xrightarrow{P} 0.$$

1.5 Proof of Theorem 3.4

We first introduce some notation. Let $\hat{\mathbb{P}}_n := n^{-1}\sum_{i=1}^n \delta_{X_i}$ be the empirical measure of $X_1, \ldots, X_n$. The scaled empirical process is defined as $\mathbb{G}_n^P := \sqrt{n}(\hat{\mathbb{P}}_n - P)$. Given a collection of functions, the empirical process $\hat{\mathbb{P}}_n$ induces a map from $\mathscr{F}$ to $\mathbb{R}$ defined as $f \mapsto \hat{\mathbb{P}}_n f := n^{-1/2}\sum_{i=1}^n f(X_i)$ for some $f \in \mathscr{F}$. Consider the collection of indicator functions $\mathscr{F}_{\text{ind}} := \{\mathbb{1}_{(-\infty, x]}, x \in \mathbb{R}\}$. The Kolmogorov distance can be rewritten as

$$d_K(\hat{\mathbb{P}}_n, P) = \sup_{f \in \mathscr{F}_{\text{ind}}} (\hat{\mathbb{P}}_n - P)f.$$

Recall that divided SPCs generate p-values by applying the Kolmogorov-Smirnov test to k single SPC p-values obtained from k disjoint subsets. We require a uniform convergence on Kolmogorov distance in the proof of Theorem 3.4, which we present in the following lemma.

Lemma 1.6. *(Uniform convergence on Kolmogorov distance) If $X_1, X_2, \ldots$ i.i.d. $\sim P$ a continuous distribution, then the empirical distribution uniformly converges to the true distribution:*

$$\sup_P d_K\left(\sup_{f\in\mathscr{F}_{ind}} \mathbb{G}_n^P f, K\right) \to 0,$$

where $K \sim F_{\mathrm{KS}}(\cdot)$, the Kolomogorov distribution with CDF $F_{\mathrm{KS}}(t) := 1 - 2\sum_{i=1}^{\infty}(-1)^{i-1}e^{-2i^2t}$.

Proof. Consider the class of indication functions $\mathscr{F}_{\mathrm{ind}} = \{\mathbb{1}_{(-\infty,x]}, x \in \mathbb{R}\}$. It's easy to show that $\mathscr{F}_{\mathrm{ind}}$ has Vapnik-Chervonenkis (VC) dimension $\nu(\mathscr{F}_{\mathrm{ind}}) = 2$. Following the discussions of Ledoux & Talagrand (1991, Theorem 14.13), such VC class $\mathscr{F}_{\mathrm{ind}}$ belongs to uniform Donsker classes. It then follows from Sheehy & Wellner (1992, Definition 2.3 and Theorem 2.1) that an equivalent definition of uniform Donsker class is, for any $t > 0$

$$\lim_{n\to\infty}\sup_P \mathbb{P}^*\left\{\sup_{f\in\mathscr{F}_{\mathrm{ind}}} (\mathbb{G}_n^P - \mathbb{G}^{(n)})(f) > t\right\} = 0,$$

where $\mathbb{G}^{(n)}$ is a sequence of coherent P-Brownian bridge processes. The conclusion immediately follows by the fact that the supremum of Brownian bridge follows the Kolmogorov distribution (Boukai 1990). □

Proof of Theorem 3.4. Suppose $k = N^\beta$ for $0 < \beta < \frac{\gamma}{\gamma+1}$. By definition we have $N_k = \lceil \frac{N}{k} \rceil = \lceil N^{1-\beta} \rceil$. It follows from Assumption A1 that for constant $C' > C$

$$d_K(\mathbb{P}_k, \mathsf{Unif}) < CN_k^{-\gamma/2} < C'N^{(\beta-1)\frac{\gamma}{2}}.$$

Consider the Kolmogorov distance between $\hat{\mathbb{P}}_k$ and Unif and fix some $t > 0$. It follows from the triangle inequality that

$$\begin{aligned}
\mathbb{P}\left[k^{1/2}d_K(\hat{\mathbb{P}}_k, \mathsf{Unif}) > t\right] &\le \mathbb{P}\left[k^{1/2}d_K(\mathbb{P}_k, \mathsf{Unif}) + k^{1/2}d_K(\hat{\mathbb{P}}_k, \mathbb{P}_k) > t\right] \\
&\le \mathbb{P}\left[C'N^{\beta/2}N^{(\beta-1)\gamma/2} + k^{1/2}d_K(\hat{\mathbb{P}}_k, \mathbb{P}_k) > t\right] \\
&= \mathbb{P}\left[k^{1/2}d_K(\hat{\mathbb{P}}_k, \mathbb{P}_k) > s - C'N^{\frac{1}{2}(\beta(1+\gamma)-\gamma)}\right]. \qquad (21)
\end{aligned}$$

Note that $\beta < \frac{\gamma}{\gamma+1}$ implies $\frac{1}{2}(\beta(1+\gamma) - \gamma) < 0$. By Lemma 1.6 and taking $N \to \infty$, we have the last line in Eq. (21) converges to $1 - F_{\mathrm{KS}}(t)$ in the Kolmogorov metric. On the other hand, by applying the triangle inequality, we have

$$\begin{aligned}
\mathbb{P}\left[k^{1/2}d_K(\hat{\mathbb{P}}_k, \mathsf{Unif}) > t\right] &\ge \mathbb{P}\left[k^{1/2}d_K(\hat{\mathbb{P}}_k, \mathbb{P}_k) - k^{1/2}d_K(\mathbb{P}_k, \mathsf{Unif}) > t\right] \\
&\ge \mathbb{P}\left[k^{1/2}d_K(\hat{\mathbb{P}}_k, \mathbb{P}_k) > t + C'N^{\frac{1}{2}(\beta(1+\gamma)-\gamma)}\right]. \qquad (22)
\end{aligned}$$

Combining Eqs. (21) and (22) and Lemma 1.6 yields

$$\left|\mathbb{P}\left[k^{1/2}d_K(\hat{\mathbb{P}}_k, \mathsf{Unif}) > t\right] - (1 - F_{\mathrm{KS}}(t))\right| \xrightarrow{k\to\infty} 0.$$

□

1.6 Proof of Theorem 3.5

Proof. Fix any $t > 0$. By assumption, there exists an $\epsilon > 0$ and $\bar{n}(\epsilon)$ such that for any $n_k > \bar{n}(\epsilon)$, $d_K(\mathbb{P}_k, \mathsf{Unif}) > \epsilon$. Together with the triangle inequality, we have

$$\begin{aligned}\mathbb{P}\left[\sqrt{k}\, d_K(\hat{\mathbb{P}}_k, \mathsf{Unif}) > t\right] &\geq \mathbb{P}\left[\sqrt{k}\, d_K(\mathbb{P}_k, \mathsf{Unif}) - \sqrt{k}\, d_K(\hat{\mathbb{P}}_k, \mathbb{P}_k) > t\right] \\ &\geq \mathbb{P}\left[\sqrt{k}\, d_K(\hat{\mathbb{P}}_k, \mathbb{P}_k) < \sqrt{k}\,\epsilon - K_\alpha\right]. \end{aligned} \tag{23}$$

It follows from Lemma 1.6 that

$$\liminf_{k\to\infty} \sup_P \mathbb{P}\left[\sqrt{k}\, d_K(\hat{\mathbb{P}}_k, P) < \sqrt{k}\,\epsilon - t\right] = 1. \tag{24}$$

Combining Eqs. (23) and (24) yields

$$\liminf_{k\to\infty} \mathbb{P}\left[\sqrt{k}\, d_K\left(\hat{\mathbb{P}}_k, \mathsf{Unif}\right) > t\right] = 1.$$

Since $\liminf \leq \lim$ and probabilities are upper bounded by 1, we have

$$\lim_{k\to\infty} \mathbb{P}\left[\sqrt{k}\, d_K\left(\hat{\mathbb{P}}_k, \mathsf{Unif}\right) > t\right] = 1.$$

□

2 Additional figures and tables

We present additional figures and tables for the previous simulation study and experiments in this section. In Section 2.1, we provide a simulation study of all candidate checks under Gaussian location model. The Poisson model simulation study are displayed in Section 2.2. For additional figures of Gaussian hierarchical simulation study, see Section 2.3.

2.1 Gaussian location model

In this section, we investigate all the candidate checks with a Gaussian location model. Consider

$$\begin{aligned} X &\sim \mathcal{N}(\theta, 1) \\ \theta &\sim \mathcal{N}(0, 10000). \end{aligned} \tag{25}$$

We design two generative models to indicate two extreme cases of model mismatches. The assumed model in (25) is well-specified when the data is generated from $P_o : X \sim \mathcal{N}(0, 1)$ and misspecified if we generate from $P_o : X \sim \mathcal{N}(0, 15^2)$. We implement all checking methods with the following statistics: (i) empirical mean $T_N(\mathbf{x}_N) := \frac{1}{N}\sum_{i=1}^N x_i$, (ii) 2nd moment $:= \frac{1}{N}\sum_{i=1}^N x_i^2$, (iii) 75th quantile $:= \arg\min_T |\frac{1}{N}\sum_{i=1}^N \mathbb{1}_{\{x_i \leq T\}} - 0.75|$ and (iv) mean squared error (MSE) $:= \frac{1}{N}\sum_{i=1}^N (x_i - \mathbb{E}[\mathbf{X}_i \mid \theta])^2$. All statistics are asymptotically normal distributed as required by Assumption A6.

Single SPCs Fig. B.9 verifies the results in Theorem 3.1 that when $\nu_\circ = \nu(\theta_\star)$, with mean statistic, the asymptotic power of single SPC depends on $\rho^2 = \frac{q\sigma_\circ^2+(1-q)\sigma_\circ^2/N_{\text{ho}}}{q\sigma(\theta_\star)^2+(1-q)\sigma(\theta_\star)^2/N_{\text{ho}}} = \sigma_\circ^2/\sigma(\theta_\star)^2$. The asymptotic power is independent of q in this case. For 2nd moment statisitic, single SPCs with all proportions successfully capture the major mismatches and q does not affect the performance when $\nu_\circ \neq \nu(\theta_\star)$.

However, for small sample regime, a small split proportion such as $q = 0.1$ is not recommended due to a limited number of split observed data in computing posterior distributions. In Fig. B.9c, when $N = 50$, the single SPC with $q = 0.1$ computes the posterior distribution with only $N_{\text{obs}} = 5$ samples, which tends to produce overconfident p-values. Fig. B.10 shows similar results for 75th quantile and MSE statistic with different proportions of single SPC.

Divided SPCs The choices of split proportion q are of significance to the performance of divided SPCs in a small-data regime, and takes the same role with large datasets as in single SPC does. Given a sample of size $N = 5000$, Fig. B.11c shows that the divided SPC with $q = 0.1, 0.3$ blows up the Type I error. The reason is that the data used to compute the posterior distribution only contains $N_{\text{obs}} = 7$ observations for $q = 0.1$ and $N_{\text{obs}} = 23$ for $q = 0.3$. Hence, we recommend choosing moderate values for proportions such as $q \in \{0.5, 0.7\}$ for divided SPC when the data set is not large.

Effective number of folds k We show different choices of k with various statistics in Fig. B.12. In Fig. B.12a, divided SPC with 2nd moment statistic and rate $\beta > 0.5$ results in large Type I errors, which coincides with Theorem 3.4. On the other hand, choosing rates too small may produce inefficient powers in small-data scenario, as in Fig. B.12d.

Comparison of PPC and SPCs In Fig. B.13, divided SPC outperforms all methods in power with mean statistic. PPC produces 0 power, while single 0.5-SPC has power stabilized at a value not equal to 1. All candidate methods succeed to achieve power 1 with the 75th quantile. For the test size comparison, all methods have the test size under control.

2.2 Poisson model

We consider the conjugate Poisson model $P_\theta = \mathsf{Poiss}(\theta)$ and $\theta \sim= \mathsf{Gamma}(0.1, 0.2)$, a gamma distribution parameterized by the shape and rate. We investigate the behavior of the predictive checks for two data-generating distributions, the well-specified model $P_\circ = \mathsf{Poiss}(2)$ and the misspecified model $P_\circ = \mathsf{NegBin}(2, 0.01)$, a negative binomial distribution parameterized by the mean and dispersion. We implement all methods using four statistics: (i) empirical mean $T_N(\mathbf{x}) = \frac{1}{N}\sum_{i=1}^N x_i$, (ii) 2nd moment $T_N(\mathbf{x}) = \frac{1}{N}\sum_{i=1}^N x_i^2$, (iii) 3rd moment $T_N(\mathbf{x}) = \frac{1}{N}\sum_{i=1}^N x_i^3$ and (iv) mean squared error (MSE) $T_N(\mathbf{x}, \theta) = \frac{1}{N}\sum_{i=1}^N (x_i - \mathbb{E}[\mathbf{X}_i \mid \theta])^2$. Similar results are also obtained in the case of the Gaussian location model, which we present in 2.1.

Single SPCs. Figure B.1 confirms that, as shown in Theorem 3.1, the choice of q controls the asymptotic behavior of single SPCs when $\nu_\circ = \nu(\theta_\star)$ and has no impact when $\nu_\circ \neq \nu(\theta_\star)$. For the small sample size regime, a moderate split proportion such as $q = 0.5$ is preferable to an extreme one. As shown in Fig. B.1a, single SPC with $q = 0.9$ produces the smallest power when $N = 1000$. We also note that a small split proportion such as $q = 0.1$ results in a slightly larger Type I error. As in Fig. B.2, small split proportions are not recommended for single SPCs due to

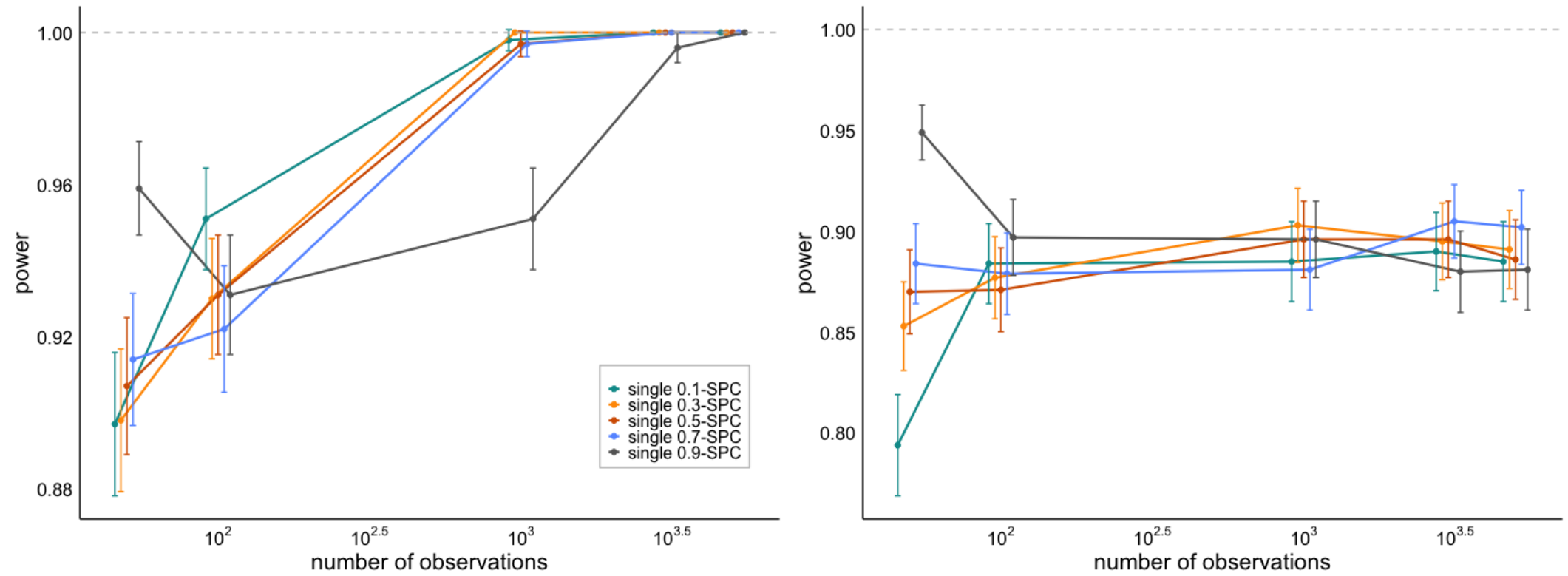

(a) 2nd moment (b) mean

Figure B.1: Poisson Model: Power plots for single SPC with different split proportions $q \in \{0.1, 0.3, 0.5, 0.7, 0.9\}$ under conjugate Poisson model. The data is generated from negative binominal distribution $\mathsf{NegBin}(2,\ 0.01)$ and thus Poisson model is misspecified. The vertical bars represent the 95% confidence intervals for the power estimates. The dashed horizontal line in the plots is at 1 indicating the optimal power of a test.

a limited number of split observed data in computing posterior distributions. On the other hand, Figure B.1b empirically verifies the limitations of single SPCs documented in Theorem 3.1: for all single SPCs using the mean statistic and where mild-to-moderate misspecification occurs, the power stays around 0.9 and has no tendency to increase as the sample size grows. We also present results for 3rd moment and MSE, which correspond to moderate misspecification case. The power plot with MSE in Fig. B.14d shows another limitation of single SPC that with small sample, a large split proportion q may result in a slow rate of convergence in power.

Divided SPCs. For divided SPCs, first we fix $k = N^{0.49}$ and vary q. Fig. B.4a shows that the divided SPC has asymptotic power 1 and since $\rho = \sigma_\circ/\sigma(\theta_\star)$, the split proportion makes no difference in the power performance of divided SPC given a large sample size. However, when dealing with small-to-moderate sample sizes, one needs to be careful with the choice of split proportion for divided SPC. Fig. B.4 shows that with $\beta > 1/2$, the test size is not well controlled, which is in agreement with conclusion of Theorem 3.4 that only guarantees the divided SPC is asymptotically well-calibrated when when $\beta \in (0, 1/2)$. We also present the results for 2nd and 3rd moments. As is shown in Figs. B.5 and B.15, the divided SPC with both $q = 0.1$ and $q = 0.9$ fail to control the test size well even when $N = 5000$. Therefore, even though the power increases with q, in the small-to-moderate sample-size regime the best choice for the proportion is $q = 0.5$. Divided SPC with different choices of k using 2nd moment and mean are shown in Fig. B.16. Both demonstrate similar results as in Fig. B.4.

Effect of number of folds k. To investigate the effect of different scalings of k, we fix the split proportion $q = 0.5$ and consider $k = N^\beta$ for different choices of β. Fig. B.5 shows that with $\beta > 1/2$, the test size is not well controlled, which is in agreement with conclusion of Theorem 3.4 that only guarantees the divided SPC is asymptotically well-calibrated when when $\beta \in (0, 1/2)$.

On the other hand, choosing small β results in an insufficient number of folds and thus degrades the power of the KS test. We find that $\beta \approx 1/2$ provides the best power while $\beta \approx 0.4$ has smaller

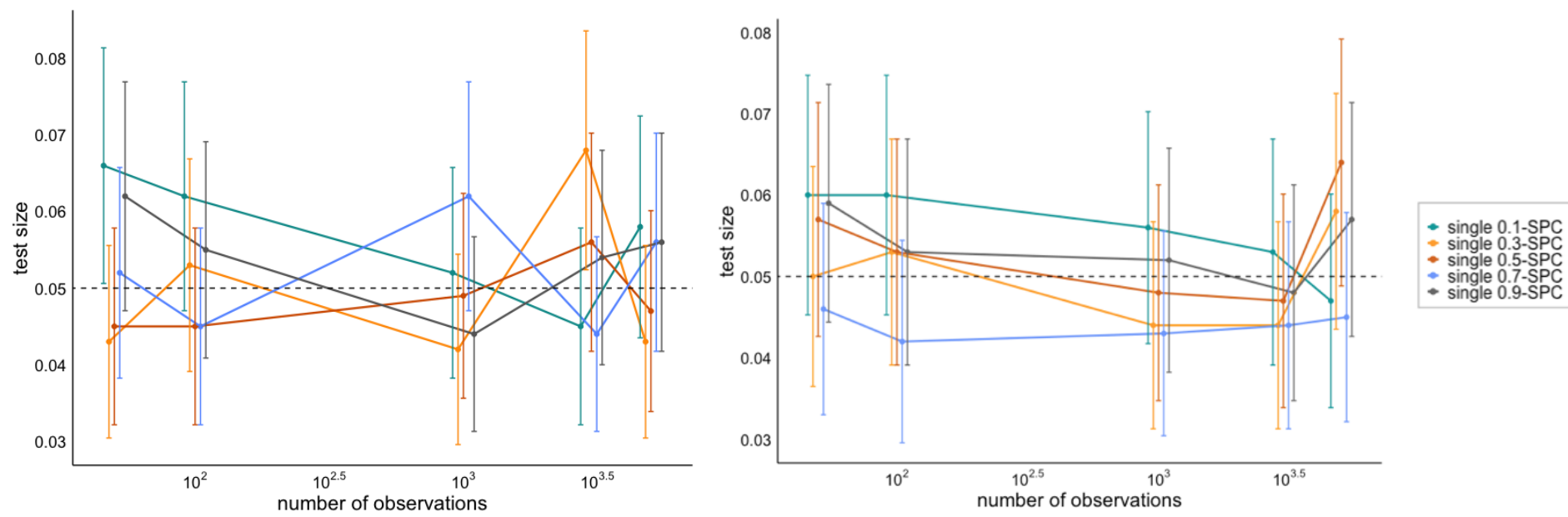

(a) mean (b) 2nd moment

Figure B.2: Poisson Model: Test size plots for single SPC with different split proportions $q \in \{0.1, 0.3, 0.5, 0.7, 0.9\}$, under conjugate Poisson model. The data was generated from $\mathsf{Poiss}(2)$ to create a well-specified model. The vertical bars represent the 95% confidence intervals for the test size estimates. Each test size is estimated with 1000 single SPC p-values that are computed with 1000 datasets of size $N \in \{50, 100, 1000, 3000, 5000\}$. The dashed horizontal line in the test size plots indicates the significance level $\alpha = 0.05$.

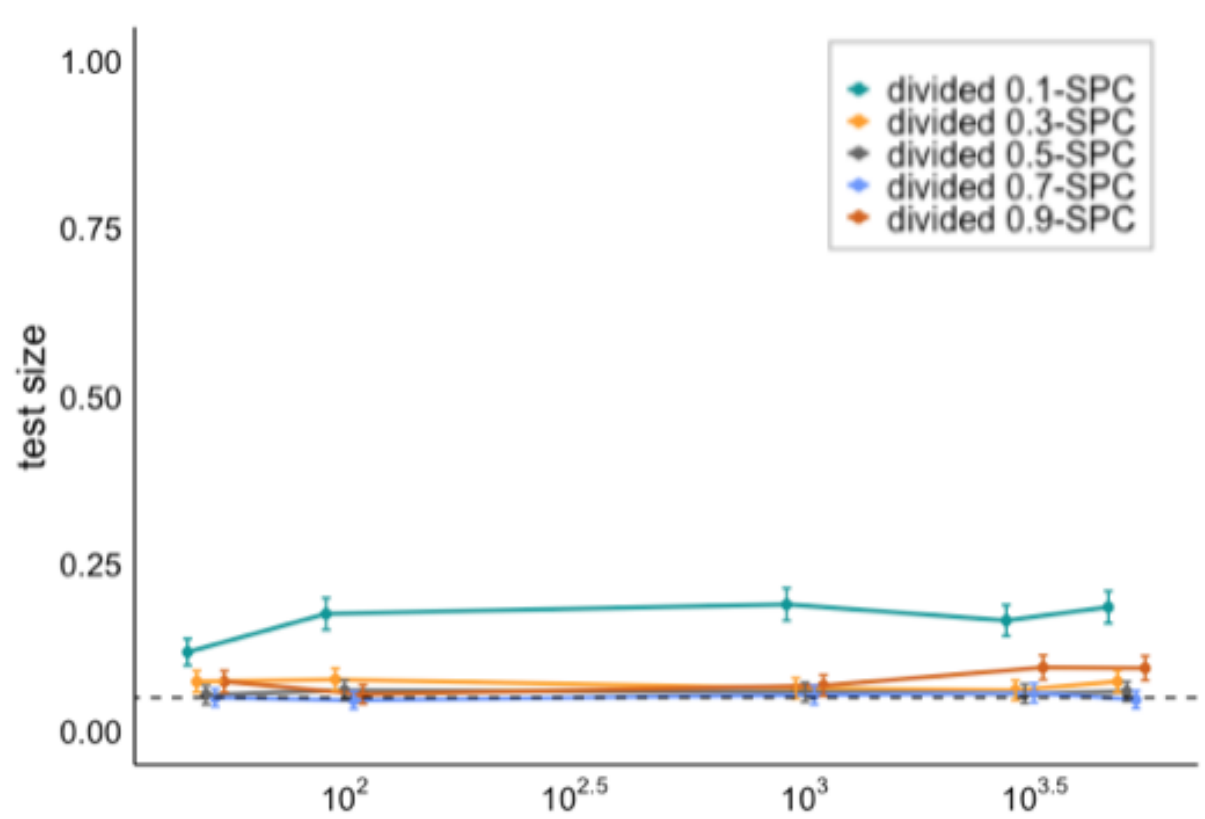

Figure B.3: Poisson Model: Test size for divided SPC with mean statistic, $k = N^{0.49}$, and varying $q \in \{0.1, 0.3, 0.5, 0.7, 0.9\}$, under the conjugate Poisson model. The data was generated from $\mathsf{Poiss}(2)$ to create a well-specified model. See Fig. B.1 for further explanation and experimental details.

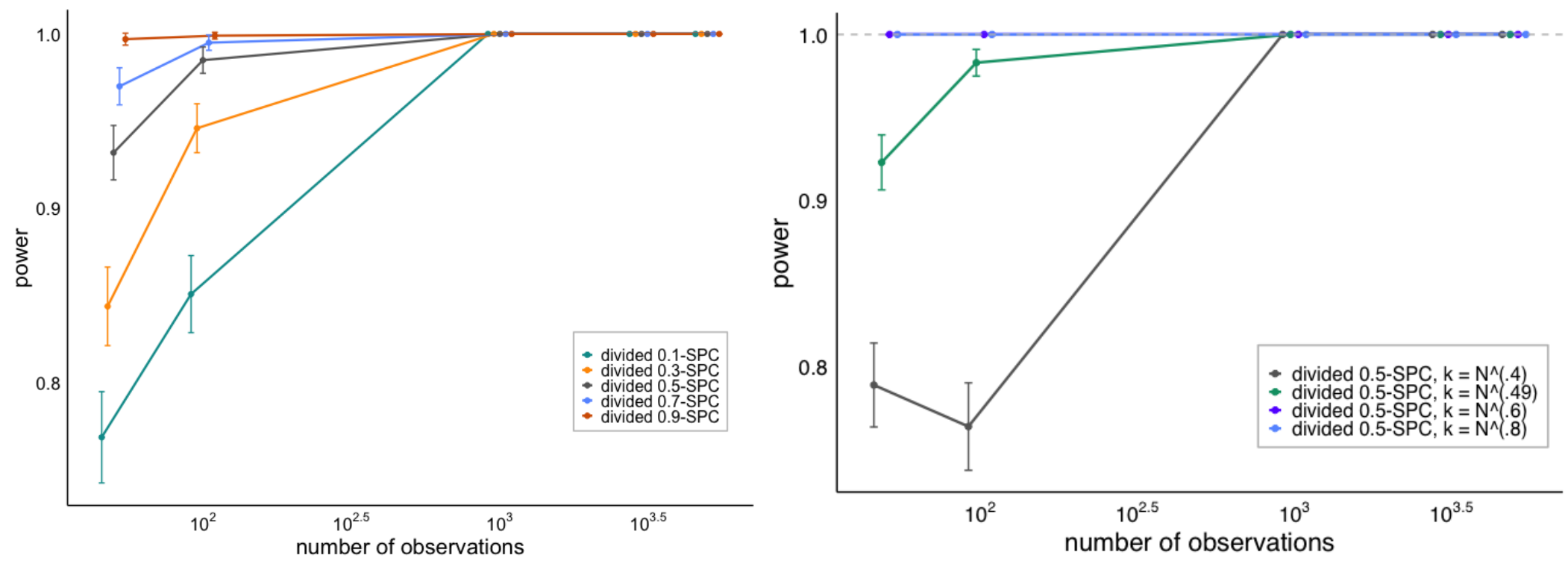

Figure B.4: Poisson Model: we fit a Poisson model to the data generated from negative binominal distribution NegBin(2,0.01). **Left:** Power for divided SPC with mean statistic, $k = N^{0.49}$, and varying $q \in \{0.1, 0.3, 0.5, 0.7, 0.9\}$, under the conjugate Poisson model. **Right:**Power for divided SPC with 3rd moment statistic, $q = 0.5$, and varying the number of folds $k \in \{N^{0.4}, N^{0.49}, N^{0.6}, N^{0.8}\}$. See Fig. B.1 for further explanation and experimental details.

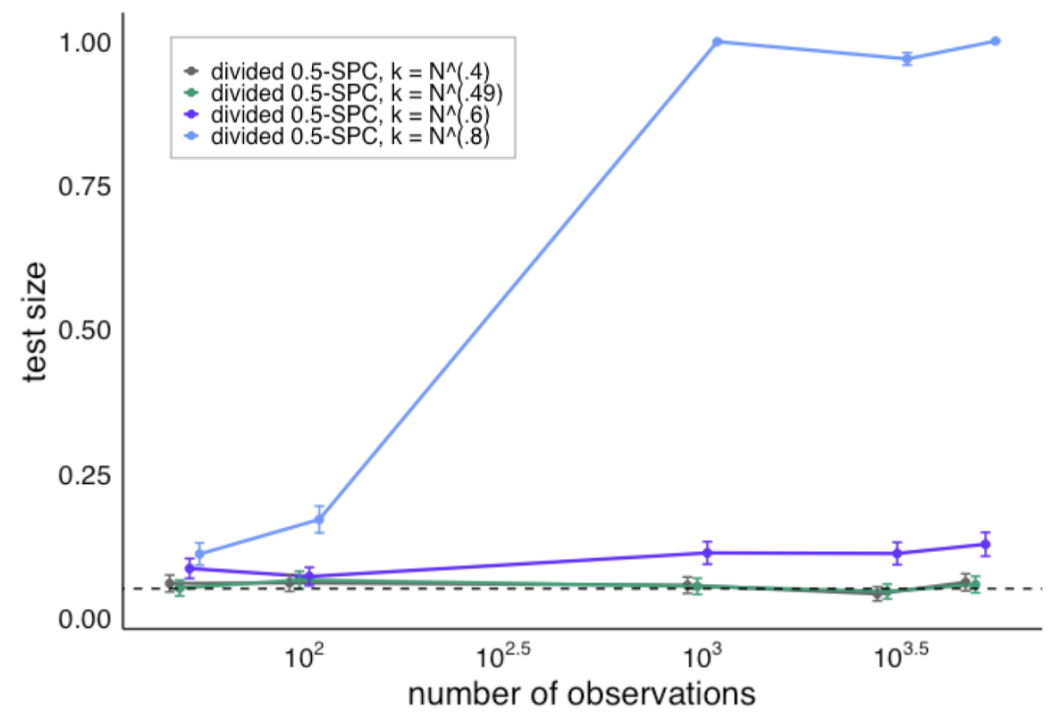

Figure B.5: Poisson Model: we fit a Poisson model to the data generated from Poisson distribution Poiss(2) to compare different checking methods under a well-specified model. We plot test size for divided SPC with mean statistic, $k = N^{0.49}$, and varying $q \in \{0.1, 0.3, 0.5, 0.7, 0.9\}$.

power. Results for other statistics are presented in Fig. B.16 in Supplementary Materials.

Effect of the degree of misspecification. To investigate how the degree of misspecification affects each predictive check, we use the data-generating distributions $P_\circ = \mathsf{NegBin}(2, \tau)$ and $P_\circ = \mathsf{Binom}(30, p)$ to create over- and under-dispersed data and compare the asymptotic power of each method given different types of misspecification. Using a mean statistic in this case yields $\nu_\circ = \nu(\theta_\star)$, so the degree of misspecification is effectively characterized by $\rho = \sigma_\circ/\sigma(\theta_\star)$ from Theorem 3.1. For the negative binomial model $\rho^2 = 1+2/\tau$ and for the binomial model $\rho^2 = 1-p$. Hence, varying $\tau \in \{0.01, 0.1, 0.5\}$ for the negative binomial model and $p \in \{0.1, 0.5, 0.8\}$ for the binomial model leads to the subtle-to-moderate misspecification scenarios $\rho^2 \in \{0.2, 0.5, 0.9, 5, 21, 201\}$. Fig. B.6a shows that, as expected, the power of single SPC stabilizes near zero for $\rho^2 < 1$ but approaches one when $\rho^2 \gg 1$. The power of divided SPC, on the other hand, reaches one for all cases except the nearly well-specified one of $\rho^2 = 0.9$. The power of PPC is zero in all cases. Switching to the 2nd moment statistic, $\nu_\circ \neq \nu(\theta_\star)$ so there is substantial misspecification. As expected, Fig. B.6b shows that PPC, single and divided SPC all succeed to capture such substantial

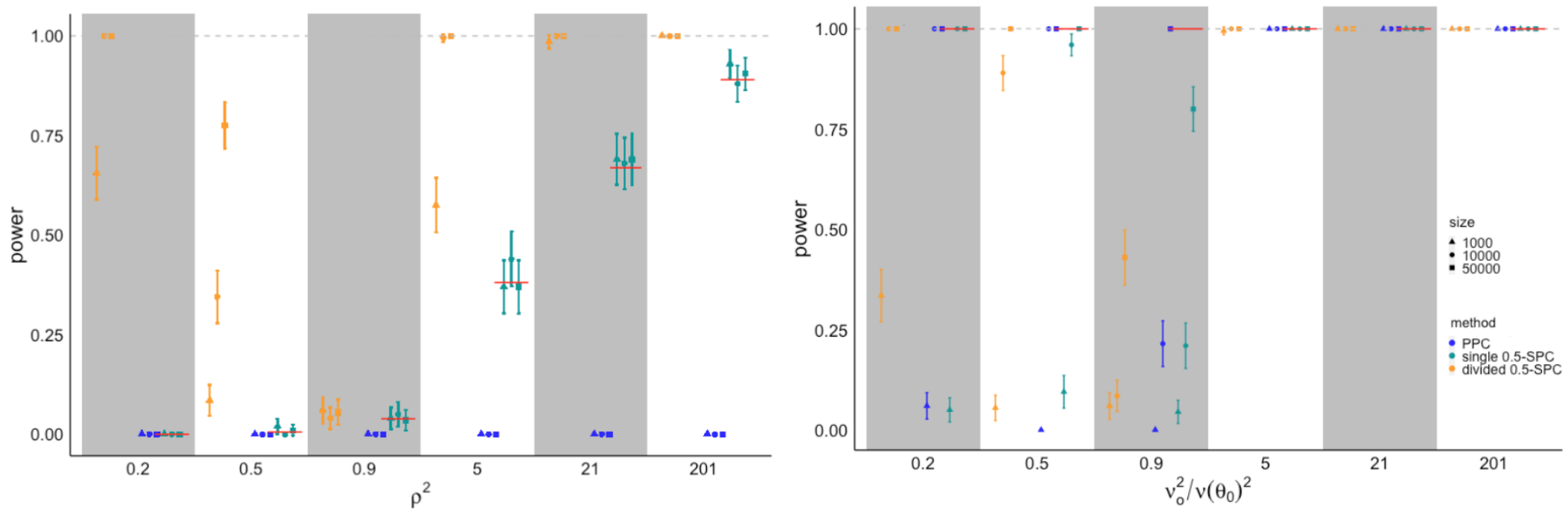

(a) mean (b) 2nd moment

Figure B.6: Poisson Model: The power plots of candidate checks given different levels of misspecification with conjugate Poisson model. For $\rho^2 = 0.2, 0.5, 0.9$, the data was generated from $\mathsf{Binom}(p)$ with $p = 0.8, 0.5, 0.1$ respectively. For $\rho^2 = 5, 21, 201$, the data was generated from $\mathsf{NegBin}(2, \tau)$ with $\tau = 0.5, 0.1, 0.01$ respectively. The red solid lines indicate theoretical asymptotic power of single SPC computed from Theorem 3.1. All SPCs choose proportion as $q = 0.5$ and for divided SPCs, the number of splits is set as $k = N^{0.49}$.

misspecification with power 1 given large datasets. On the whole, these results suggest that, when used together, single and divided SPC are superior to using PPC.

2.3 Additional figures for Gaussian Hierarchical simulation study of Section 5

We show results of all methods given fixed number of groups $I = 20$ and increasing number of individuals per group J in Fig. B.17. For group-level mismatches, Fig. B.17a shows that increasing number of observations per group may not help in detecting such misspecification even with proper statistics. However, with lower-level misspecified model, we see in Fig. B.17b that power of PPC and SPCs with within-splits tend to increase as the number of observations per group increases. A large sample size for each group also provides more information to checking methods when the model is misspecified in both levels as shown in Fig. B.17c.

2.4 Real-data experiment: light speed data

The light speed data is discussed in both Gelman et al. (2013) and Robins et al. (2000). The data were collected by Simon Newcomb (1882), who measured the time that light takes to travel 7442 meters at sea level. The observations are recorded as deviations from 24800 nanoseconds and $N = 66$. We consider a normal model $P_\theta = \mathcal{N}(\mu, \sigma^2)$ with an improper prior density $\pi_0(\mu, \sigma^2) = \sigma^{-2}$. A comparison of the data distribution and the posterior predictive distribution is displayed in Section 2.4, which shows that the model does not capture the heavy left tail of the data and that the 95% posterior credible interval does not contain the true value. We consider five statistics: mean, standard deviation, MSE, 5th and 95th quantiles. We use $q = 0.5$ to ensure the statistics (particularly the quantiles) can be reasonably estimated. Two-sided p-values computed for each method and statistic are given in Table 1. Both the PPC and single SPC yield small p-values for the left quantile. The single SPC yields a small p-value for the standard deviation and MSE

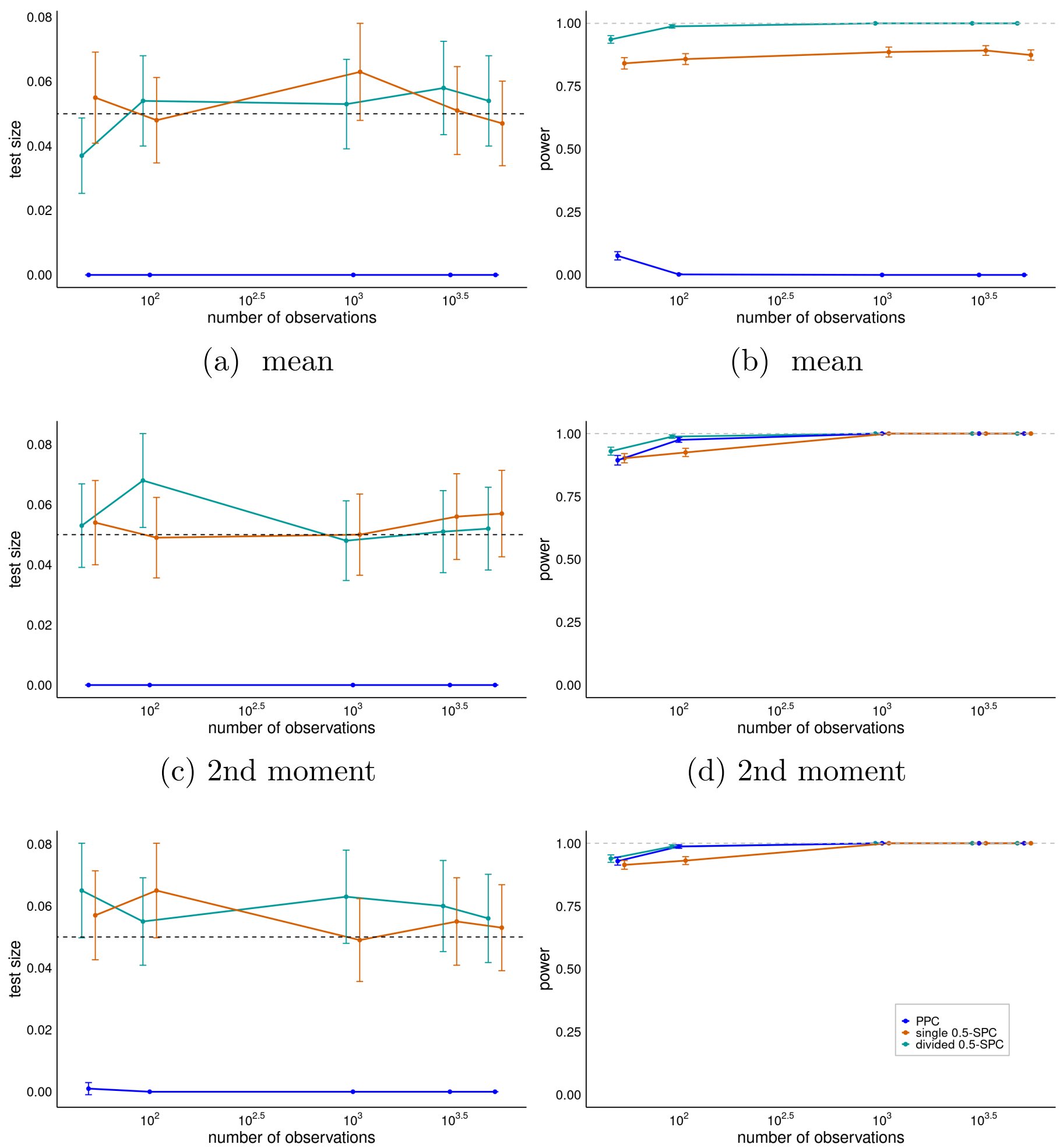

(a) mean (b) mean

(c) 2nd moment (d) 2nd moment

(e) 3rd moment (f) 3rd moment

Figure B.7: Poisson Model: Test size and power plots in log scale for different checking methods and statistics under the conjugate Poisson model. We display experiments for $N \in \{50, 100, 1000, 3000, 5000\}$. For figures on the left, the data is generated from $\mathsf{Poiss}(2)$ to ensure the model is well-specified; for plots on the right, the data is generated from $\mathsf{NegBin}(2, 0.01$ to introduce model-data mismatch. All SPCs choose proportion as $q = 0.5$ and for divided SPCs, the number of splits is set as $k = N^{0.49}$.

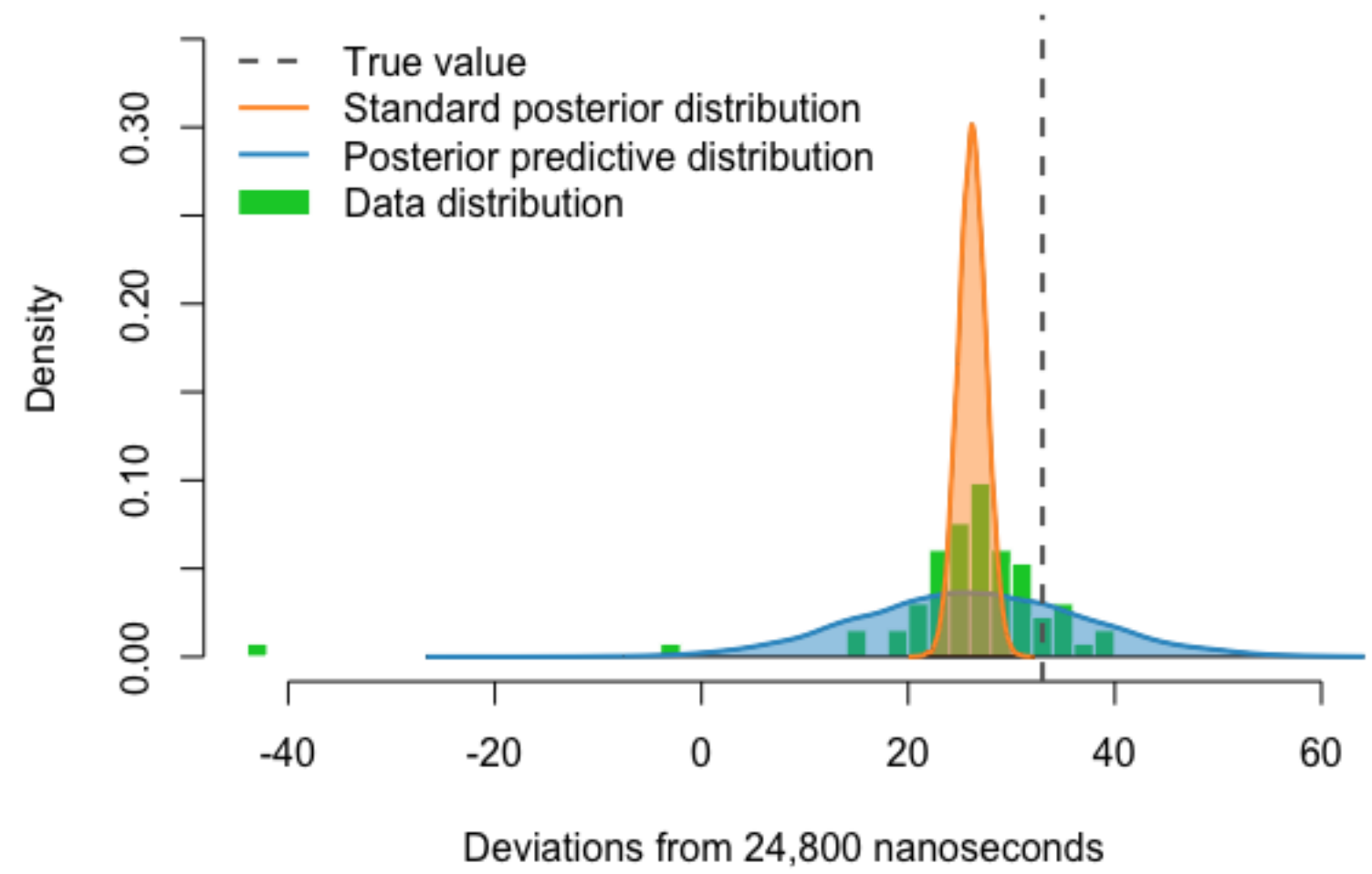

Figure B.8: Light speed data: The histogram of light data, the standard posterior and posterior prediction density curves. The vertical dashed line is the "true value" based on the currently accepted value of speed of light, $\nu = 33.0$.

Table 1: Two-sided p-values for light data.

**Method**	**5th quantile**	**Mean**	**95th quantile**	**Std Dev**	**MSE**
PPC	0.004	0.996	0.010	0.944	0.894
single 0.5-SPC	0.012	0.608	0.762	0.000	0.000
divided 0.5-SPC, $k = \lfloor N^{0.49} \rfloor$	0.592	0.533	0.259	0.239	0.200

while the PPC results in a small p-value for the right quantile. Thus, we find that the single SPC captures complementary (but overlapping) forms of misspecification compared to the PPC. On the other hand, as expected in this small-data regime, the divided SPCs yield p-values far from zero.

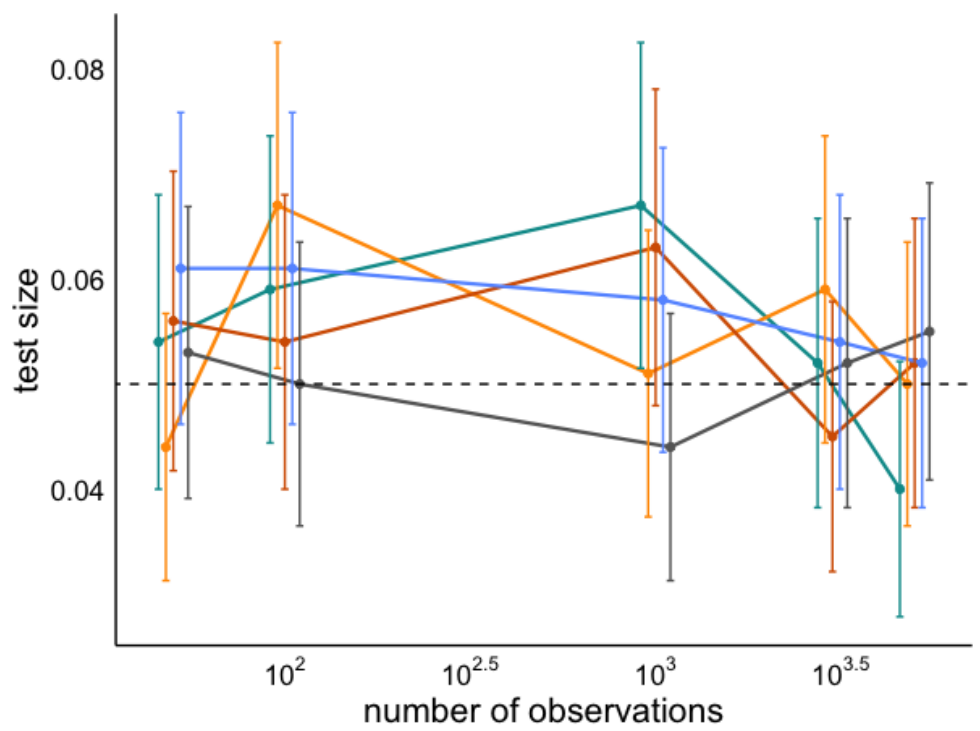

(a) mean

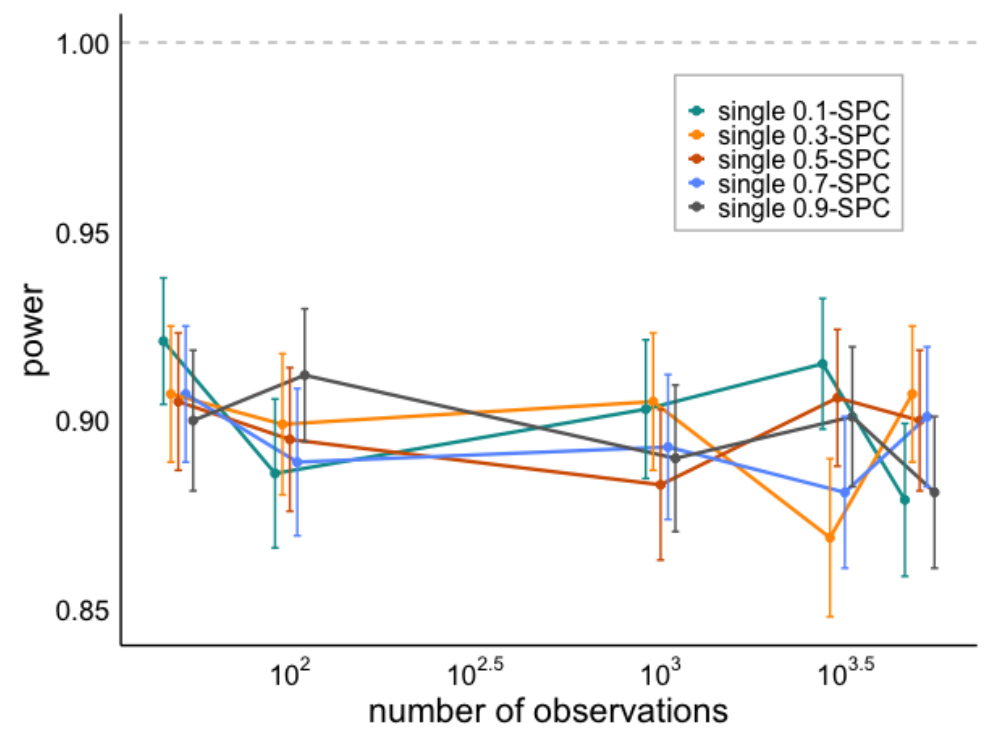

(b) mean

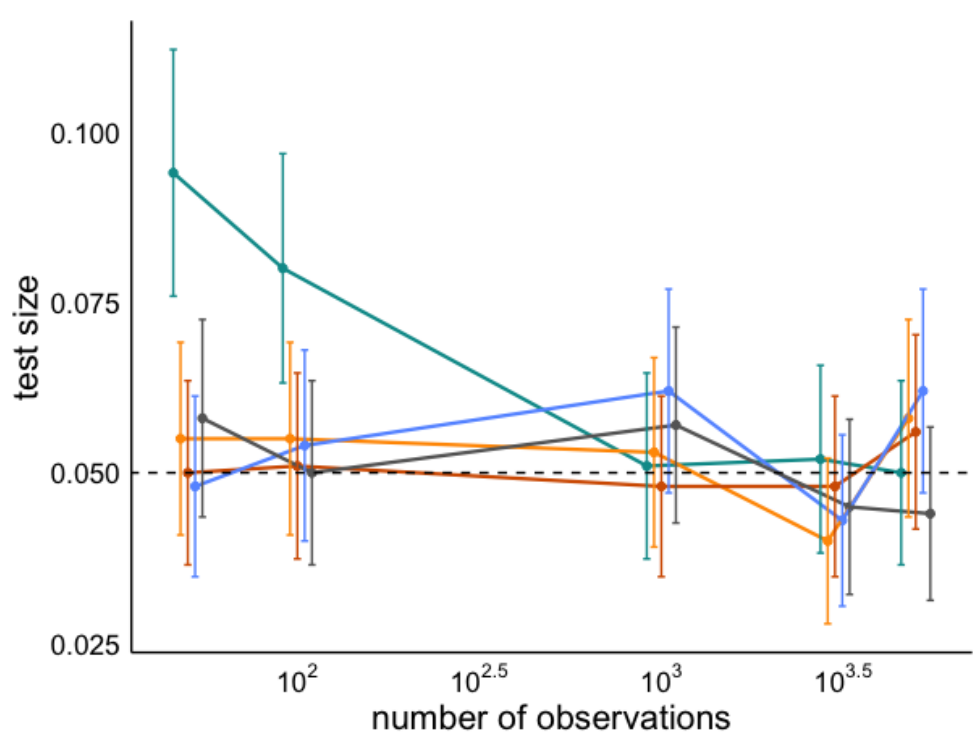

(c) 2nd moment

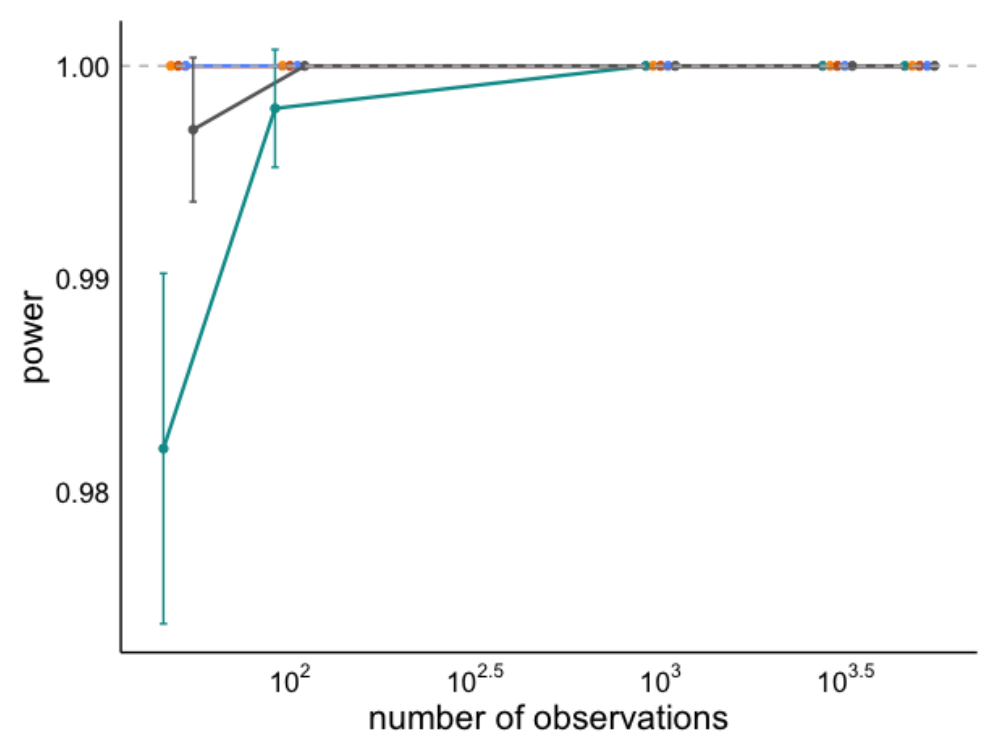

(d) 2nd moment

Figure B.9: Gaussian location model: Test size and power plots in the log scale for single SPC with different split proportions $q \in \{0.1, 0.3, 0.5, 0.7, 0.9\}$, under the Gaussian location model. For plots on the left, the data is generated from $\mathcal{N}(0, 1)$ and for plots on the right, the data is generated from $\mathcal{N}(0, 15^2)$. The vertical bars refer to the confidence interval of each estimate. All estimates of test size and power are computed by 1000 replicate observed data drawn from the generative model. We display experiments for $N \in \{50, 100, 1000, 3000, 5000\}$ to show how test size and power change as number of observations increases. The dashed horizontal line in test size plots indicates the significance level $\alpha = 0.05$ and the dashed line in power plot shows the optimal power 1.

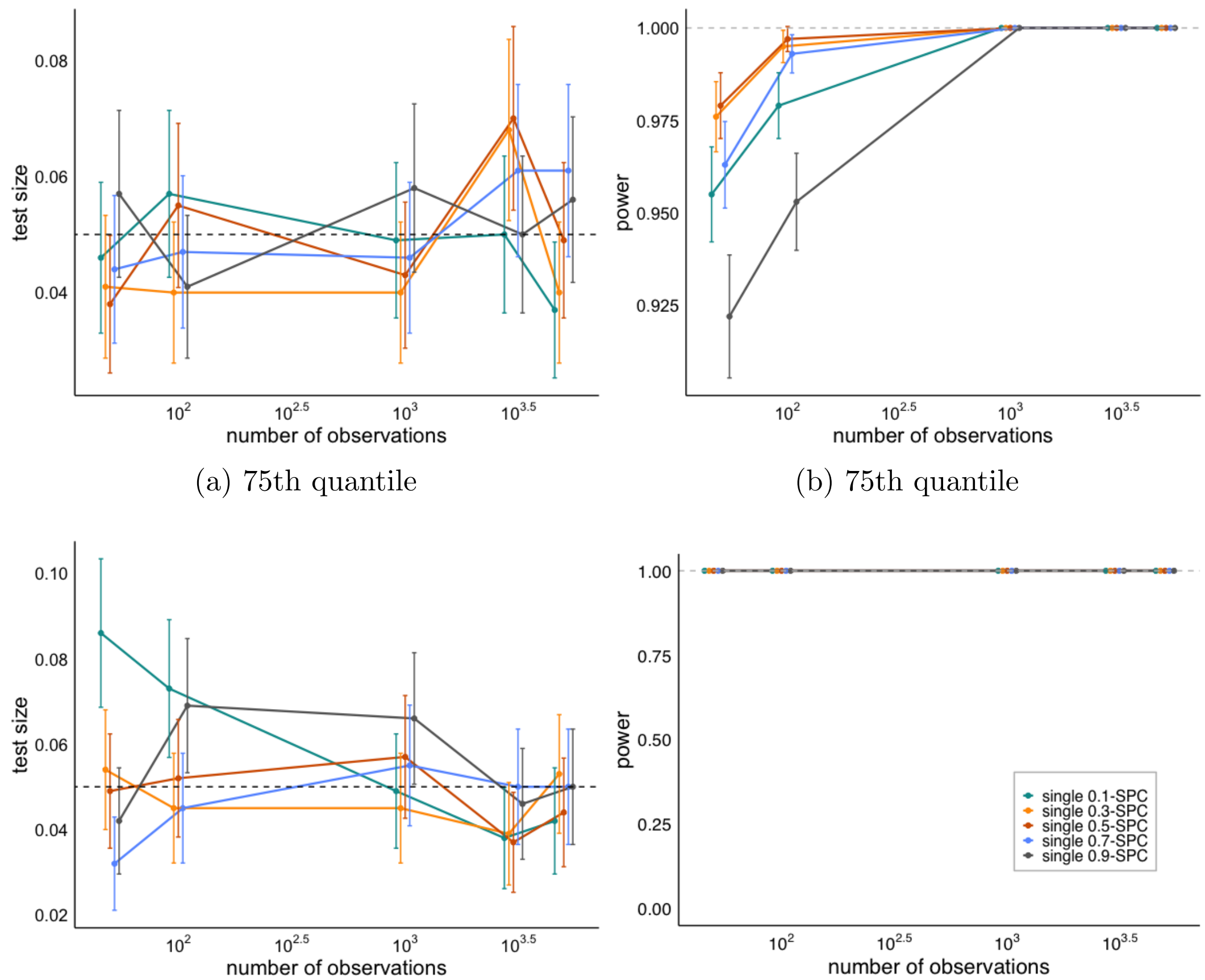

(a) 75th quantile

(b) 75th quantile

(c) MSE

(d) MSE

Figure B.10: Gaussian location model: Test size and power plots (in log scale) for single SPC with different split proportions $q \in \{0.1, 0.3, 0.5, 0.7, 0.9\}$. For plots on the left, the data is generated from $\mathcal{N}(0, 1)$ and for plots on the right, the data is generated from $\mathcal{N}(0, 15^2)$. See caption for Fig. B.9 for further explanation.

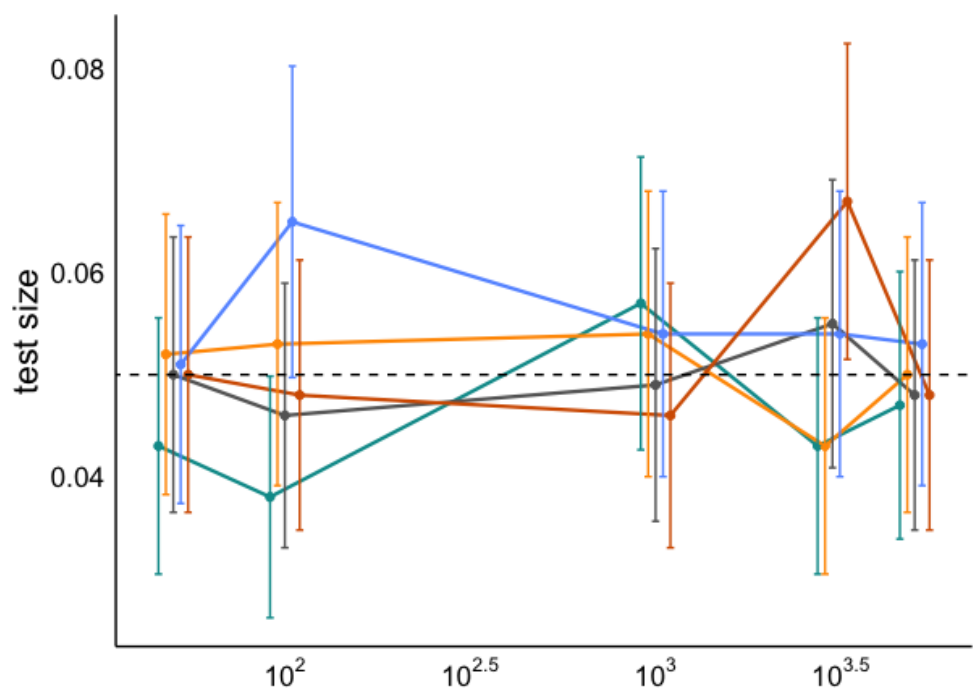

(a) mean

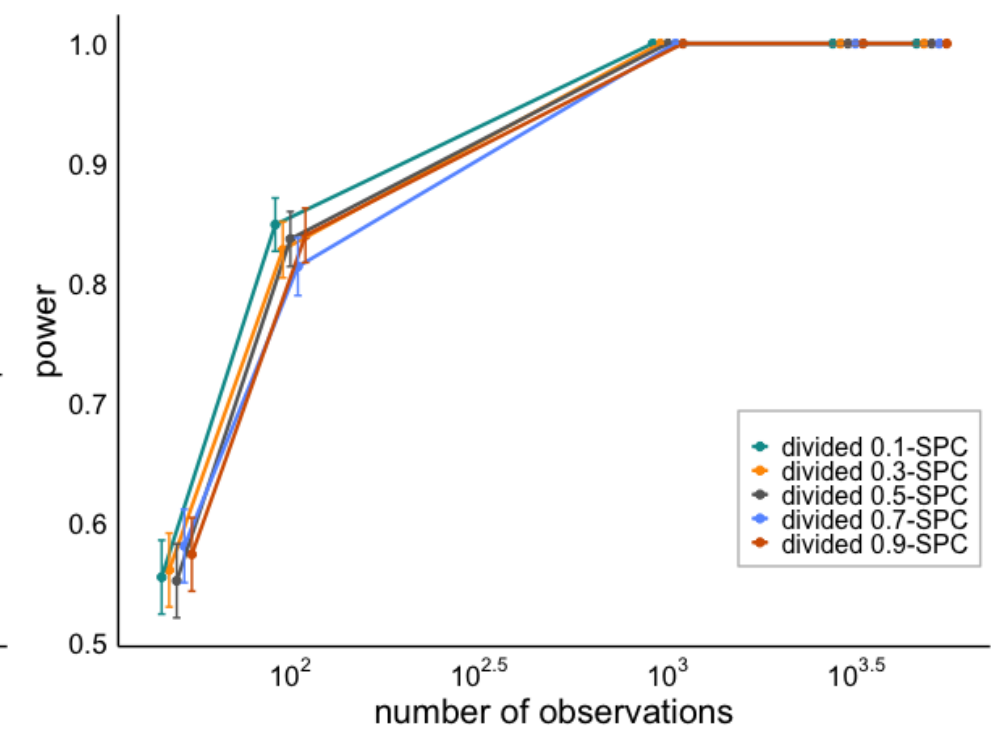

(b) mean

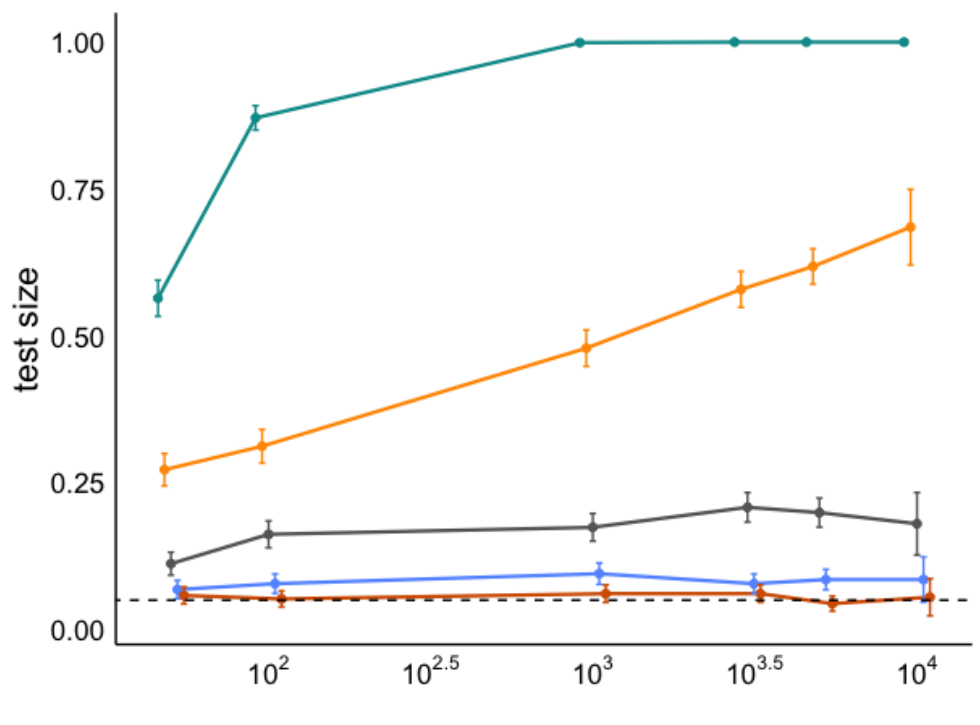

(c) 2nd moment

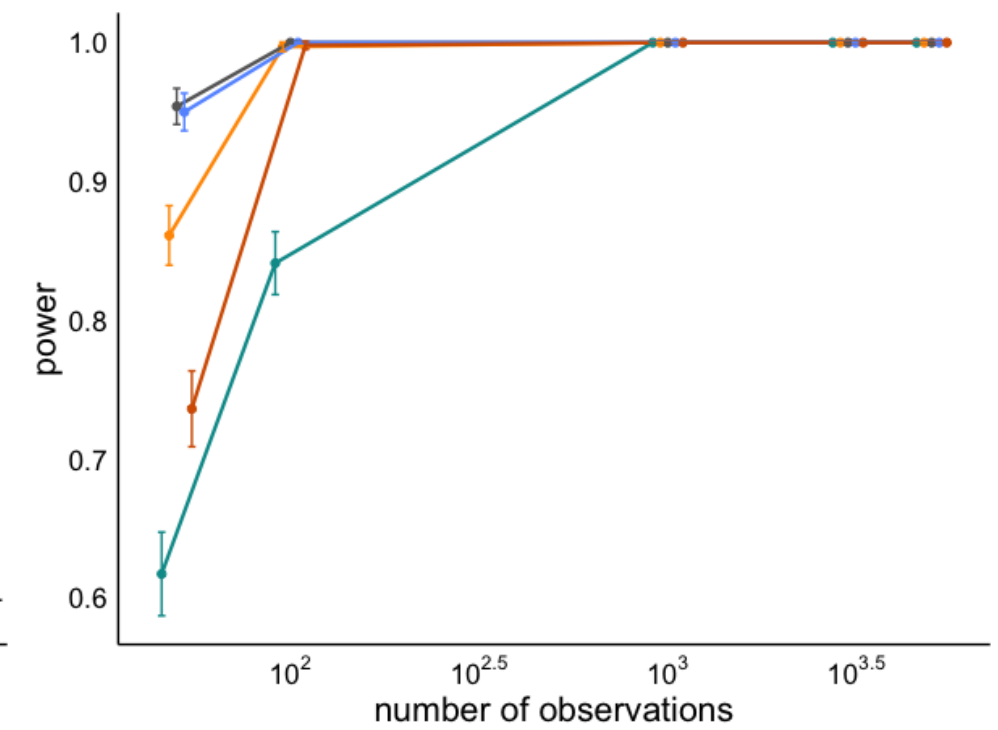

(d) 2nd moment

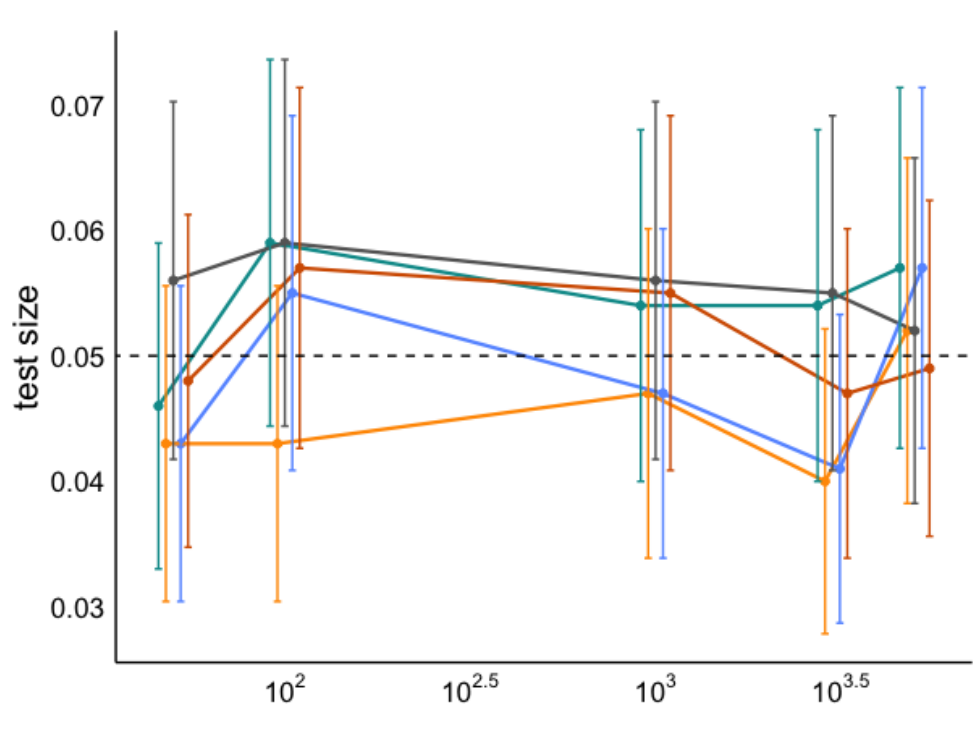

(e) 75th quantile

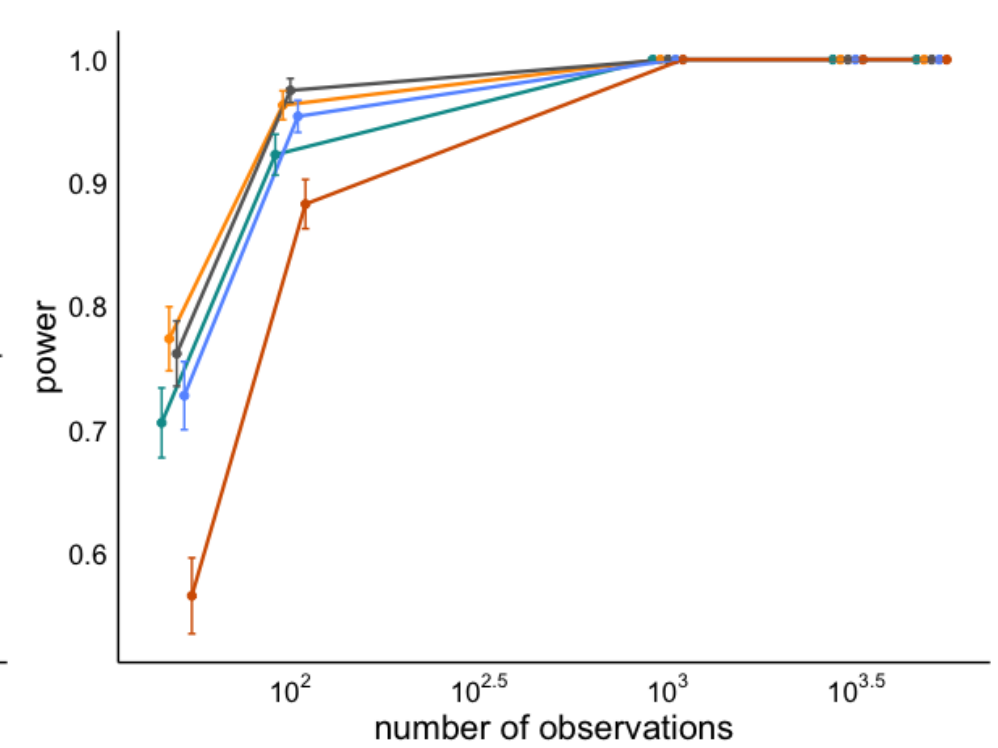

(f) 75th quantile

Figure B.11: Gaussian location model: Test size and power plots in the log scale for divided SPC with identical $k = N^{0.49}$, varying split proportions $q \in \{0.1, 0.3, 0.5, 0.7, 0.9\}$, under the Gaussian location model. For plots on the left, the data is generated from $\mathcal{N}(0, 1)$ and for plots on the right, the data is generated from $\mathcal{N}(0, 15^2)$. We include experiments for $N \in \{50, 100, 1000, 3000, 5000\}$.

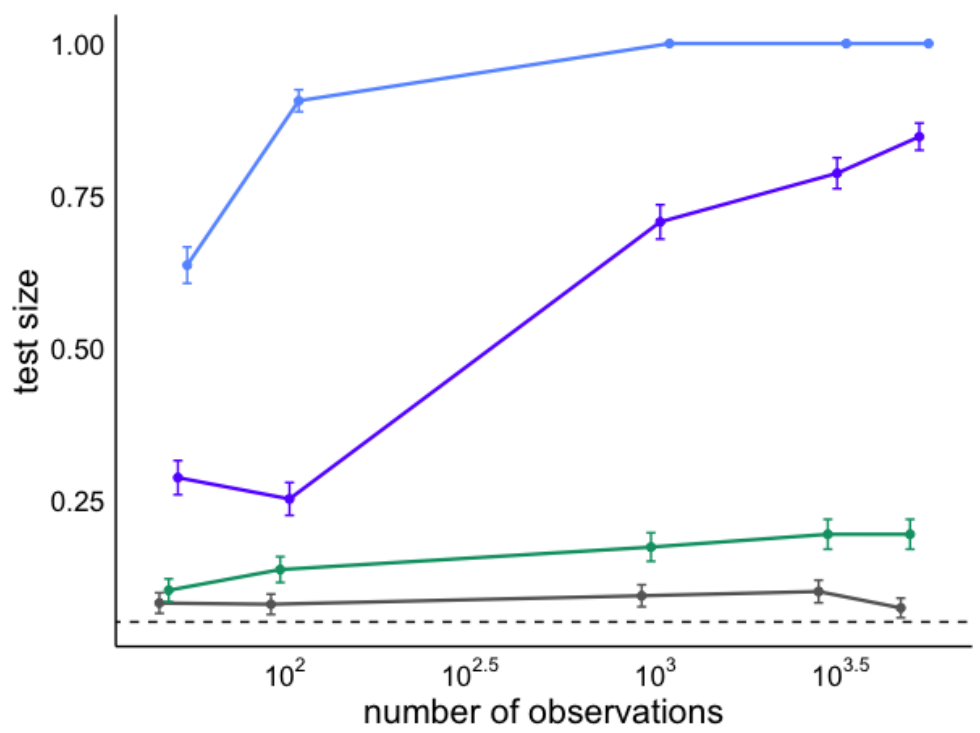

(a) 2nd moment

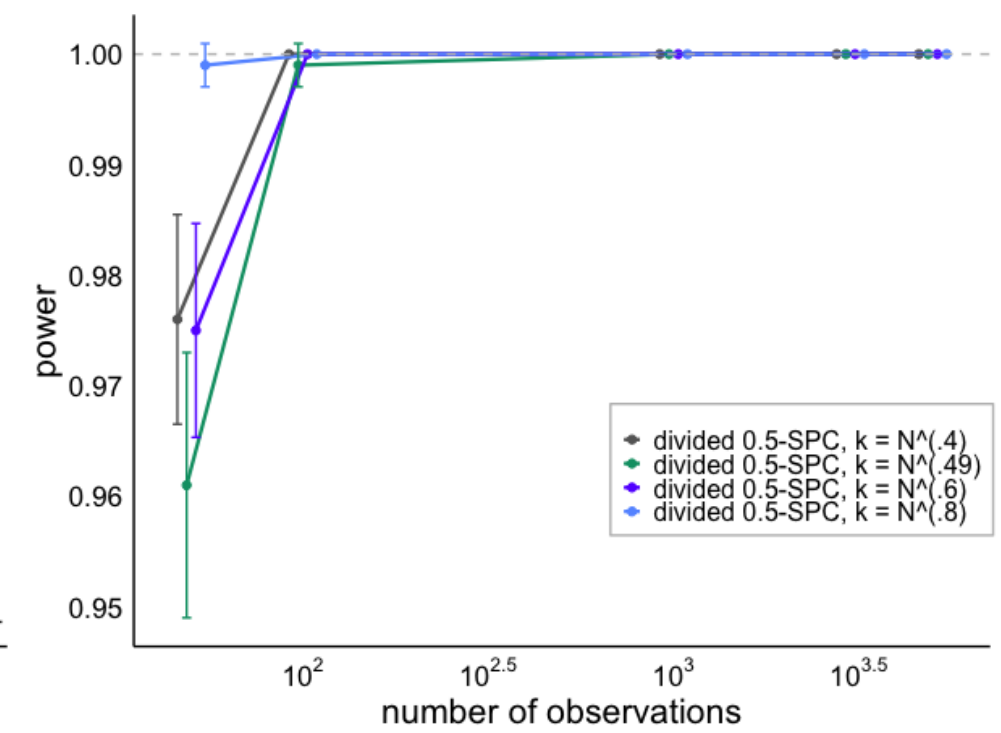

(b) 2nd moment

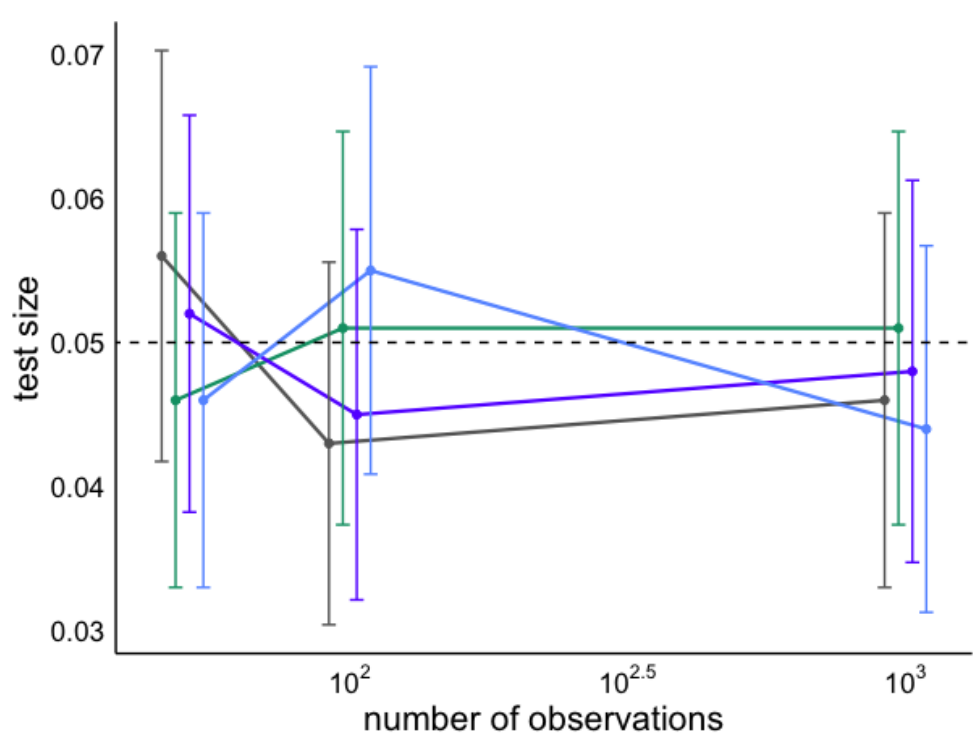

(c) 75th quantile

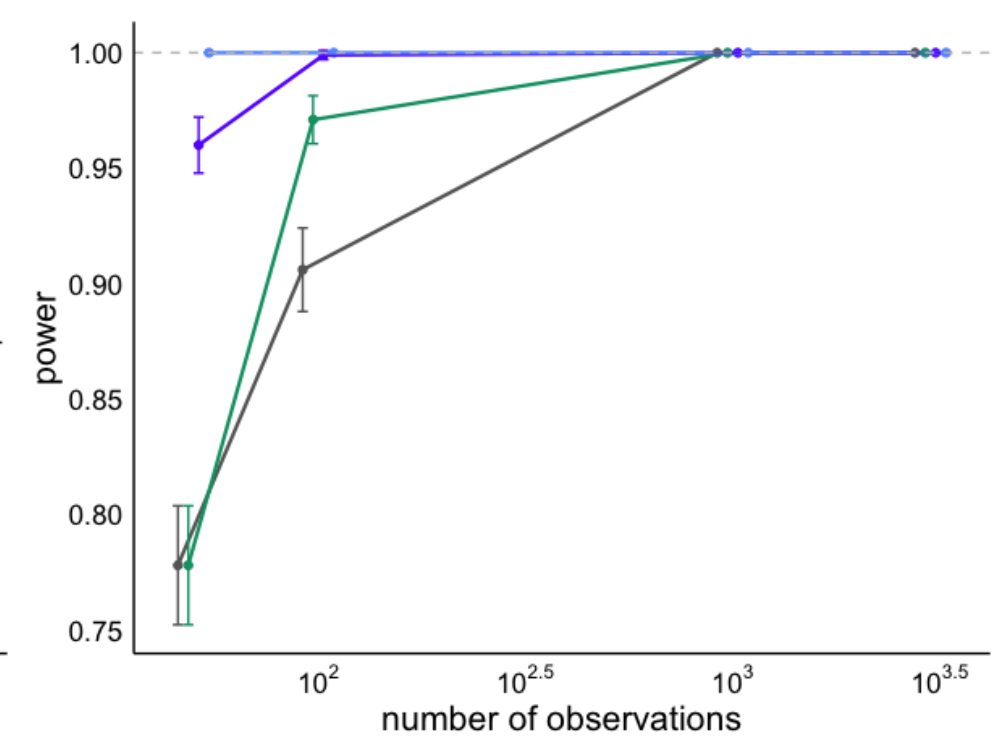

(d) 75th quantile

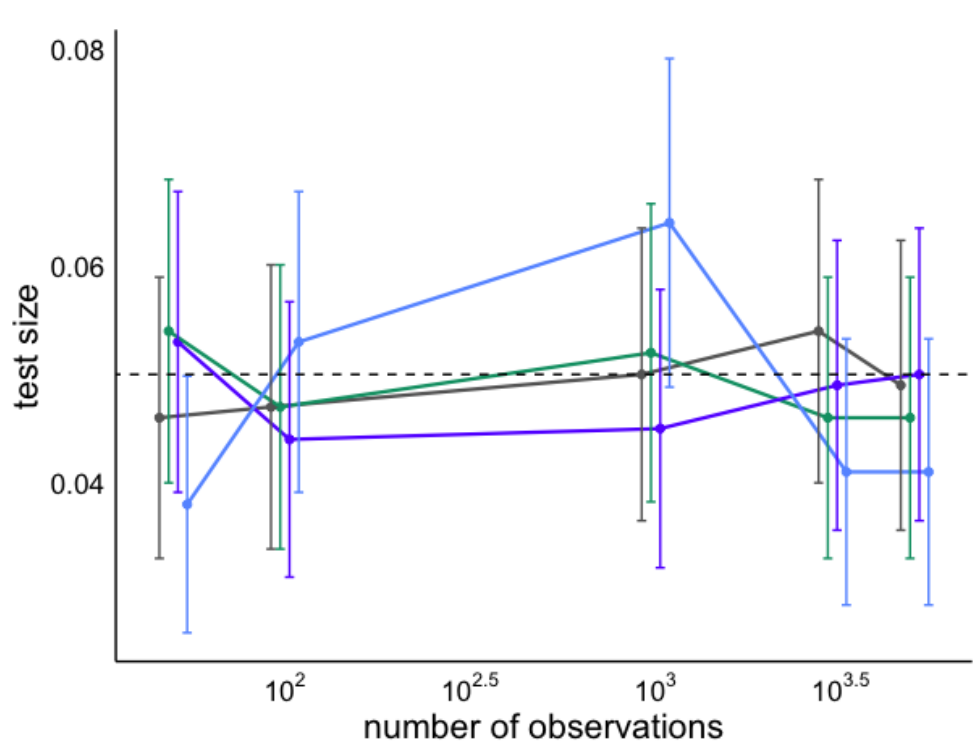

(e) mean

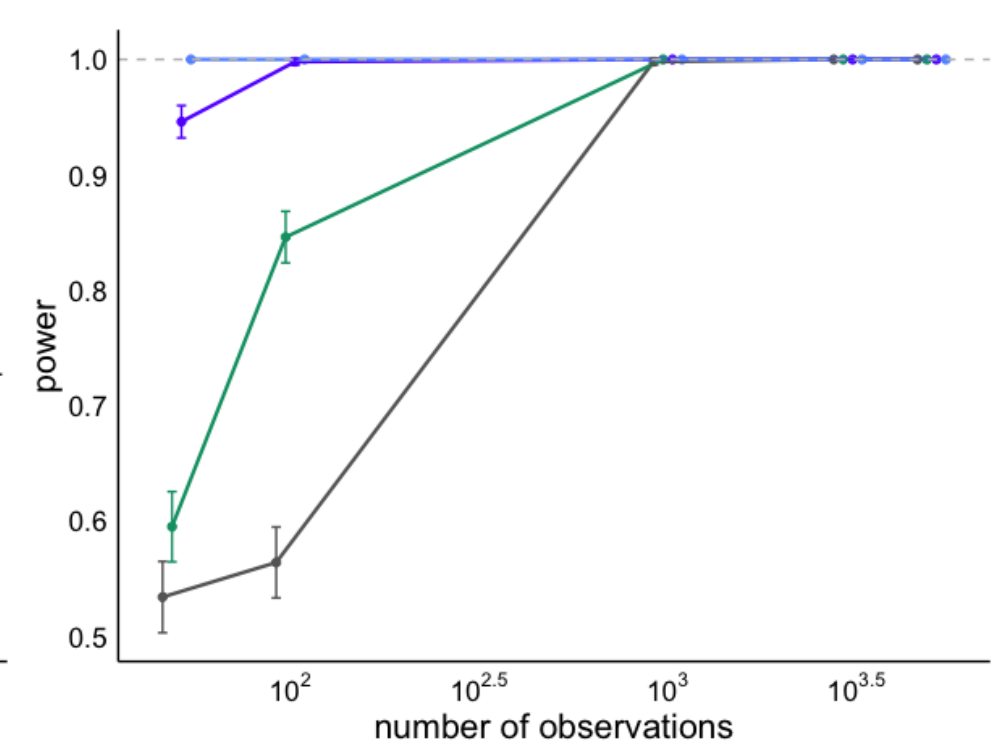

(f) mean

Figure B.12: Gaussian location model: Test size and power plots in the log scale with 2nd moment and 75th quantile statistics for divided SPC under the Gaussian location model. For plots on the left, the data is generated from $\mathcal{N}(0,1)$ and for plots on the right, the data is generated from $\mathcal{N}(0,15^2)$. Divided SPC p-values are computed with identical split proportions $q = 0.5$ with varying number of folds $k \in \{N^{0.4}, N^{0.49}, N^{0.6}, N^{0.8}\}$. We include experiments for $N \in \{50, 100, 1000, 3000, 5000\}$.

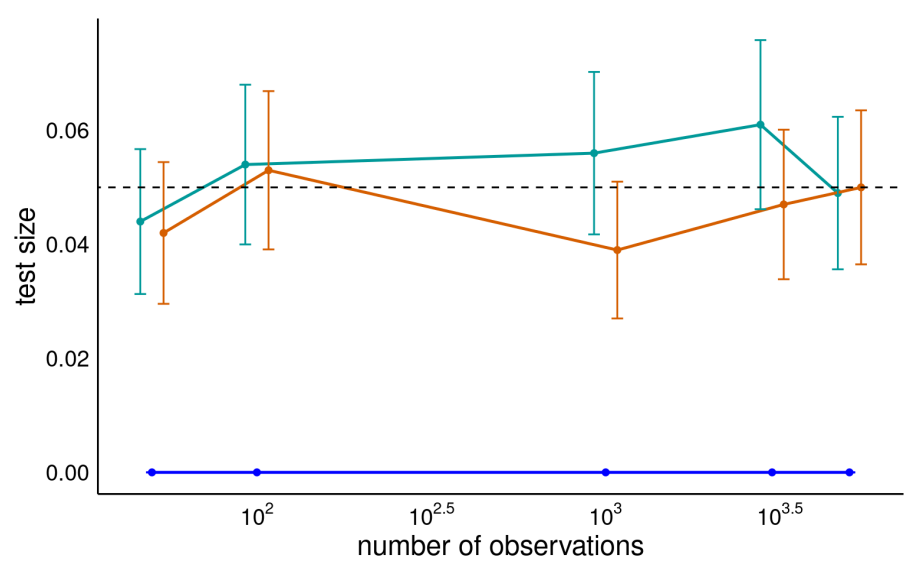

(a) mean

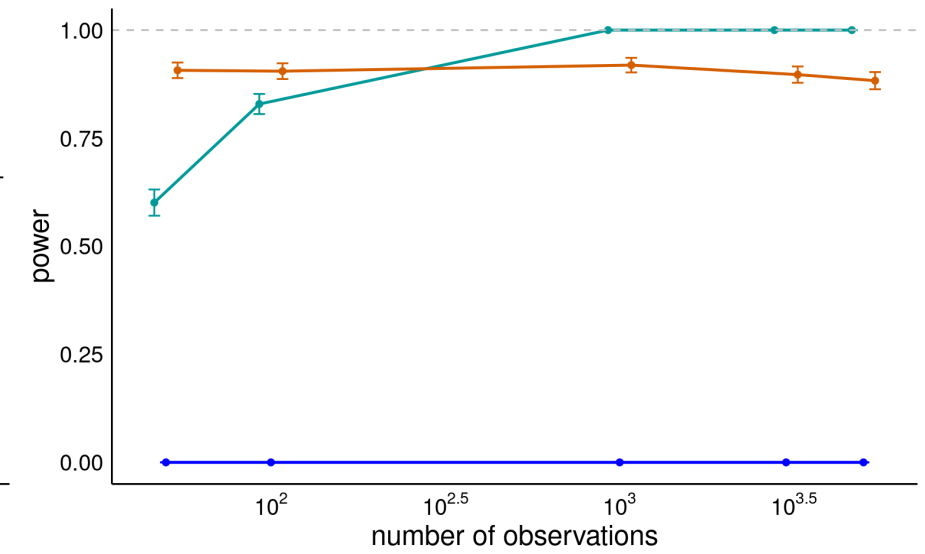

(b) mean

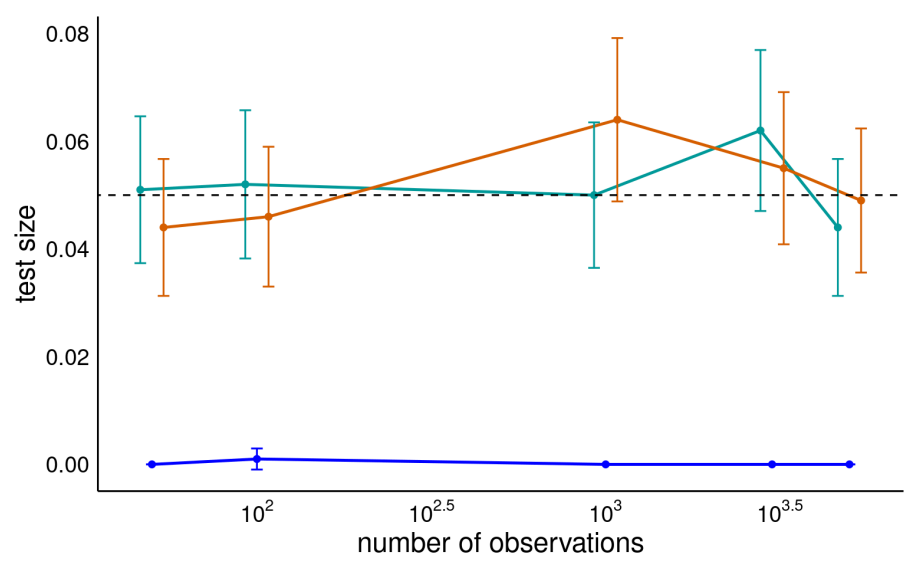

(c) 75th quantile

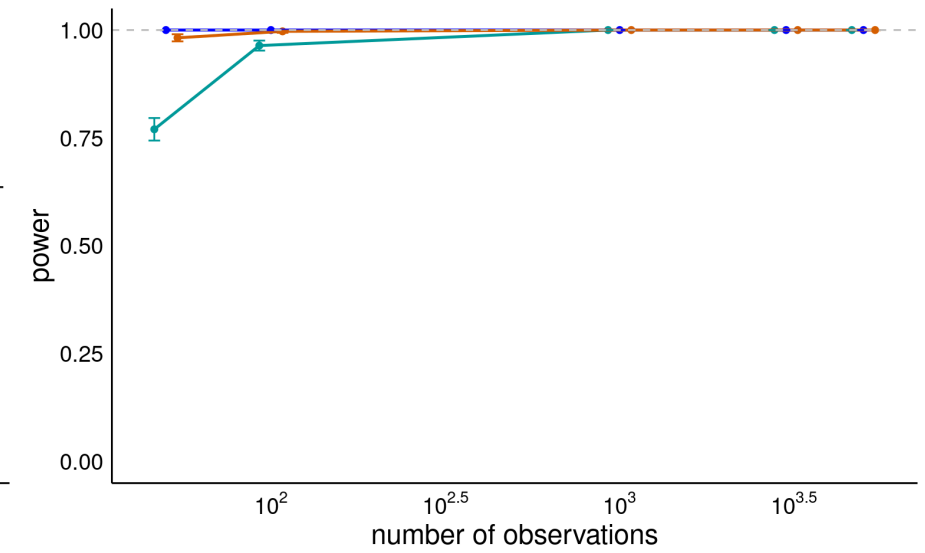

(d) 75th quantile

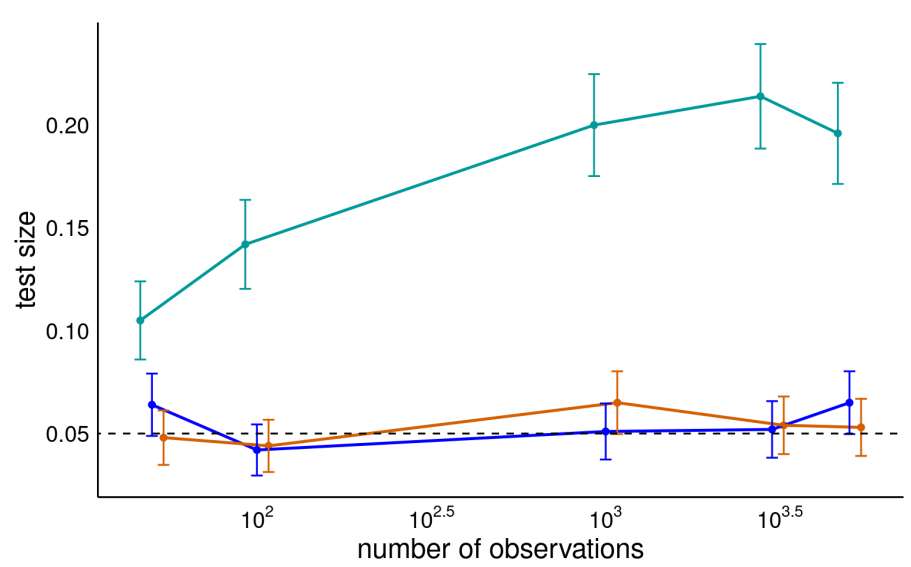

(e) 2nd moment

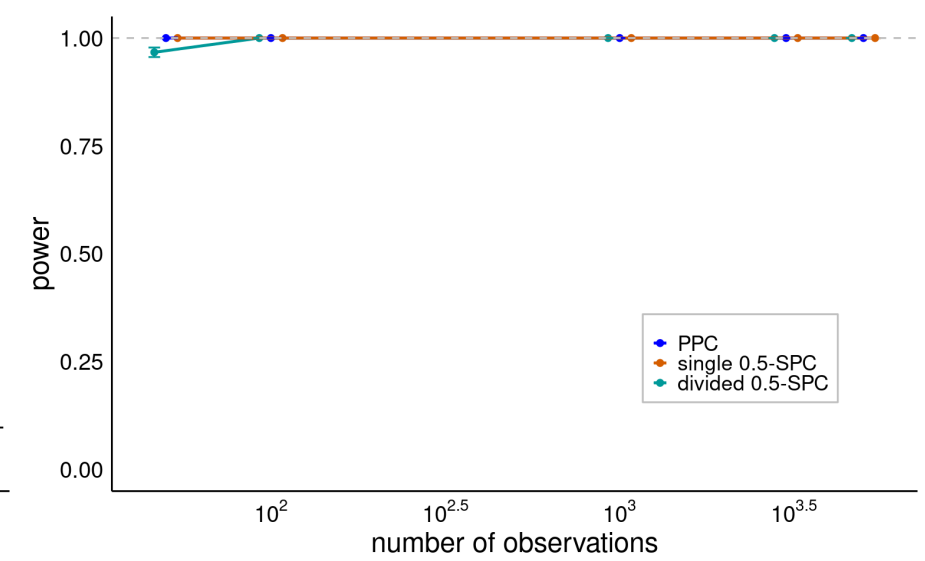

(f) 2nd moment

Figure B.13: Gaussian location model: Test size and power plots in log scale for different checking methods and statistics under Gaussian location model. We display experiments for $N \in \{50, 100, 1000, 3000, 5000\}$. For plots on the left, the data is generated from $\mathcal{N}(0, 1)$ and for plots on the right, the data is generated from $\mathcal{N}(0, 15^2)$. All SPCs choose proportion $q = 0.5$ and for divided SPCs, the number of splits is set as $k = N^{0.49}$.

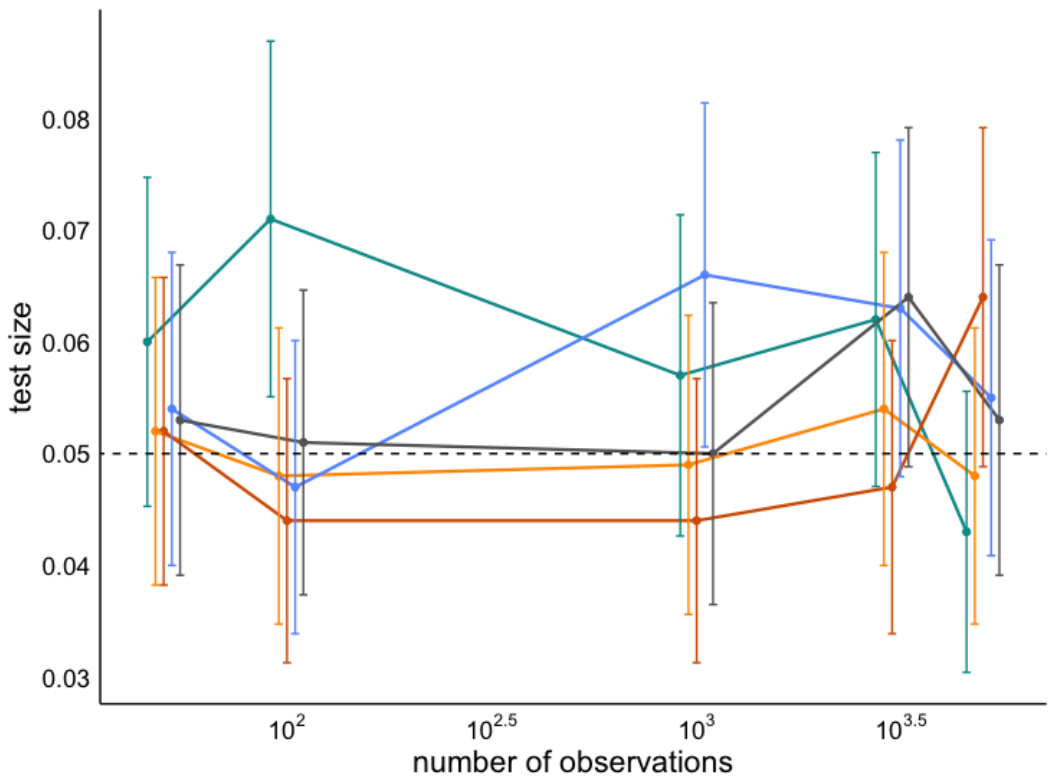

(a) 3rd moment

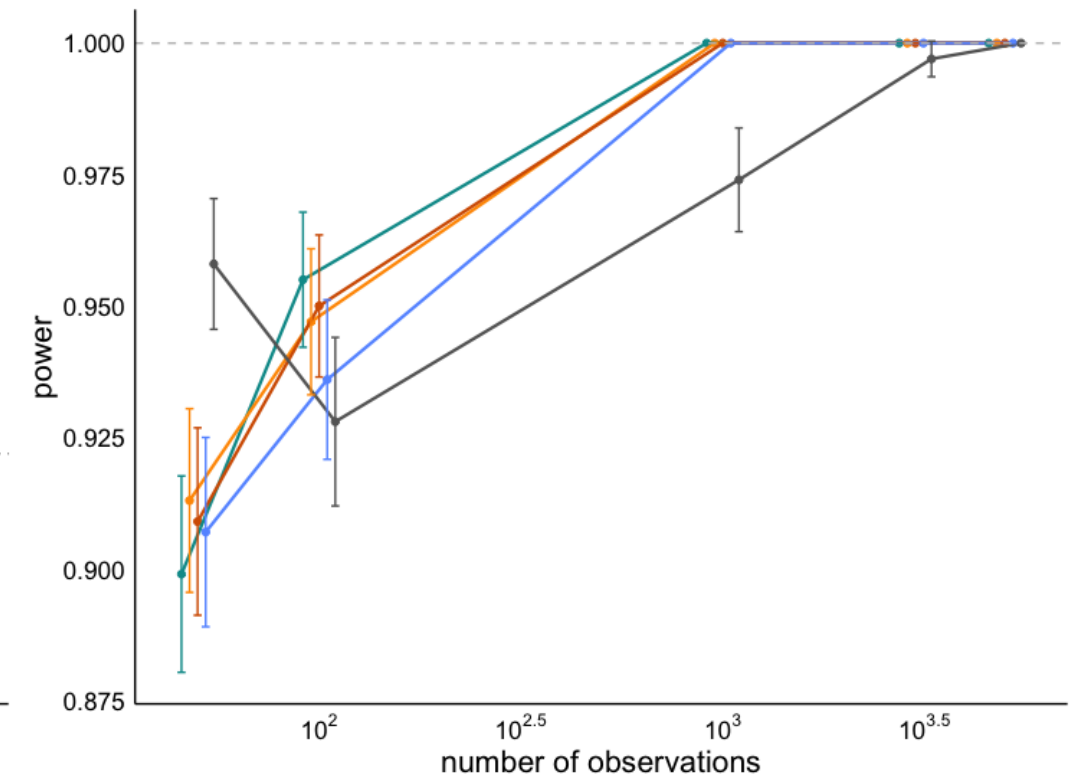

(b) 3rd moment

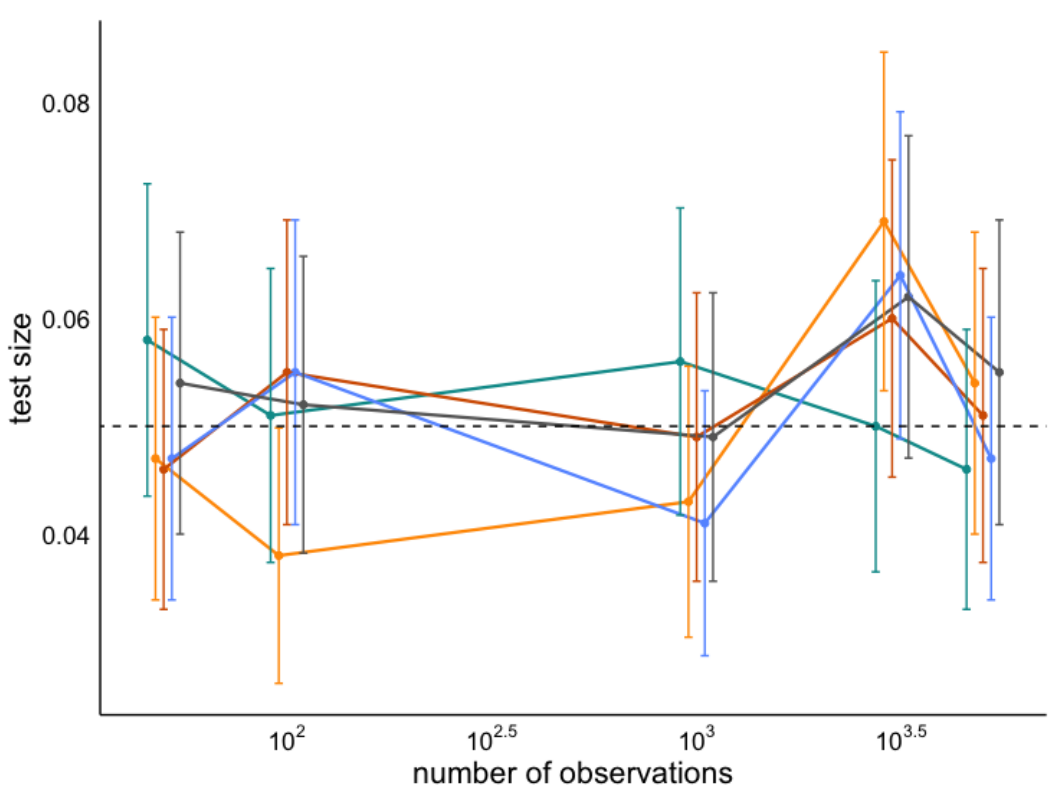

(c) MSE

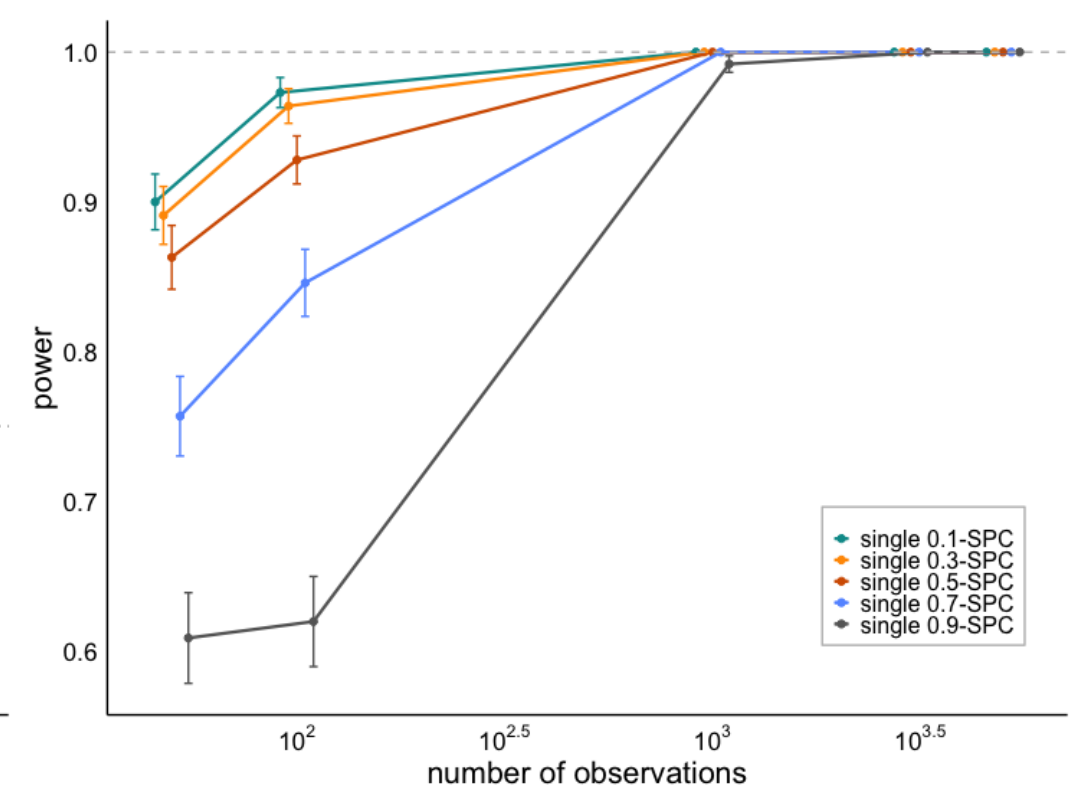

(d) MSE

Figure B.14: Poisson model: Test size and power plots in log scale for single SPC with different split proportions $q \in \{0.1, 0.3, 0.5, 0.7, 0.9\}$, under the conjugate Poisson model. For plots on the left, data is generated from $\mathsf{Poiss}(2)$ and for plots on the right, the data is generated by $\mathsf{NegBin}(2, 0.01)$. See caption for Fig. B.1 for further explanation.

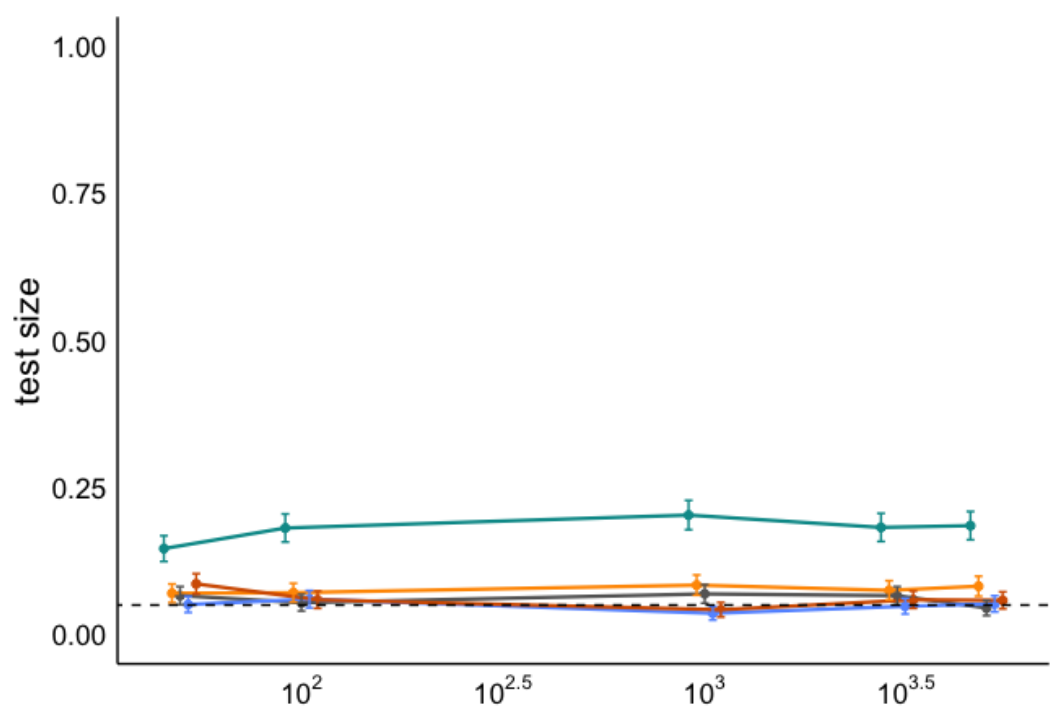

(a) 2nd moment

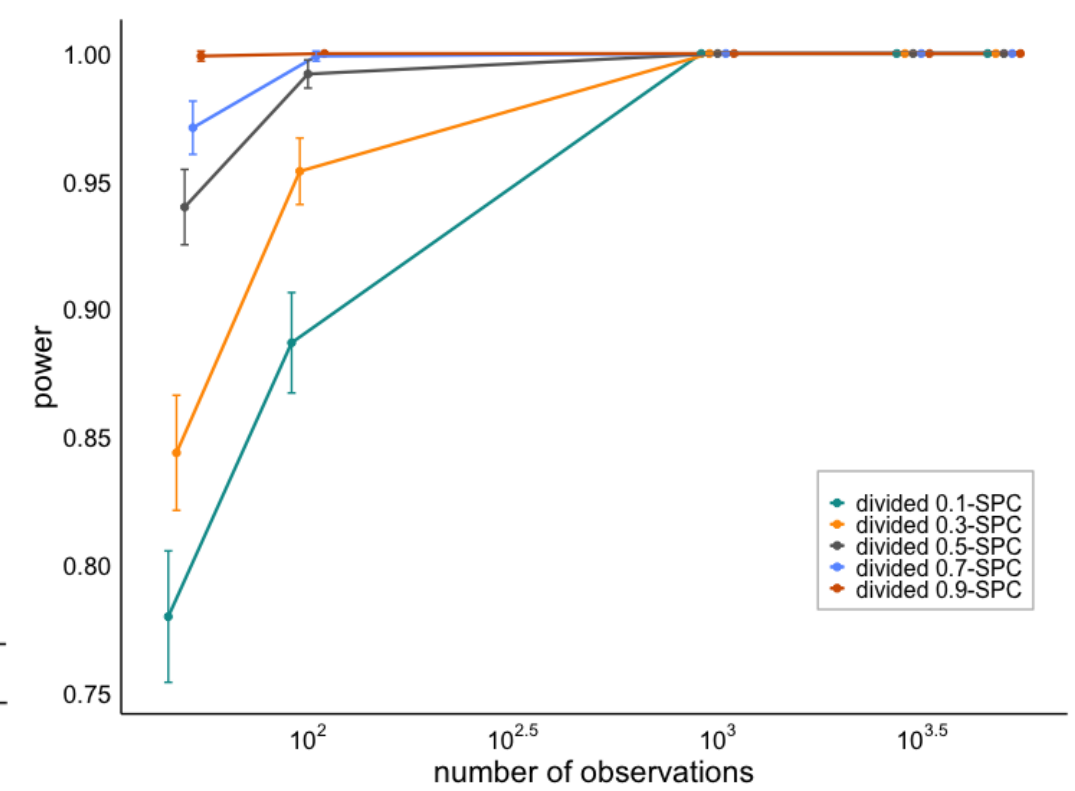

(b) 2nd moment

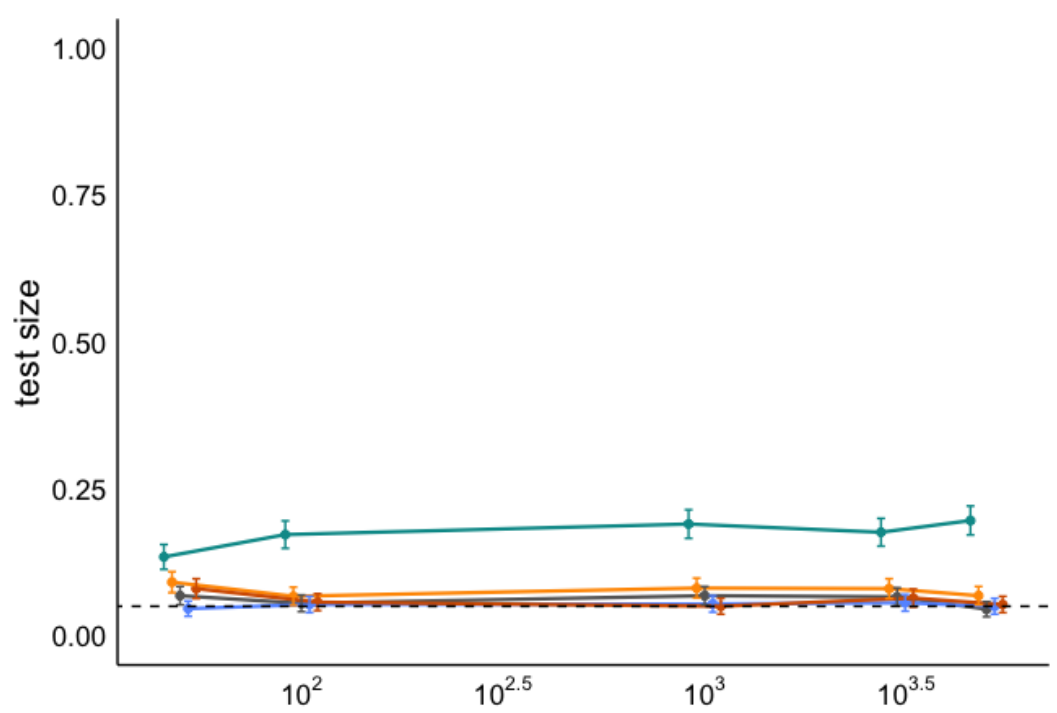

(c) 3rd moment

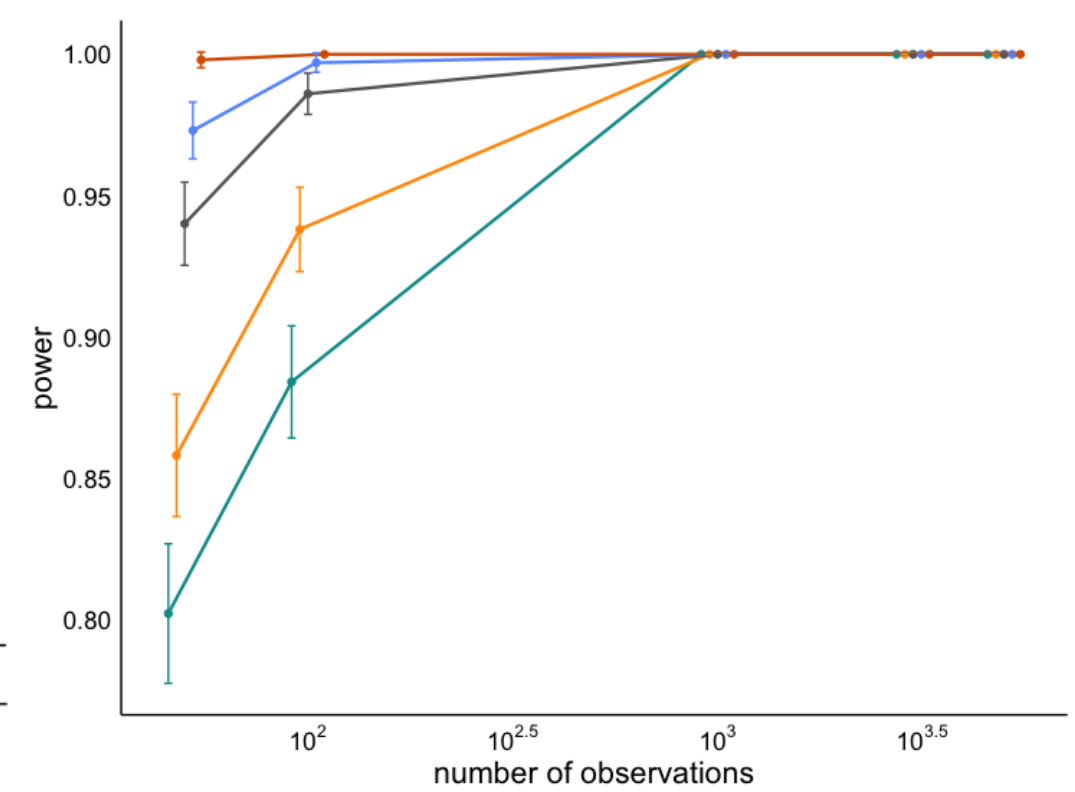

(d) 3rd moment

Figure B.15: Poisson model: Test size and power plots in the log scale for divided SPC with identical $k = N^{0.49}$ but different split proportions $q \in \{0.1, 0.3, 0.5, 0.7, 0.9\}$, under the conjugate Poisson model. For plots on the left, data is generated from $\mathsf{Poiss}(2)$ and for plots on the right, the data is generated by $\mathsf{NegBin}(2, 0.01)$.

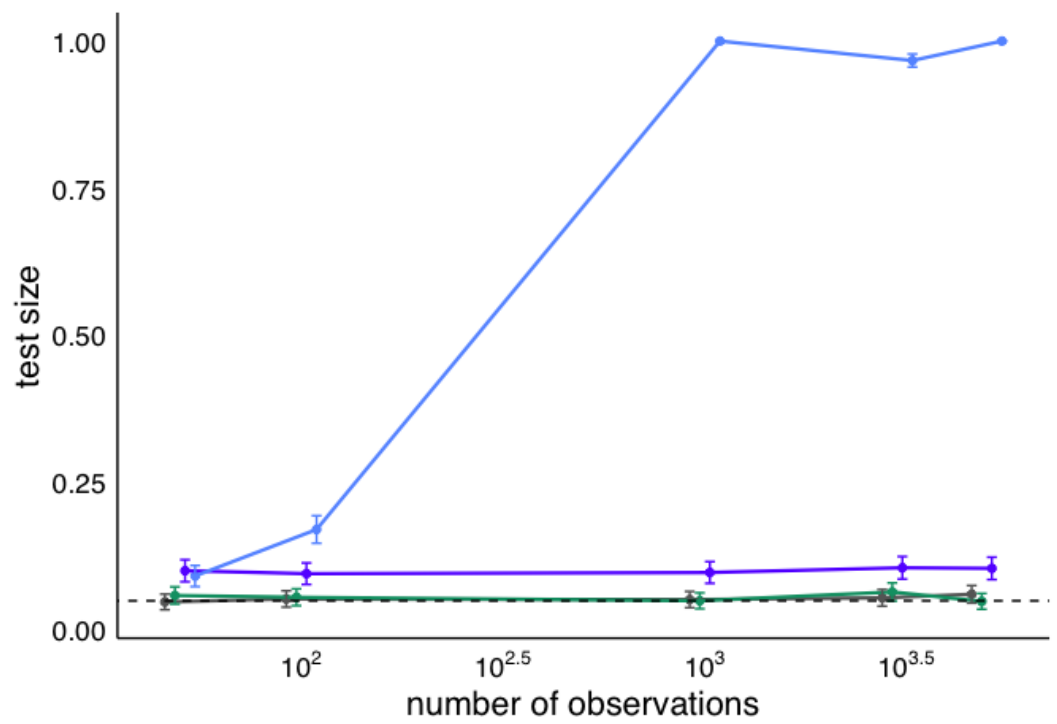

(a) 2nd moment

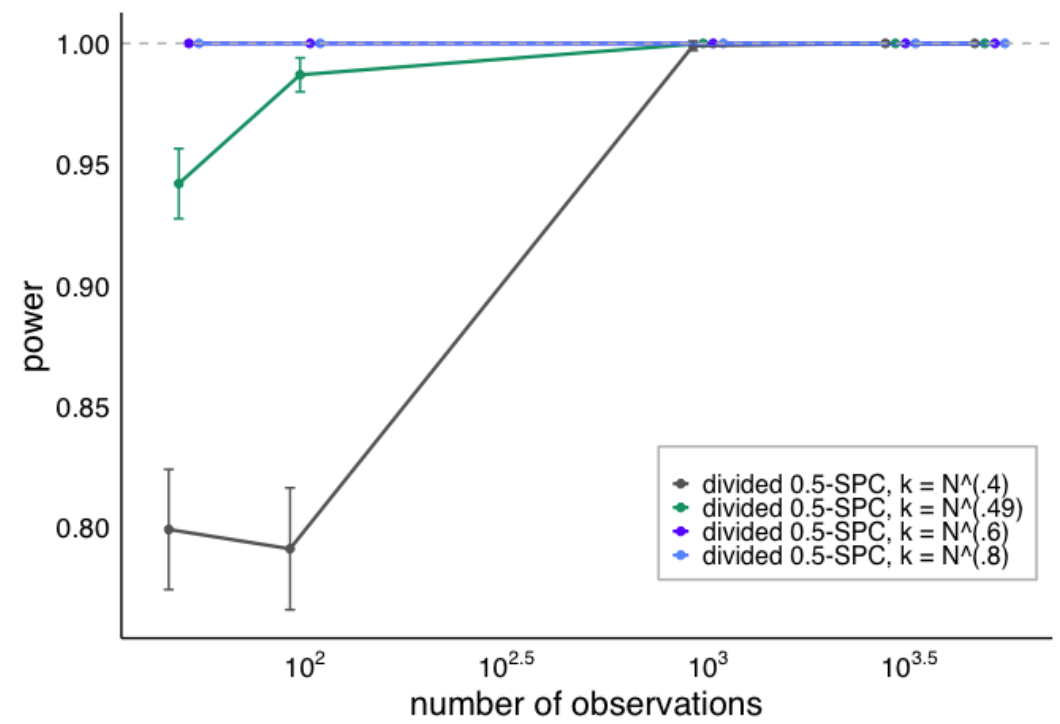

(b) 2nd moment

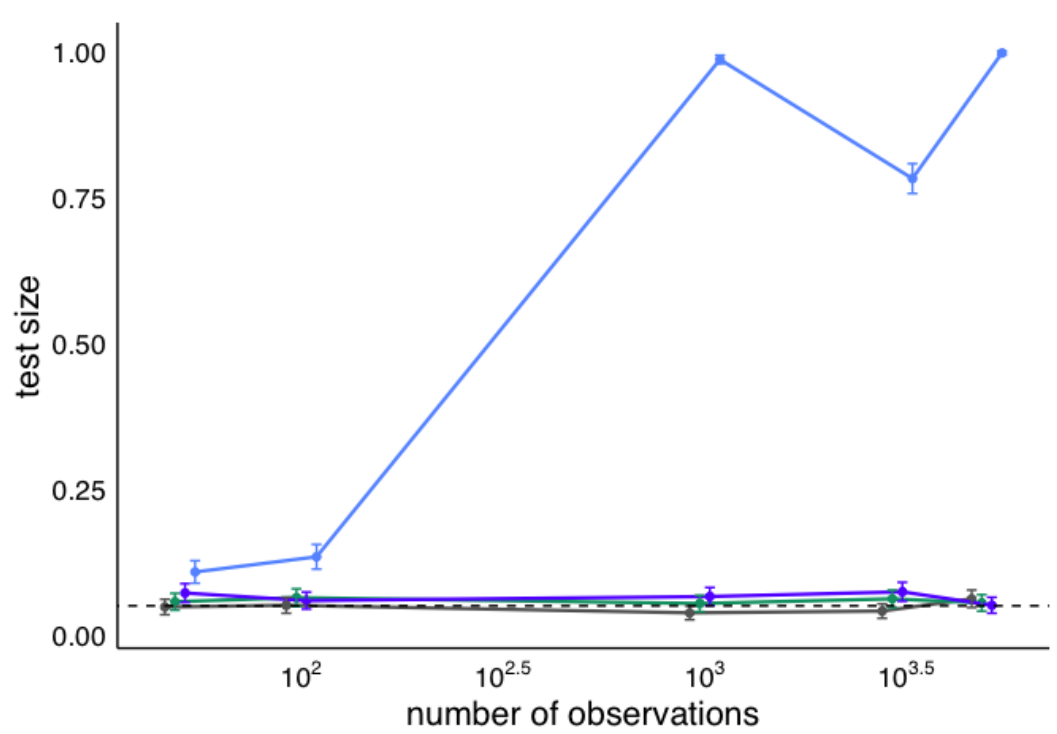

(c) Mean

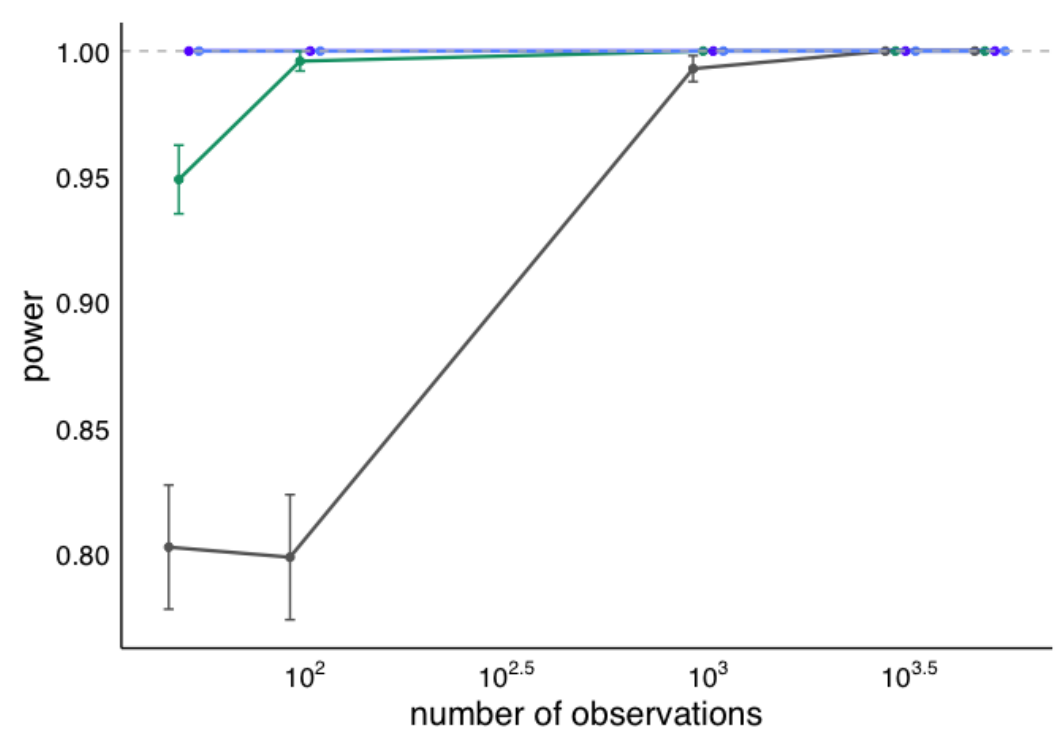

(d) Mean

Figure B.16: Poisson model: Test size and power plots in the log scale for divided SPC with identical split proportion $q = 0.5$ but various $k = N^{0.49}$, under the conjugate Poisson model. For plots on the left, data is generated from $\mathsf{Poiss}(2)$ and for plots on the right, the data is generated by $\mathsf{NegBin}(2, 0.01)$. See caption for Fig. B.4 for further explanation.

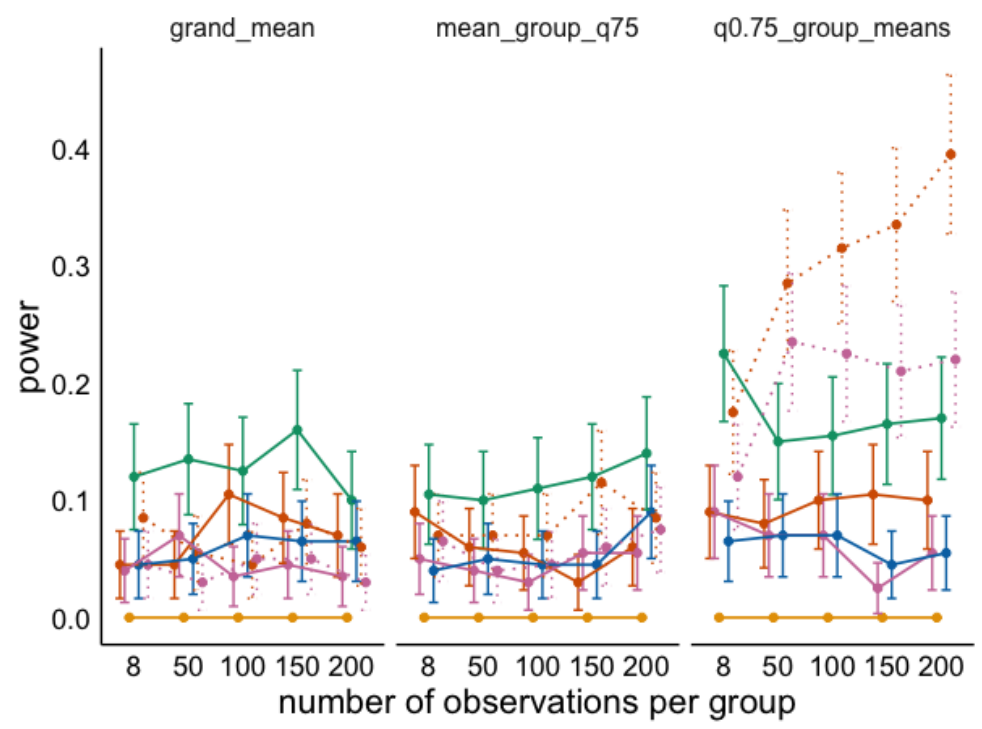

(a) Misspecified across groups

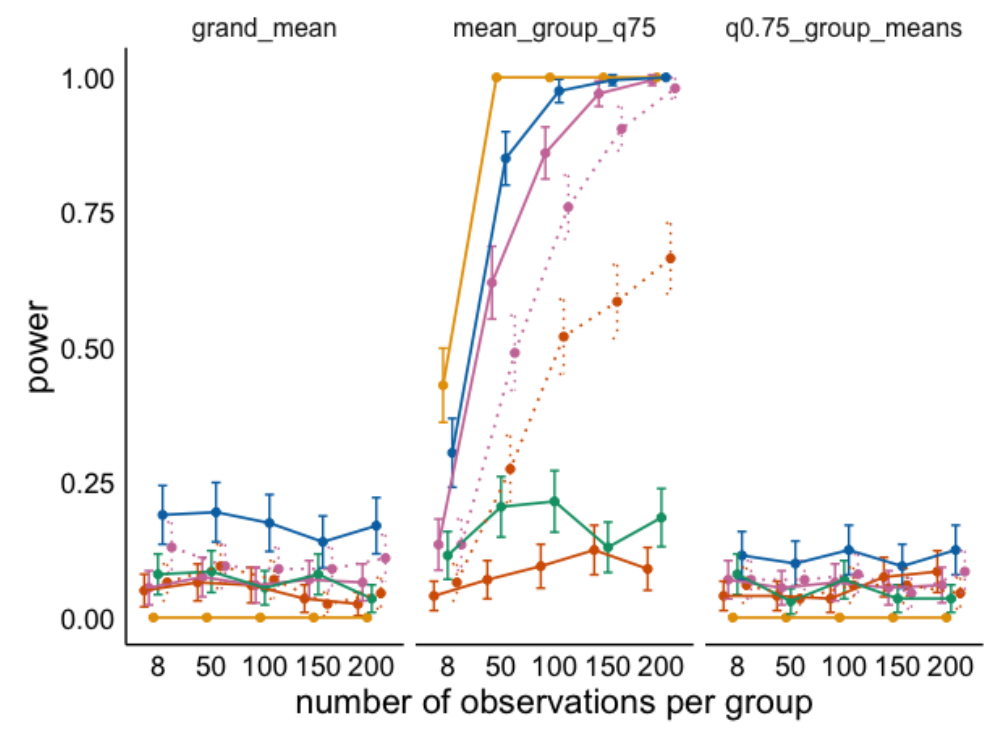

(b) Misspecified within groups

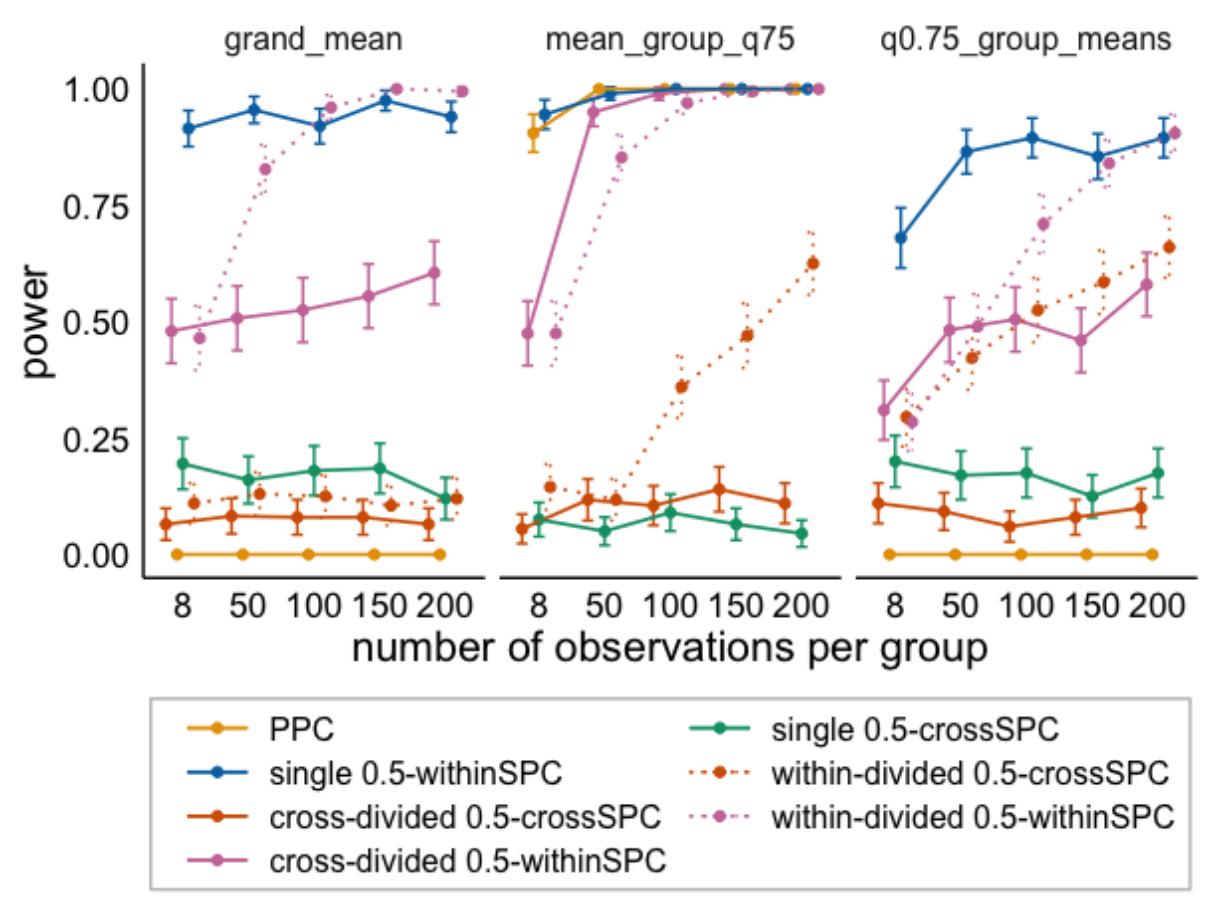

(c) Misspecified across and within groups

Figure B.17: Gaussian hierarchical model: Power plot for different checks and statistics given different types of misspecification with fixed the number of groups $I = 20$. We increase the number of individuals per group to investigate how the power changes accordingly in different scenarios. Powers are estimated with 200 repeated experiments for each scenario. Vertical bars show the 95th confidence interval for each power estimate.

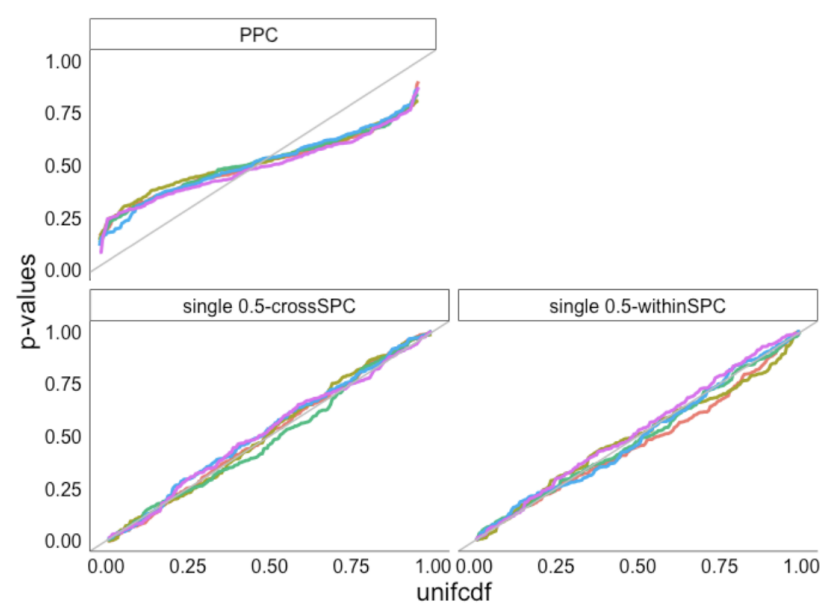

(a) mean of 75th quantiles

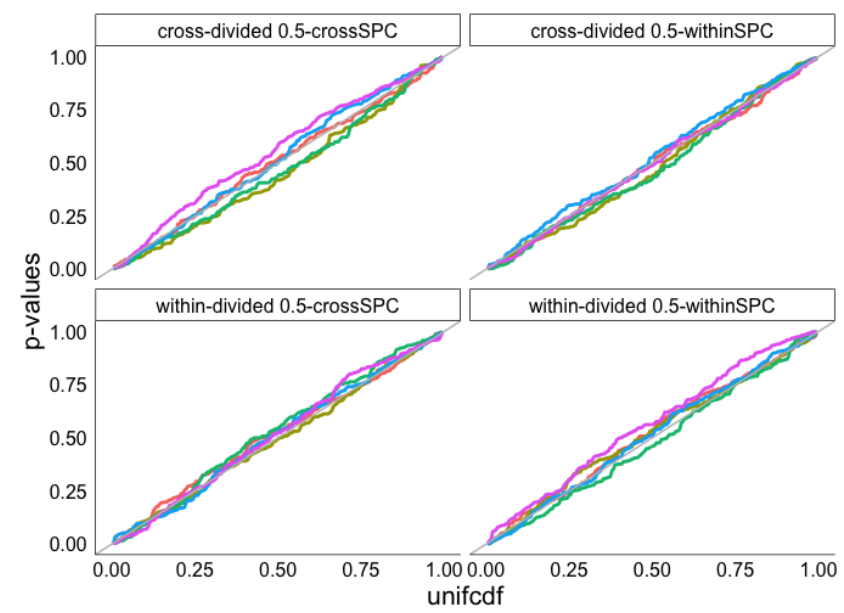

(b) mean of 75th quantiles

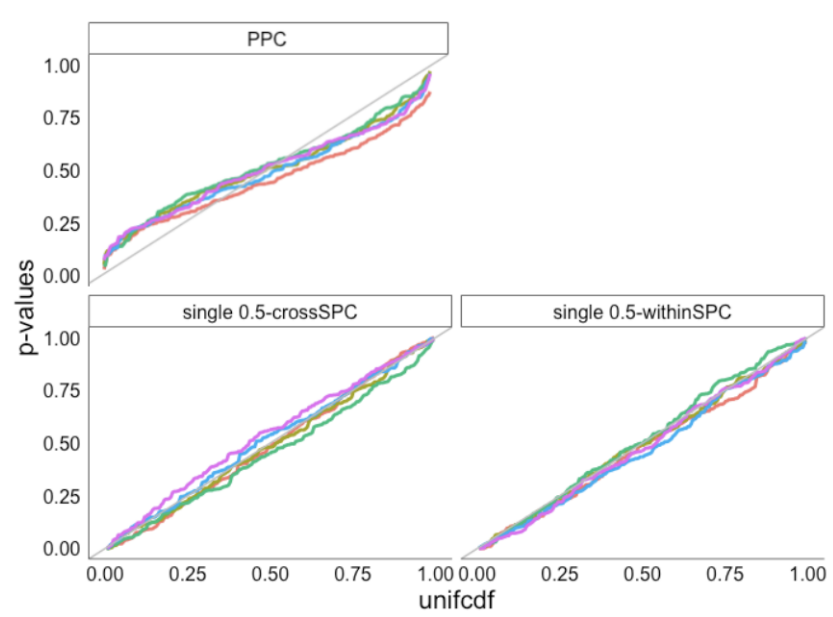

(c) 75th quantile of group means

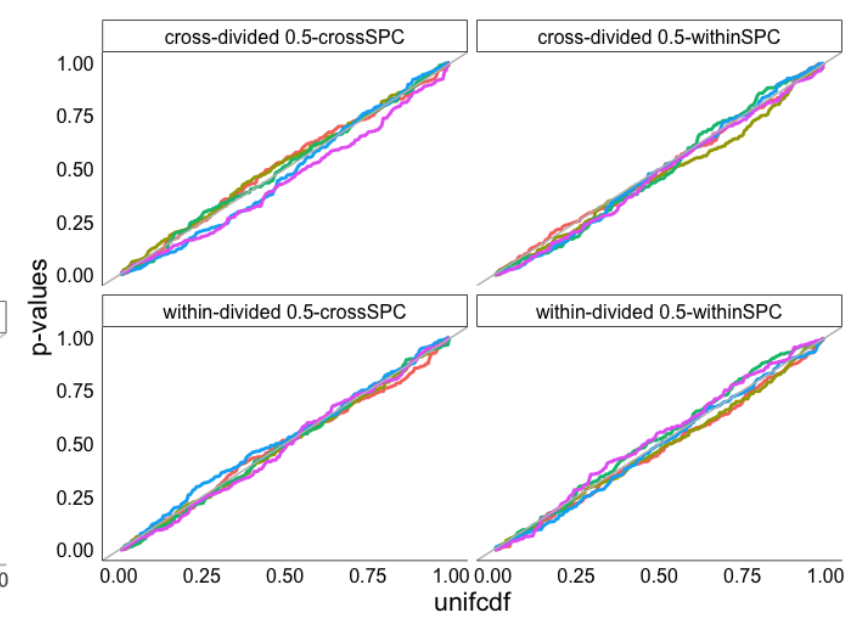

(d) 75th quantile of group means

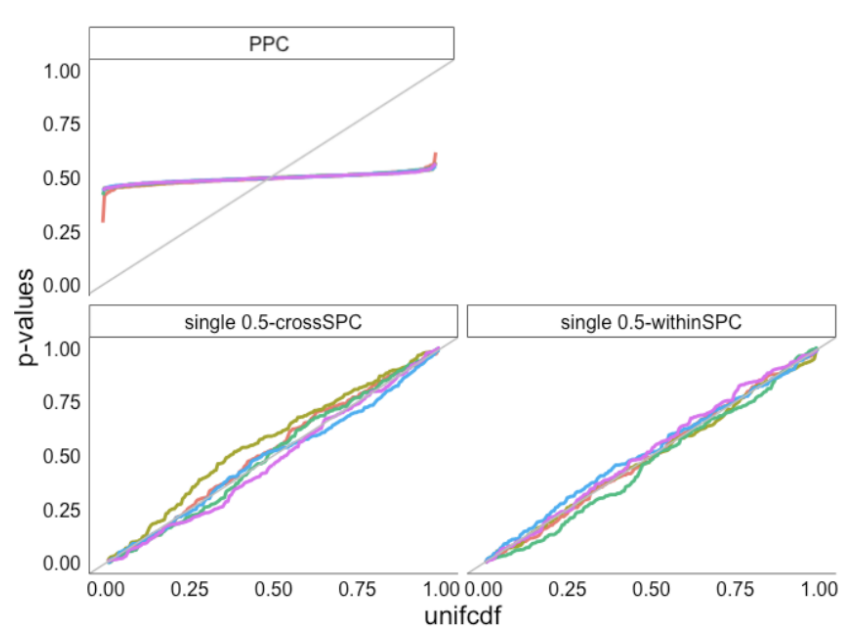

(e) grand mean

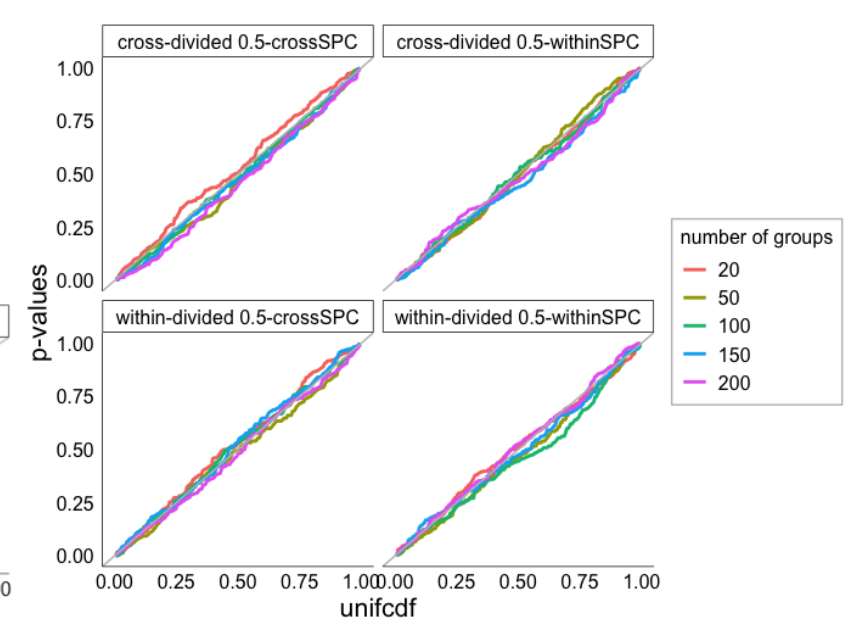

(f) grand mean

Figure B.18: Gaussian hierarchical model: The Q-Q plots of all checks under the well-specified model with grand mean, mean of 75th quantiles and 75th quantile of group means statistic, under the Gaussian hierarchical model.

References

Alberink, I. (1999), 'A Berry–Esseen Bound for U-Statistics in the Non-I.I.D. Case', *Journal of Theoretical Probability* **13**.

Anastasiou, A. & Ley, C. (2017), 'Bounds for the asymptotic normality of the maximum likelihood estimator using the delta method', *Latin American Journal of Probability and Mathematical Statistics* **14**.

Bouchitte, G., Jimenez, C. & Mahadevan, R. (2007), 'A new l^∞ estimate in optimal mass transport', *Proceedings of the American Mathematical Society* **135**, 3525–3535.

Boukai, B. (1990), 'An explicit expression for the distribution of the supremum of brownian motion with a change point', *Communications in Statistics - Theory and Methods* **19**(1), 31–40.

Gelman, A., Carlin, J., Stern, H., Dunson, D. B., Vehtari, A. & Rubin, D. B. (2013), *Bayesian Data Analysis*, third edn, Chapman and Hall/CRC.

Gibbs, A. L. & Su, F. E. (2002), 'On choosing and bounding probability metrics', *International Statistical Review / Revue Internationale de Statistique* **70**(3), 419–435.

Gut, A. (2013), *Probability: A Graduate Course*, Vol. 75, Springer.

Ledoux, M. & Talagrand, M. (1991), *Probability in Banach Spaces: Isoperimetry and Processes*, Springer-Verlag Berlin Heidelberg, Springer.

Robins, J. M., van der Vaart, A. W. & Ventura, V. (2000), 'Asymptotic Distribution of P Values in Composite Null Models', *Journal of the American Statistical Association* **95**(452), 1143–1156.

Sheehy, A. & Wellner, J. A. (1992), 'Uniform Donsker Classes of Functions', *The Annals of Probability* **20**(4), 1983 – 2030.

Vershynin, R. (2018), *High-Dimensional Probability: An Introduction with Applications in Data Science*, Cambridge Series in Statistical and Probabilistic Mathematics, Cambridge University Press.

Villani, C. (2009), *Optimal transport: old and new*, Vol. 338, Springer.
